# Origin of Optoelectronic Contradictions in 3,4-Cycloalkyl[*c*]-chalcogenophenes: A Computational Study

**DOI:** 10.3390/polym15214240

**Published:** 2023-10-27

**Authors:** Ganesh Masilamani, Gamidi Rama Krishna, Sashi Debnath, Anjan Bedi

**Affiliations:** 1 Department of Chemistry, SRM Institute of Science and Technology, Kattankulathur 603203, India; 2 Organic Chemistry Division, CSIR—National Chemical Laboratory, Pune 411008, India; 3 Department of Radiology, University of Texas Southwestern Medical Center, Dallas, TX 75390, USA

**Keywords:** DFT calculation, chalcogenophene, steric effect, morphology, optoelectronic properties

## Abstract

The planar morphology of the backbone significantly contributes to the subtle optoelectronic features of π-conjugated polymers. On the other hand, the atomistic tuning of an otherwise identical π-backbone could also impact optoelectronic properties systematically. In this manuscript, we compare a series of 3,4-cycloalkylchalcogenophenes by tuning them atomistically using group-16 elements. Additionally, the effect of systematically extending these building blocks in the form of oligomers and polymers is studied. The size of the 3,4-substitution affected the morphology of the oligomers. In addition, the heteroatoms contributed to a further alteration in their geometry and resultant optoelectronic properties. The chalcogenophenes, containing smaller 3,4-cycloalkanes, resulted in lower bandgap oligomers or polymers compared to those with larger 3,4-cycloalkanes. Natural bonding orbital (NBO) calculations were performed to understand the disparity alongside the contour maps of frontier molecular orbitals (FMO).

## 1. Introduction

Chalcogenophenes are planar π-conjugated chromophores that attract significant interest in research because of their widespread presence ranging from device physics to biomedical applications [1,2,3,4]. The amenable modulation of chalcogenophene-based organic materials has guaranteed their significant place in field-effect transistors [5,6,7], organic solar cells [8,9,10], organic light-emitting diodes [11,12], electrochromic switches [13,14] and non-linear optical materials [15] among many other applications. In this process, chalcogenophene-based small molecules, oligomers, cycles, and polymers were synthesized and characterized [16,17]. The synthetic availability of a range of morphology in macromolecular architecture, ranging from homopolymer [18], copolymer [19], block co-polymer [20], or macrocycles [21,22] has given these building blocks a wide range of interest. The tuning of their optoelectronic and device properties has been studied computationally and experimentally in innumerable studies [23,24,25]. Recently, we showed that chalcogenophenes can even induce stronger optical activity in pre-twisted polyaromatic hydrocarbons [26]. Poly-3-hexylthiophene, with a bandgap of 1.99 eV, is soluble in common organic solvents and is considered a benchmark material in device physics [27]. Further substitution on the 3,4-position of thiophene results in 3,4-ethylenedioxy thiophene (EDOT) or 3,4-cyclopenta[*c*]thiophene (**CPS**) and can even result in low-bandgap homopolymers [28,29]. Despite the substitution on the 3,4-position, the π-backbone of poly-cyclopenta[*c*]thiophene (**PCPS**) or poly-cyclopenta[*c*]selenophene (**PCPSe**) was found to be planar-like in poly-3,4-ethylenedioxythiophene (PEDOT) or poly-3,4-ethylenedioxyselenophene (PEDOS) [30,31]. In comparison to PEDOT/PEDOS the **PCPS**/**PCPSe** are oxidatively more stable. Bendikov et al. computationally studied the structure–property relationship of planar chalcogenophene oligomers to find a linear relationship between optoelectronic properties and an increase in oligomer chain length [32]. However, this linear relation could not be expected for the 3,4-cycloalkyl-substituted chalcogenophenes, as steric effects can play an important role in deciding the morphology of their oligomers or homopolymers.

**Figure 1:**
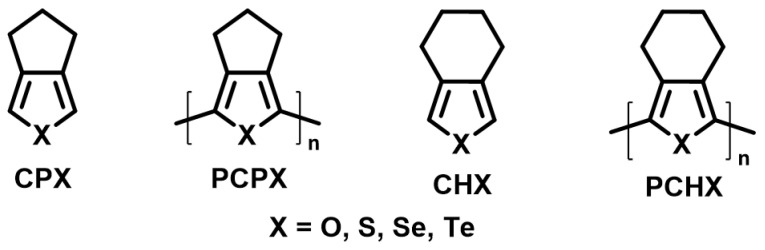
Structure of **CPX**, **PCPX**, **CHX** and **PCHX**.

The synthesis of such 3,4-cycloalkane-fused chalcogenophenes was explored by Fagan et al. [33] and was later followed by Tilley et al. [34]. Rivard et al. extended this technique to make such systems contain Te [35,36]. In this context, Zade et al. extensively synthesized **CPS**- and **CPSe**-based building blocks, polymers, and copolymers [29,31]. **CPS** and **CPSe** are among the building blocks, when polymerized or copolymerized, that produce materials showing a decent-to-excellent charge transport [37,38], electrochromic switching, or which complete the red-green-blue color wheel [39]. While most **CPS**-based polymers were found to be amorphous, copolymers based on **CPS** and bithiazole building blocks showed superior p-type hole mobility arising from the semicrystalline nature of the polymers [37]. In parallel to this, 3,4-cyclopenta[*c*]chalcogenophene(**CPX**)- and 3,4-cyclohexa[*c*]chalcogenophene (**CHX**)-based polymers were also synthesized by Rivard et al. (Figure 1) [40]. The cyclohexa[*c*]tellurophene- and 3-hexylthiophene-based copolymer showed a much higher optical bandgap than the butadiene- and 3-hexylthiophene-based polymer. This indicates that in this polymer, the effect of the heavy atom in lowering the optical bandgap could be overshadowed by the interrupted delocalization of π-electrons caused by the steric effect of 3,4-cyclohexyl substitution on the chalcogenophenes. On the other hand, in all **CPS**-based polymers synthesized by our group, we found a low bandgap. However, the structure of polymer backbones there has been markedly different, leaving no scope for a direct structure–property correlation between the polymers based on **CPX**s and **CHX**s. To our surprise, no study exists to compare them from only an atomistic perspective, either experimentally or computationally.

To observe the atomistic effect on a chromophore toward its optoelectronic properties, chalcogen atoms were chosen as a successful handle [14,40,41,42,43,44]. An alteration in group-16 elements in the backbone of π-conjugated systems provided versatile tools to achieve diverse prospects [45,46]. In addition to the optoelectronic properties, the solid-state packing was influenced heavily by altering the chalcogens, resulting in differential properties in relevant applications [47,48]. **PCPT** and **PCPS** were synthesized electrochemically and compared with their optoelectronic and spectroelectrochemical properties [29,31]. However, the origin of typical optoelectronic features in those with an increasing chain length was never achieved synthetically or computationally from their monomer stage to the polymers via shorter oligomers. Additionally, the furan analog for both **CPX**s and **CHX**s was never achieved experimentally. This inspired us to computationally investigate these two research problems in one attempt. First, we investigated the effect of systematically tuning the heteroatoms in **CPX**s and **CHX**s (where X = O, S, Se, and Te) and their oligomers, which are ***n*CPX**s and ***n*CHX**s (*n* = 2–6) and polymers **(CPX)*_n_*** and **(CHX)*_n_***. Finally, these broader variations of structures provided us with a comparative correlation between their optoelectronic properties and π-delocalization patterns.

## 2. Materials and Methods

### 2.1. Single Crystal X-ray Diffraction (SCXRD)

**(5,5-bis(methoxymethyl)-5,6-dihydro-4H-cyclopenta[*c*]thiophene-1,3-diyl)bis(trime-thylsilane)** was synthesized according to our previous report [29]. SCXRD data were collected at 296 K on Brüker’s KAPPA APEX II CCD Duo (Brüker, Mannheim, Germany) with graphite monochromated Mo-K*α* radiation (0.71073 Å). The crystals were glued to a thin glass fiber using FOMBLIN immersion oil (Aldrich, Delhi, India) and mounted on the diffractometer. Intensity data were processed using Brüker’s suite of data processing programs (SAINT) [49]. The crystal structure was solved through direct methods using SHELXS-97, and the data were refined by full-matrix least-squares refinement on *F*^2^ with anisotropic displacement parameters for non-H atoms using SHELXL-97 [29].

### 2.2. Computational Methodology

All calculations were performed using the density functional theory (DFT) and the Gaussian 16 program package [50]. A computational investigation of the effect of the heteroatom on the optoelectronic properties of the parent and 3,4-cycloalkylchalocogenophenes was performed using the density functional theory (DFT) based on the hybrid function of three parameters Becke-3-Lee-Yang-Parr (B3LYP) [51,52] with an SDD pseudopotential basis set [53,54,55]. Our group extensively analyzed the structure–property relationship of **CPT**- or **CPS**-based systems using functional B3LYP alongside 6-31G(d) as the basis set [29,37]. A computational calculation on chalocogenophene-based small molecules or oligomers is quite reliably correlated to their optoelectronic properties using the DFT-B3LYP-6-31G(d) level for smaller chalcogens [56,57,58]. However, we anticipated a lack of accuracy in finding a potential energy minimum using a 6-31G(d) basis set for the heavier atoms based on several earlier reports [59,60,61]. So, the choice of SDD was made as this effective core potential basis set is known to be useful in circumventing and describing relativistic effects in deep core electrons [62]. Thus, SDD reduced the computational cost and presented reliable optimized geometry and energies of the FMO in our calculations. The computationally obtained gas-phase structure and the crystal structure, derived using the XRD method, for **(5,5-bis(methoxymethyl)-5,6-dihydro-4H-cyclopenta[*c*]thiophene-1,3-diyl)bis(trimethylsilane)** served as a reference point for the choice of our level of calculation. Therefore, to compare the results in a systematic manner, the basis set (SDD) and functional (B3LYP) were kept identical in all calculations for systems containing different heteroatoms. Notably, the head–tail arrangement in ***n*CPX**s or ***n*CHX**s was considered for optimization as it is attributed to higher stability compared to head–head-combined oligomers or polymers [32,63]. The polymers were optimized under the same program package and identical level of theory with an extension of the periodic boundary condition (PBC) [64,65], which was applied to the unit cell constructed on the optimized geometry of the **2CHX** and **2CPX**s. Time-dependent DFT (TDDFT) calculations were performed to understand the electronic transitions in **nCHX**s and **nCPX**s. The monomers and dimers were calculated for 20 independent electronic transitions, whereas this number was 35 for higher oligomers. Natural bonding orbital (NBO) calculations were performed to understand the electron delocalization pathways in the molecules, respectively.

## 3. Results

### 3.1. Crystal Structure

**Figure 2:**
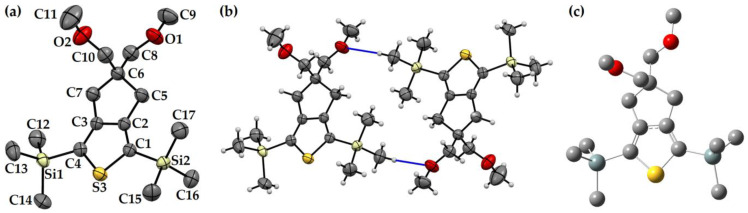
(**a**) Solid-state structure (**b**) Packing and (**c**) Optimized geometry of **(5,5-bis(methoxymethyl)-5,6-dihydro-4H-cyclopenta[*c*]thiophene-1,3-diyl)bis(trimethylsilane)** (CCDC 2249392). Hydrogen atoms were removed from subfigure (**a**,**c**) for clarity. The blue trace in subfigure (**b**) is the van der Waals force (O⋅⋅⋅H = 2.08 Å). Please see supporting information (Figures S1 and S2) for a detailed structure. The ORTEP plot of the molecule was drawn at a 50% probability level.

Finding a general computational method to establish an intuitive structure–property relationship in a series of molecules could be challenging and erroneous without experimental support [66]. In our previous attempts, the characterization of the solid-state geometry of 5,5-bis(methoxymethyl)-5,6-dihydro-4H-cyclopenta[*c*]thiophene was unsuccessful. Also, we strongly anticipate that this compound could afford several intermolecular interactions resulting in large packing forces in the solid state, which could, in turn, produce a substantial difference with gas-phase-optimized geometry. So, we planned to characterize **(5,5-bis(methoxymethyl)-5,6-dihydro-4H-cyclopenta[*c*]thioph-ene-1,3-diyl)bis(trimethylsilane)**, which could afford less π⋅⋅⋅π stacking due to the presence of two sterically demanding trimethylsilyl groups on the 2,5-positions of the thiophene ring (Figure 2). The compound was synthesized according to the previously reported method [29] and was recrystallized from heptane as white needles using the slow evaporation method. The crystal structure was unambiguously characterized using the single-crystal X-ray diffraction (SCXRD) method for the first time. The compound was crystallized in a triclininc space group (P-1), where the unit cell was composed of two molecules bonded to each other through van der Waals forces (O⋅⋅⋅H = 2.08 Å).

In the crystal, the bond angle ∠C1-S3-C4 was 95.4°, whereas, in the optimized (DFT-B3LYP-SDD) structure, it is 92.5°. The difference in bond length between the solid state and gas-phase-optimized geometry is within a difference of 0.02 Å. These negligible alterations in the gas-phase-optimized structure from solid-state geometry further established the choice of DFT-B3LYP-SDD as the method for computational calculation on such molecules. 

### 3.2. Computational Analysis

#### 3.2.1. Gas-Phase Morphology

The planarity of the heterocycles was an eminent feature in both **CPX**s and **CHX**s, which is similar to what has been found earlier in the literature. Interestingly, their oligomers displayed different features in optimized geometry. ***n*CPX**s ranging from the monomer to the hexamer exhibited a planarity of π-conjugated backbone, which is expected for electronic applications where a delocalized planar π-conjugated core effectively influences the optoelectronic properties. By contrast, **CHX**s showed anomalous morphology upon oligomerization. Unlike **CPX**s, **CHX**s exhibited a large dihedral angle between the heterocyclic cores in their gas-phase-optimized structures. This could be attributed to the steric factors of 3,4-cyclohexyl substitution on the chalcogenophene rings. Contextually, PEDOT and PEDOS with a 3,4-ethylenedioxy substitution on chalcogenophene were reported to be planar [30]. This concludes that the extra methylene between the **CPX**s and **CHX**s is exclusively the reason behind the non-planarity of n**CHX**s or **PCHX**.

The heteroatoms also play an important role in the alteration of the geometry within the **CHX** series. For example, in ***n*CHO**s, the noticeable non-planarity of the chains originates at the tetramer (**4CHO**) stage (Table 1), whereas **2CHS** is already substantially non-planar (Table S4). ***n*CHSe** and ***n*CHTe** follow a similar trend (Tables S5 and S6) of affording non-planar morphology at an early stage of oligomerization. Additionally, the change in heteroatoms manifests its effect on the extent of the biaryl twist between chalcogenophene rings. For example, the average biaryl twist angles are 153.4°, 132.9°, 119.5°, and 95.3° for **4CHO**, **4CHS**, **4CHSe**, and **4CHTe**, respectively. On the contrary, the biaryl dihedral angle remained unaltered at 180° for **4CPO**, **4CPS**, **4CPSe**, and **4CPTe**. Therefore, the increased volume of the chalcogenophene rings for larger chalcogen atoms is constantly adjusted within the overall framework of the ***n*CPX**s, whereas it adds to the already existing steric effect of *n***CHX**s. Notably, there was no remarkable increase in the dihedral angle upon the further extension of the **4CHO** oligomer in that series. A similar saturation of the biaryl twist was observed after **2CHS**, **2CHSe**, and **2CHTe** in their corresponding oligomer series.

#### 3.2.2. Optoelectronic Properties

**Figure 3:**
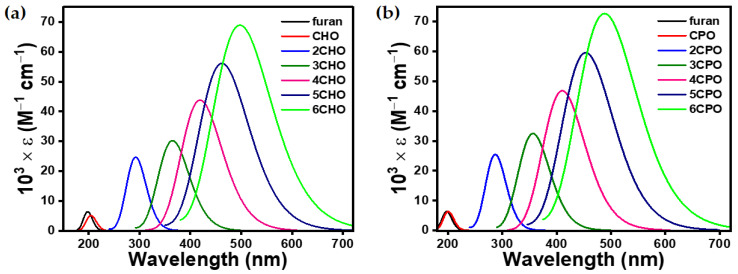
Computationally (TDDFT-B3LYP-SDD) obtained gas-phase UV–vis spectra of (**a**) ***n*CHO** and (**b**) ***n*CPO**. Transitions at a shorter wavelength were omitted for data clarity.

**Figure 4:**
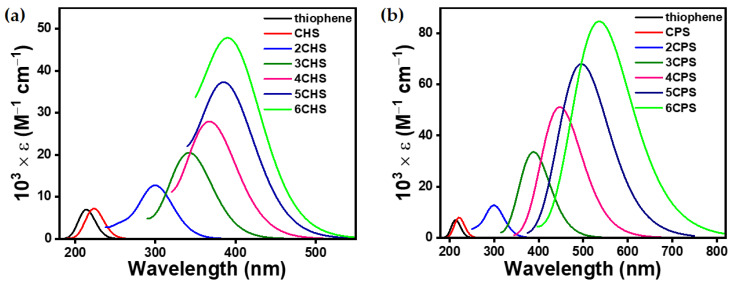
Computationally (TDDFT-B3LYP-SDD) obtained gas-phase UV–vis spectra of (**a**) ***n*CHS** and (**b**) ***n*CPS**.

**Figure 5:**
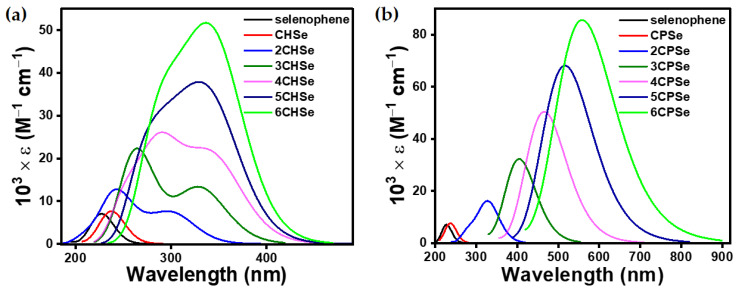
Computationally (TDDFT-B3LYP-SDD) obtained gas-phase UV–vis spectra of (**a**) ***n*CHSe** and (**b**) ***n*CPSe**. Transitions at a shorter wavelength were omitted only for selenophene and **CHSe** for data clarity.

**Figure 6:**
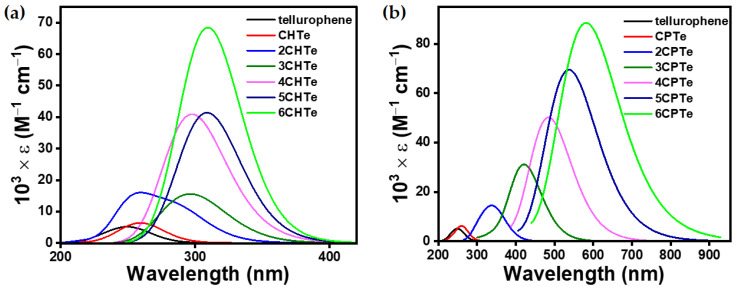
Computationally (TDDFT-B3LYP-SDD) obtained gas-phase UV–vis spectra of (**a**) ***n*CHTe** and (**b**) ***n*CPTe**. Transitions at a shorter wavelength were omitted only for tellurophene and **CHTe** for data clarity.

To achieve a structure–optoelectronic property relationship between the series of ***n*CPX**s and ***n*CHX**s with variable morphological properties, TDDFT calculations were performed under the identical level of calculation, which was used for optimization. The shorter wavelength band in the UV-vis spectra of such molecules is known to arise from several different electronic transitions, whereas the longer band transition is known to be dominated by clear HOMO → LUMO transitions (Figures S7–S20). Therefore, we focused on the longer wavelength transition for a meaningful investigation and unambiguous discussion. However, the complete UV-vis spectra can be found in (Figures S1–S4). The negligible electronic contribution of the aliphatic cyclic 3,4-substitution on the π → π* of chalcogenophene was concluded from the UV-vis spectra (Figure 3, Figure 4, Figure 5 and Figure 6), which showed a small bathochromic shift for mono-**CPX**s and mono-**CHX**s compared to their parent chalcogenophenes (furan, thiophene, selenophene, and tellurophene).

**Figure 7:**
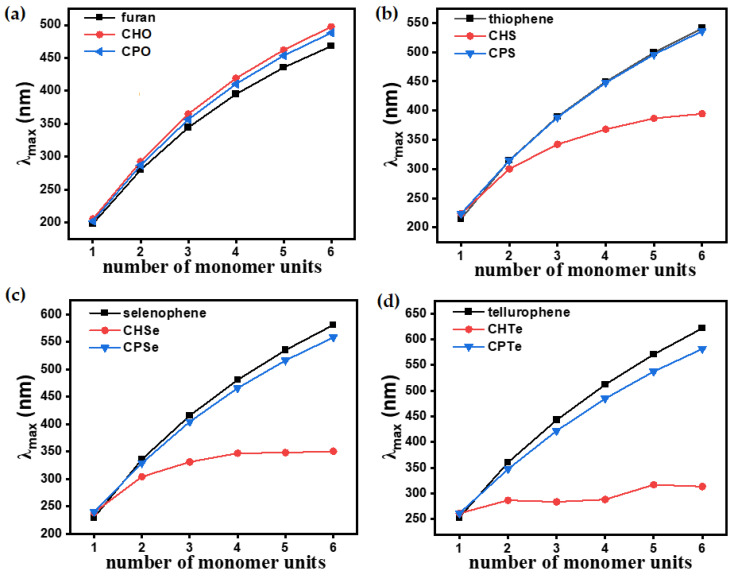
Variation in λ_max_ of (**a**) oligofuran, ***n*CPO** and ***n*CHO**, (**b**) oligothiophene, ***n*CPS** and ***n*CHS**, (**c**) oligoselenophene, ***n*CPSe** and ***n*CHSe**, and (**d**) oligotellurophene, ***n*CPTe** and ***n*CHTe** with the increase oligomers’ length. The black trace is parent chalcogenophenes (furan, thiophene, selenophene and tellurophene), the blue trace is ***n*CPX**s, and red trace is ***n*CHX**s.

The **CPX** and **CHX** monomers showed a distinct π → π* transition in the almost similar position of spectra (Figure 6). However, the extension of **CPX** and **CHX** building blocks to ***n*CPX**s and ***n*CHX**s brings remarkable differences. In the ***n*CPX** series, an increase in the length of oligomers in any series of chalcogenophenes showed a similar trend for the bathochromic shift (Figure 7). For example, the wavelength of absorption maxima (λ_max_) increased from 205 nm for **CPO** to 497 nm for **6CPO** (Figure 7a). This was identical to the extension of the parent furan molecule. ***n*CHX**s also showed similar features of a continuous bathochromic shift to a similar extent via elongation compared to ***n*CPO** or parent furan series (Figure 7b). A remarkable change was observed in the case of the thiophene series. After an almost similar bathochromic shift for the π → π* transition at the dimer stage, the deviation of **3CHS** was discernible compared to the terthiophene or **3CPS**. In the selenophene series, λ_max_ for **nCPSe**s deviated almost negligibly (~5 nm) compared to the oligoselenophenes of similar length, whereas **nCHSe**s deviated from the rest of the congeners at even the dimer stage by 26 nm (Figure 7c). Upon further elongation, the difference between λ_max_ for the ***n*CHSe**s became prominent compared to ***n*CPSe**s or oligoselenophenes of the same length, leading to a saturation in the optical bandgap beyond **4CHSe**. In the tellurophene series, **2CHTe** displayed a substantially low increase in λ_max_ compared to parent bitellurophene and **2CPTe**, which upon further elongation of the chain length, could not yield any conspicuous changes (Figure 7d).

**Figure 8:**
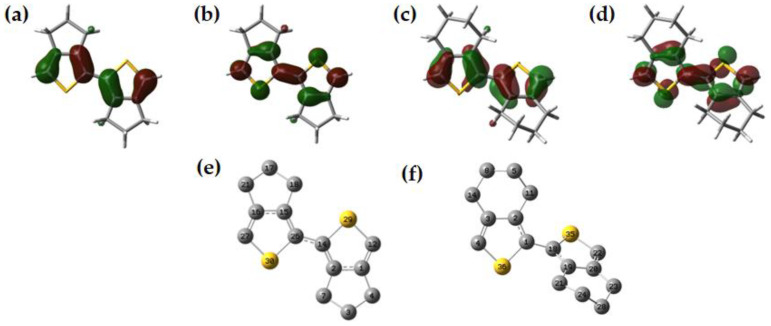
(**a**) Highest occupied molecular orbital (HOMO) and (**b**) lowest unoccupied molecular orbital (LUMO) contour maps of **2CPS**. (**c**) HOMO and (**d**) LUMO contour maps of **2CHS**. The isovalue of 0.04 was considered while generating FMOs. Optimized geometry of (**e**) **2CPS** and (**f**) **2CHS**. Hydrogen atoms were omitted for clarity.

The phenomena of an extension of a π-conjugated building block and an increase in the λ_max_ of the π → π* transition are generally attributed to the extended delocalization of the π-electron system, which converges the HOMO-LUMO gap into a narrower range. However, we found that an increase in *n* for ***n*CHX**s is contradictory to it. This could be due to the lack of π-conjugation, which is triggered by a lack of planar geometry in the higher oligomers compared to those of ***n*CPXs** or the parent chalcogenophenes of a similar chain length. In **CHX**s, the presence of one extra methylene group, compared to **CPX**s, affects the morphology of the polymer chain, impacting effective π-delocalization. On top of that, upon exchanging the heteroatom in the oligomers with the heavier group-16 elements, the already non-planar oligomers further deviated from a suitable geometry for an effective π-overlap of the orbitals toward delocalization. The contour plots of frontier molecular orbitals (FMOs) were compared between **2CHS** and **2CPS** to identify a subtle difference in electron delocalization in both molecules (Figure 8a–d). The covalent bond connecting these two chalcogenophene rings showed a twisted nature of electron density around the connecting bond. This is a clear indication of a disruption in the extension of π-conjugation in the **2CHS** molecule, where the overlapping lobes of p-orbitals move away from each other to a similar extent but in opposite directions.

NBO calculations performed on **2CHT** and **2CPT** provided a clear insight into the lack of electron delocalization between the two rings through the increased dihedral angle. In **2CPT** (Figure 8e), the charge transfer was found between the bonding orbital of C15–C26 (ED_i_ = 0.6839) and C2–C14 (EDj = 0.7296) with a stabilization energy (*E*^2^) of 16.96 kcal/mol) (Figure 8e). Also, C15–C26 (ED_i_ = 0.6839) to C16–C27 (ED_j_ = 0.7315) was identified with an *E*^2^ of 17.47 kcal/mol. These π → π* transitions have a similar stabilization energy of the LP → π* electron transfer as *E*^2^ for LP S29 → where the antibonding NBO of C1–C12 (EDj = 0.7315) is 16.01 kcal/mol. In **2CHS**, *E*^2^ for electron transfer from the LP of S36 to the antibonding NBO of C1–C2 (EDj = 0.6772) was 15.86 kcal/mol and that for the electron transfer between C18 and C19 (ED_i_ = 0.7358) to C1–C2 (ED_j_ = 0.6772) was only 1.85 kcal/mol (Figure 8f). However, major stabilization in the molecule was obtained from the electron transfer between C3 and C4 (EDj = 0.6872) to C1–C2 (EDj = 0.6772) with an *E*^2^ of kcal/mol. This explains that the intra-unit and inter-unit electron delocalization in **2CPS** equally contributed to the stabilization of the molecule in an identical manner to the delocalization of the lone pair of electrons. However, the intra-ring electron delocalization and delocalized lone pair of electrons were found to be the only energy stabilizing factor in **2CHS**, lacking any potential energy stabilization via the inter-unit orbital overlap. This was reciprocated in the typical trend of HOMO-LUMO energies of the oligomers upon increasing the chain length.

**Figure 9:**
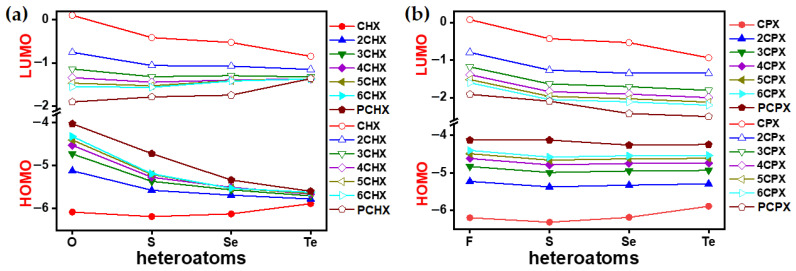
Computationally (DFT-B3LYP-SDD) obtained HOMO–LUMO gap of (**a**) ***n*CHX**s and (**b**) ***n*CPX**s.

The differential trend of energies for FMOs in ***n*CPX**s and ***n*CHX**s needs to be accounted for from two directions, including the (a) atomistic effect and (b) the effect of chain extension (Figure 9). Notably, the stabilization of the HOMO level from furan to thiophene within a series of oligomers of identical length remained a general trend due to the lower aromatic nature of the furan rings than the other chalcogenophenes. In **CHX**s, heavier chalcogens stabilized the HOMO in the monomer in a similar manner to the parent chalcogenophenes. However, from the dimer to the polymer, it showed the stabilization of the HOMO due to less conjugation. The LUMO levels of the monomer and parent chalcogenophene were stabilized with the insertion of heavier chalcogens. However, for *n* ≥ 2, the trends were reversed, resulting in the destabilization of LUMO due to less delocalization. These two antagonistic effects cause an increase in the HOMO-LUMO gap for the ***n*CHX** (n ≥ 2) or **PCHX**s, which further increases upon the insertion of heavier chalcogen atoms. In **CPX**s, the trend of destabilization of the HOMO originates from both the insertion of the heteroatoms and the elongation of the polymer chain. In addition to this, the LUMO levels were stabilized by these factors. These two synergistic effects caused a lowering in the HOMO-LUMO gap in ***n*CPX**s (n ≥ 2) or **PCPX**s.

## 4. Discussion

The systematic comparison between the origin of optoelectronic properties in 3,4-cycloalkyl[*c*]chalcogenophenes was necessary. We did not consider the 3,4-cyclobuta[*c*]chalcogenophene for this purpose for two reasons. First, the 3,4-cyclobutane asserts enormous strain on that heteroaromatic ring, which could force the rehybridization of the orbitals in chalcogenophenes. Finally, the 3,4-cyclobuta[*c*]chalcogenophenes were not synthetically available. However, the **CPX**s and **CHX**s proved to be useful designs for understanding several factors that influence the optoelectronic properties of a π-conjugated building block upon the elongation of chain length. Our study complements previous experimental outcomes of low-bandgap polymers from **CPS-** or **CPSe**-based systems and high-bandgap polymers from **CHX**-based systems. Our earlier reports on the synthesis and characterization of poly-**CPT** support the proof of the concept here as the colorless **CPT** building block, when electropolymerized, showed yellow–orange oligomers and a blue polymer [29]. Also, the excellent electronic properties of polymers arising from planar morphologies were anticipated from our study. Our computational calculation completely supports that observation with a lowering of the bandgap resulting from a higher degree of oligomerization. **CPX**s were proved to be the preferable building blocks where the resulting copolymers or block copolymers were expected to achieve planar morphology and a lower bandgap via electronic delocalization. The electronic effects based on the donor–acceptor intramolecular charge transfer strategy also anticipated the use of **CPX**-based π-systems over **CHX**-based architecture, whereas CHXs could have potential in applications where weakly delocalized covalently bonded π-systems are expected. Block copolymers consisting of **CPX**s and **CHX**s as two different building blocks could significantly attain very different morphology and optoelectronic properties compared to the individual homopolymers, which has synthetically not been achieved yet. **CPTe** carries significant potential in terms of the planar π-conjugated system, phosphorescence [67,68,69], and low bandgap [70,71,72]. 

## 5. Conclusions

We successfully found a path through which optoelectronic contradictions are systematically generated upon an increase in the chain length of covalently bonded **CPX**s and **CHX**s. In **CHX**s, the elongation of the monomer to oligomer and polymer resulted in the planar to twisted morphology of the π-conjugated chain. The optoelectronic properties were affected because of this non-planar morphology and as the HOMO-LUMO gap increased with the extension of the chain length. Heavier heteroatoms further magnified this detrimental effect to result in large bandgap oligomers and polymers. The reason for increasing the disruption in the **CHX**s was attributed to the lack of delocalization of the π-electrons among the covalently bonded building blocks through the help of NBO calculation and HOMO-LUMO contour maps. On the other hand, the oligomer or polymer chains remained planar despite changes in the number of attached building blocks or the insertion of heavier chalcogen atoms in **nCPX**s. As a result, the HOMO-LUMO gap kept on decreasing. **CPX** series showed a remarkable decrease in the HOMO-LUMO gap upon extension. **PCPTe** was found to possess a bandgap wider than that of **PCHTe** by two times. Interesting features could be introduced in alternate, or block copolymers made of **CPX** and **CHX** building blocks. **PCPTe** holds the potential to construct very low-bandgap polymers and, in terms of its anticipated phosphorescence, could be applied in relevant electronic applications. 

## Figures and Tables

**Table 1:** Optimized geometry of the oligomer and polymers of **CPO** and **CHO** at the DFT-B3LYP-SDD level of theory.

*n*CHO	Side View	*n*CPO	Side View
**CHO**	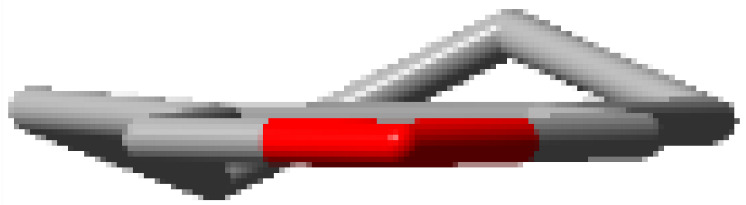	**CPO**	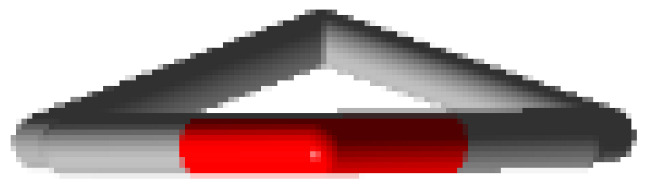
**2CHO**	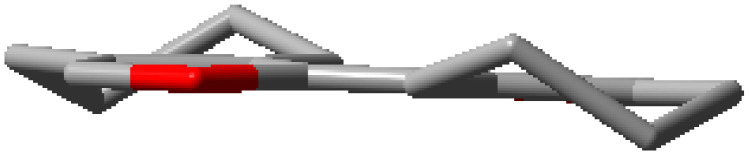	**2CPO**	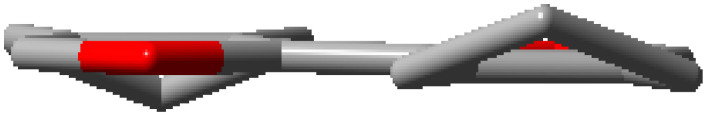
**3CHO**	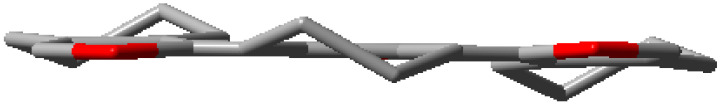	**3CPO**	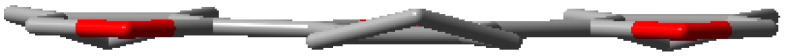
**4CHO**	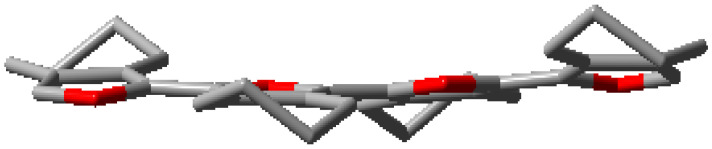	**4CPO**	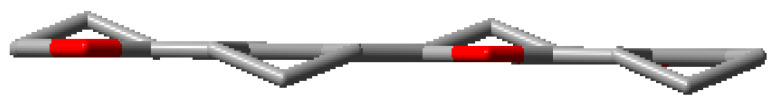
**5CHO**	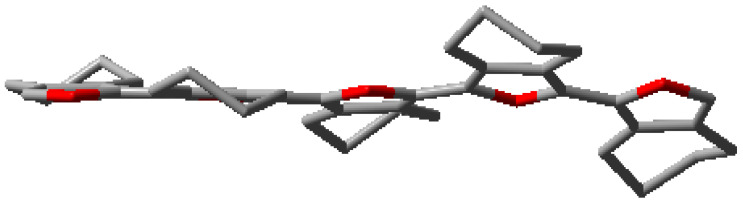	**5CPO**	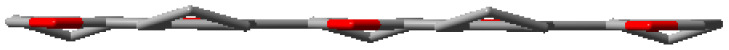
**6CHO**	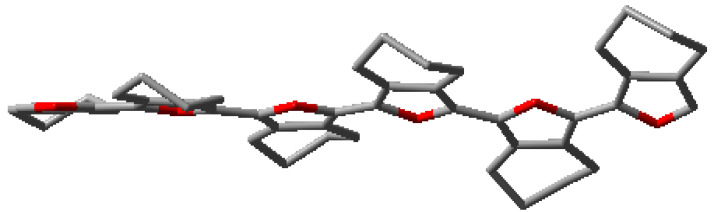	**6CPO**	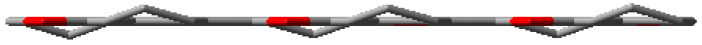
**(CHO)** ***_n_***	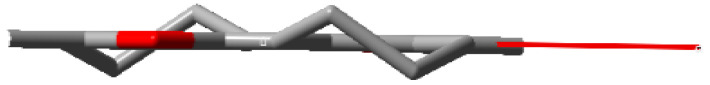	**(CPO)** ***_n_***	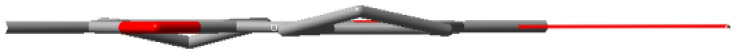

## Data Availability

The data presented in this study are available on request from the corresponding author. The data are not publicly available due to privacy.

## Supplementary Materials

The following supporting information can be downloaded at: https://www.mdpi.com/article/10.3390/polym15214240/s1.
