# Supplementary material for: Origin of Optoelectronic Contradictions in 3,4-Cycloalkyl[c]-chalcogenophenes: A Computational Study
Source: Polymers (Basel). 2023 Oct 27;15(21):4240. doi: 10.3390/polym15214240 (PMC10650045; doi:10.3390/polym15214240)
Supplement: Supplementary file 1 [file polymers-15-04240-s001.zip › polymers-2666297-supplementary.pdf]

Supporting information

Origin of Optoelectronic Contradictions in 3,4-Cycloalkyl[*c*]-chalcogenophenes: A Computational Study

Ganesh Masilamani [1], Gamidi Rama Krishna [2,*], Sashi Debnath [3,*] and Anjan Bedi [1,*]

1 Department of Chemistry, SRM Institute of Science and Technology, Kattankulathur, Chennai 603203, India
2 Organic Chemistry Division, CSIR-National Chemical Laboratory, Pune 411008, India
3 Department of Radiology, University of Texas Southwestern Medical Center, Dallas, TX 75390, USA

Contents

Table S1. Parameters of **(5,5-bis(methoxymethyl)-5,6-dihydro-4H-cyclopenta[c]thiophene -1,3-diyl)bis(trimethylsilane)** obtained from the single crystal X-ray diffraction (SCXRD) technique... 3

Optimized structures ... 6

Table S2. Optimized geometry of the **(5,5-bis(methoxymethyl)-5,6-dihydro-4H-cyclopenta[c]thiophene-1,3-diyl)bis(trimethylsilane)** ... 6

Table S3. Optimized geometry of the Chalcogenophene oligomer of **CHX**s and **CPX**s ... 6

Table S4. Optimized geometry of the oligomer and polymers of **CHS** and **CPS** ... 7

Table S5. Optimized geometry of the oligomer and polymers of **CHSe** and **CPSe** ... 8

Table S6. Optimized geometry of the oligomer and polymers of **CHTe** and **CPTe** ... 9

Computationally (TDDFT-B3LYP-SDD) obtained gas-phase UV−vis spectra ... 10

Figure S3. Gas phase UV−vis spectra of the (a) **CPO** and (b) **CHO** ... 10

Figure S4. Gas phase UV−vis spectra of the (a) **CPS** and (b) **CHS** ... 10

Figure S5. Gas phase UV−vis spectra of the (a) **CPSe** and (b) **CHSe** ... 10

Figure S6. Gas phase UV−vis spectra of the (a) **CPTe** and (b) **CHTe** ... 11

Orbital Contributions ... 11

Table S7. Orbital Contribution of **(5,5-bis(methoxymethyl)-5,6-dihydro-4H-cyclopenta[c]thiophene-1,3-diyl)bis(trimethylsilane)** ... 11

Table S8. Orbital contribution of electronic transitions in chalcogenophene ... 11

Table S9. Orbital contribution of electronic transitions in **CPX** ... 12

Table S10. Orbital contribution of electronic transitions in **2CPX** 12
Table S11. Orbital contribution of electronic transitions in **3CPX** 12
Table S12. Orbital contribution of electronic transitions in **4CPX** 13
Table S13. Orbital contribution of electronic transitions in **5CPX** 13
Table S14. Orbital contribution of electronic transitions in **6CPX** 13
Table S15. Orbital contribution of electronic transitions in **CHX**........ 13
Table S16. Orbital contribution of electronic transitions in **2CHX**........ 14
Table S17. Orbital contribution of electronic transitions in **3CHX**........ 14
Table S18. Orbital contribution of electronic transitions in **4CHX**........ 14
Table S19. Orbital contribution of electronic transitions in **5CHX**........ 15
Table S20. Orbital contribution of electronic transitions in **6CHX**........ 15
Optimized Energy (Hartree) 15
Table S21. Energy of the optimized **(5,5-bis(methoxymethyl)-5,6-dihydro-4H-cyclopenta[c]thiophene-1,3-diyl)bis(trimethylsilane)** 15
Table S22. Energy of the optimized chalcogenophene........ 16
Table S23. Energy of the optimized **CPX** 16
Table S24. Energy of the optimized **2CPX** 16
Table S25. Energy of the optimized **3CPX** 16
Table S26. Energy of the optimized **4CPX** 17
Table S27. Energy of the optimized **5CPX** 17
Table S28. Energy of the optimized **6CPX** 17
Table S29. Energy of the optimized **PCPXs** 17
Table S30. Energy of the optimized **CHXs** 18
Table S31. Energy of the optimized **2CHX**s........ 18
Table S32. Energy of the optimized **3CHX**s........ 18
Table S33. Energy of the optimized **4CHX**s........ 18
Table S34. Energy of the optimized **5CHX**s........ 19
Table S35. Energy of the optimized **6CHX**s........ 19
Table S36. Energy of the optimized **PCHX**s 19
Table S37. Harmonic frequency of the optimized **6CHTe**........ 19
Optimized coordinates of the molecules........ 26
Checkcif report provided by IUCr 147

Table S1. Parameters of **(5,5-bis(methoxymethyl)-5,6-dihydro-4H-cyclopenta[c]thiophene-1,3-diyl)bis(trimethylsilane)** obtained from the single crystal X-ray diffraction (SCXRD) technique

**Empirical formula**	$C_{17}H_{32}O_2SSi_2$
**Formula weight**	356.6708
**Temperature/K**	296(2)
**Crystal system**	triclinic
**Space group**	P-1
**a/Å**	6.0030(4)
**b/Å**	13.0295(10)
**c/Å**	13.9564(10)
**α/°**	101.251(4)
**β/°**	92.850(4)
**γ/°**	91.986(4)
**Volume/Å^3**	1068.21(13)
**Z**	2
**ρ_{calc}g/cm^3**	1.109
**μ/mm^{-1}**	0.268
**F(000)**	388
**Crystal size/mm^3**	0.44 x 0.32 x 0.23
**Radiation**	Mo Kα (λ = 0.71073)
**2θ range for data collection/°**	
**Index ranges**	$-7 \leq h \leq 6$, $-15 \leq k \leq 15$, $-16 \leq l \leq 16$
**Reflections collected**	3764
**Independent reflections**	16158 [R_{int} = 0.0481, R_{sigma} = 0.0414]
**Data/restraints/parameters**	3764/0/207
**Goodness-of-fit on F^2**	1.311
**Final R indexes [I>=2σ (I)]**	R_1 = 0.0720, wR_2 = 0.2057
**Final R indexes [all data]**	R_1 = 0.0855, wR_2 = 0.2104
**Largest diff. peak/hole / e Å^{-3}**	0.390/-0.372
**CCDC number**	2249392

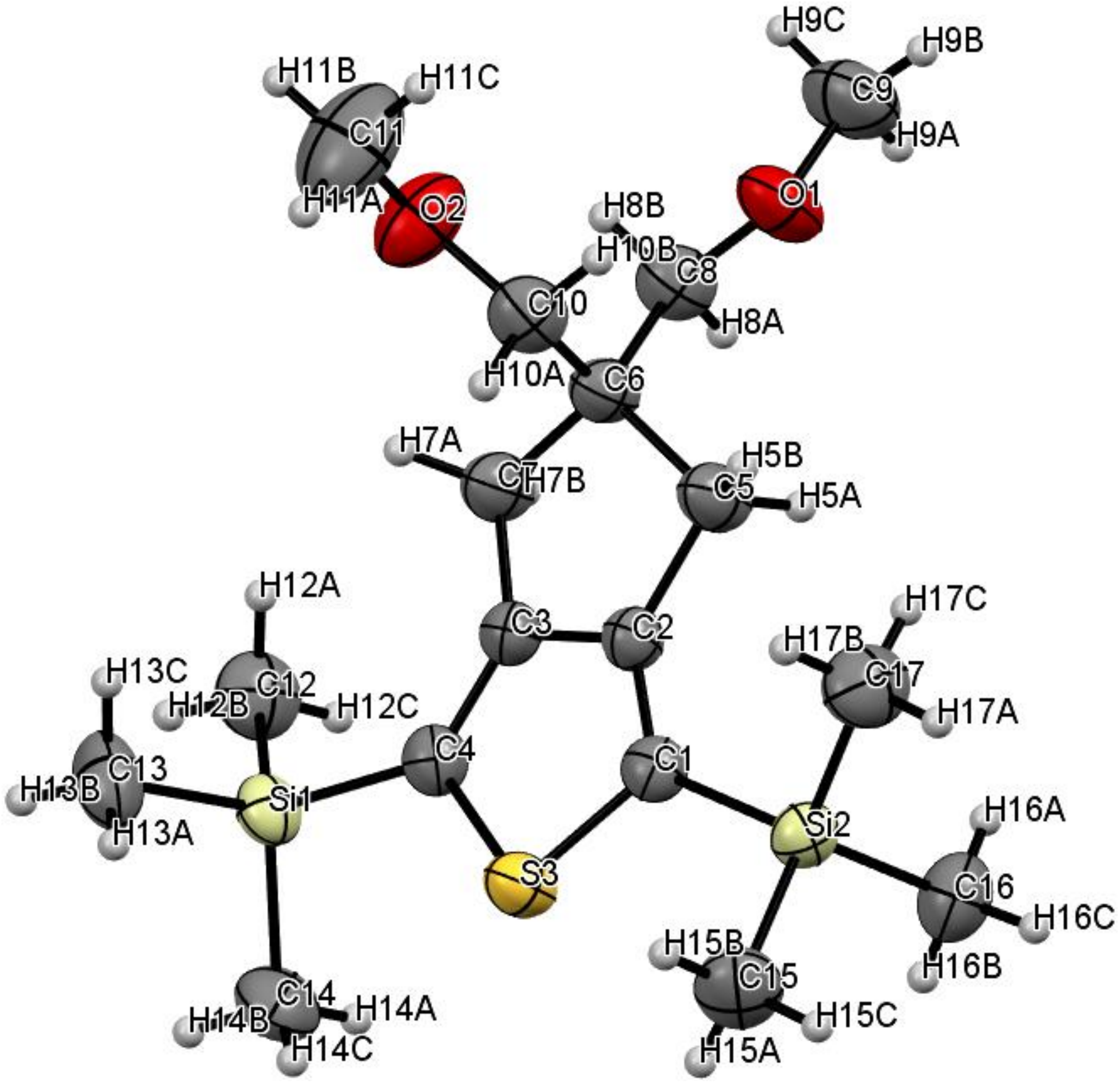

Figure S1. (a) ORTEP representation of **(5,5-bis(methoxymethyl)-5,6-dihydro-4H-cyclopenta[c]thiophene-1,3-diyl)bis(trimethylsilane)**, drawn at 50% probability level.

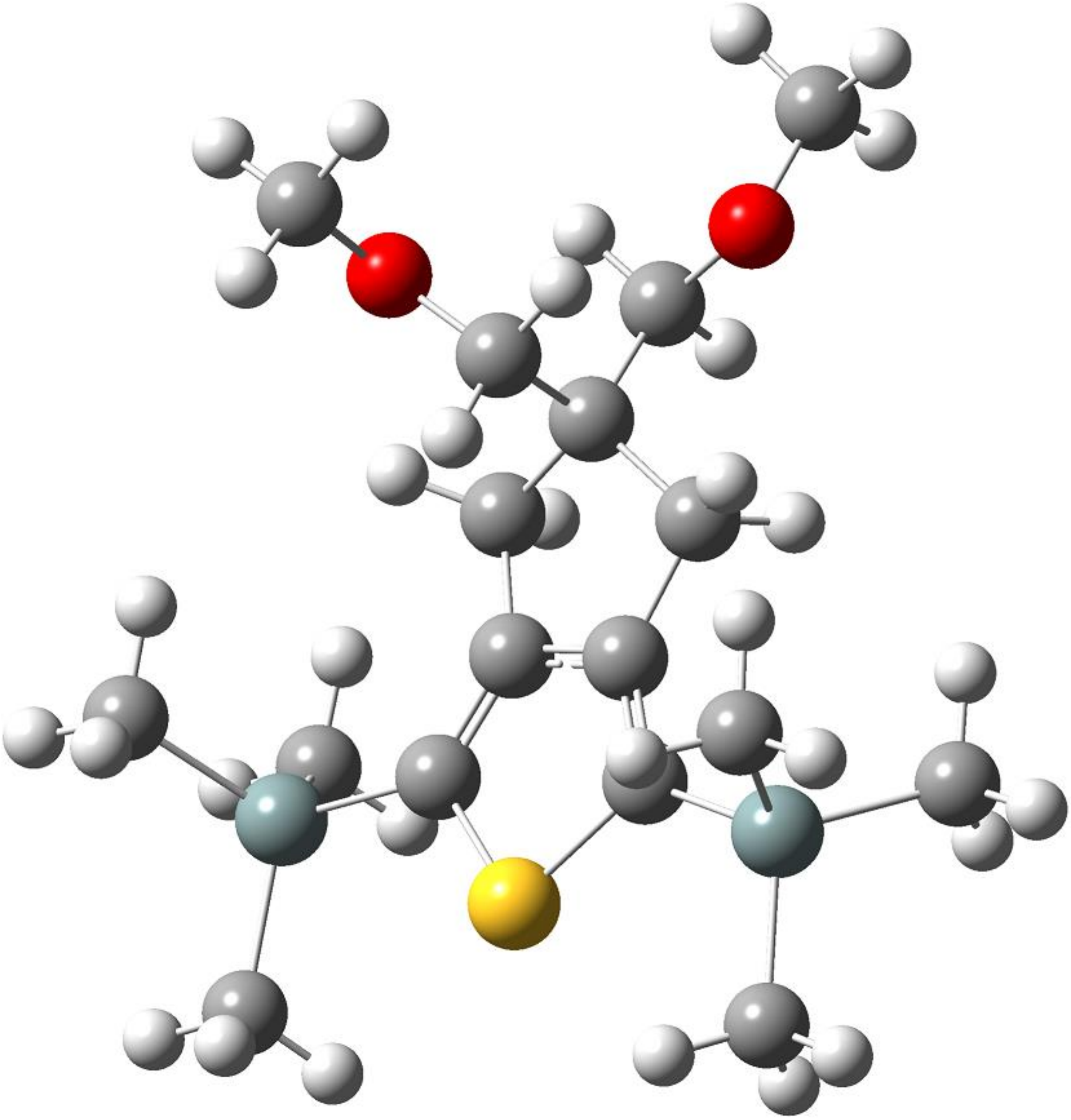

Figure S2. Optimized geometry (DFT-B3LYP-SDD) of **(5,5-bis(methoxymethyl)-5,6-dihydro-4H-cyclopenta[c]thiophene-1,3-diyl)bis(trimethylsilane)**.

Optimized structures

Table S2. Optimized geometry of the **(5,5-bis(methoxymethyl)-5,6-dihydro-4H-cyclopenta[*c*]thiophene-1,3-diyl)bis(trimethylsilane)**

Front View	Side View
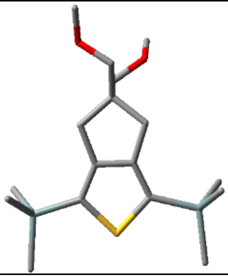	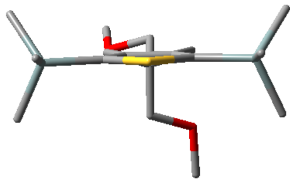

Table S3. Optimized geometry of the Chalcogenophene oligomer of **CHX**s and **CPX**s

**CHX**s	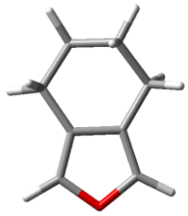	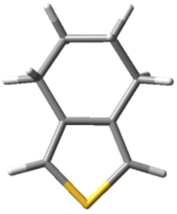	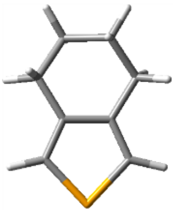	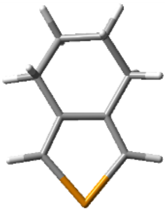
**CPX**s	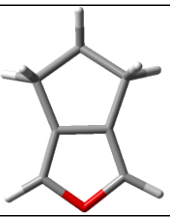	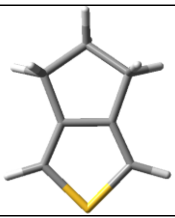	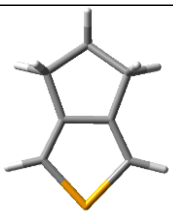	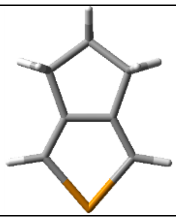

Table S4. Optimized geometry of the oligomer and polymers of **CHS** and **CPS**

	Front View	Side View		Front View	Side View
**CHS**	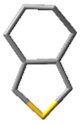	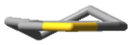	**CPS**	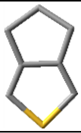	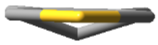
**2CHS**	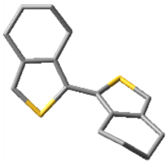	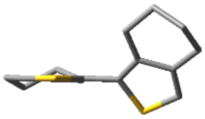	**2CPS**	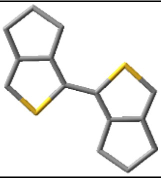	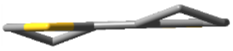
**3CHS**	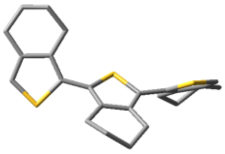	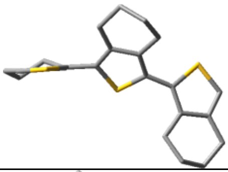	**3CPS**	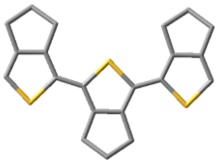	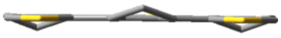
**4CHS**	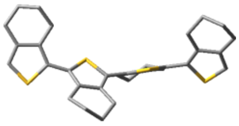	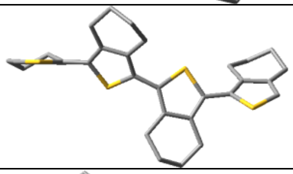	**4CPS**	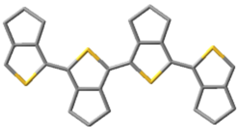	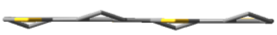
**5CHS**	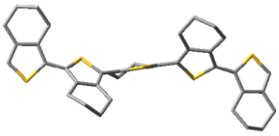	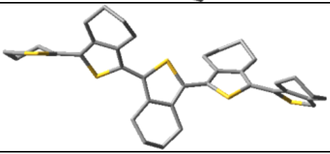	**5CPS**	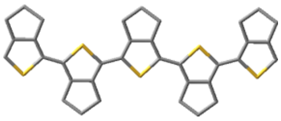	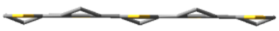
**6CHS**	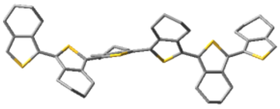	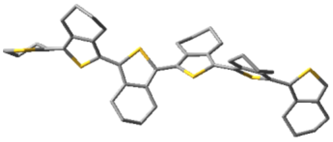	**6CPS**	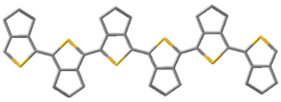	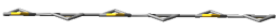
**PCHS**	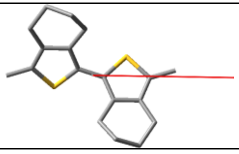	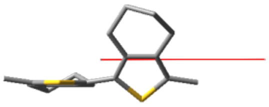	**PCPS**	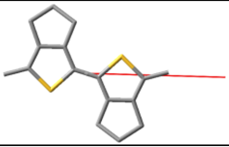	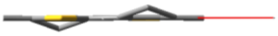

Table S5. Optimized geometry of the oligomer and polymers of **CHSe** and **CPSe**

	Front View	Side View		Front View	Side View
**CHSe**	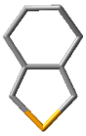	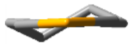	**CPSe**	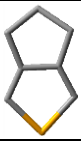	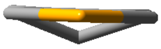
**2CHSe**	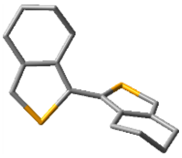	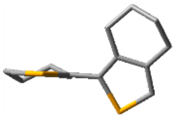	**2CPSe**	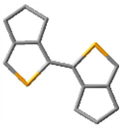	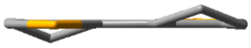
**3CHSe**	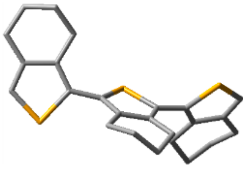	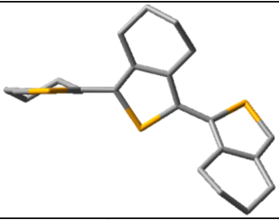	**3CPSe**	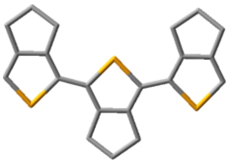	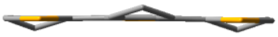
**4CHSe**	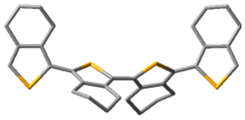	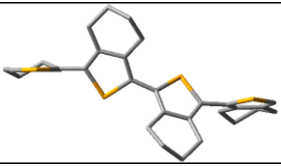	**4CPSe**	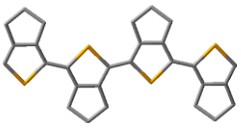	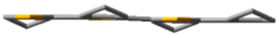
**5CHSe**	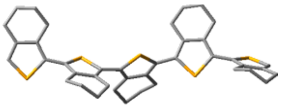	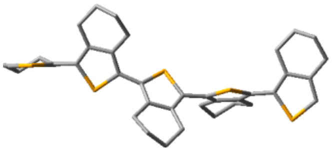	**5CPSe**	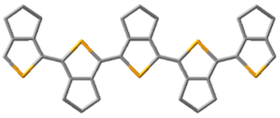	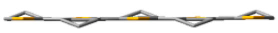
**6CHSe**	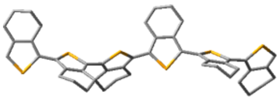	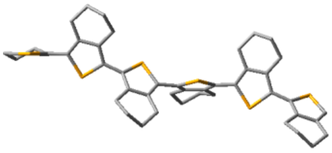	**6CPSe**	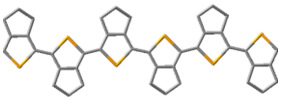	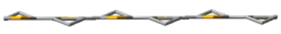
**PCHSe**	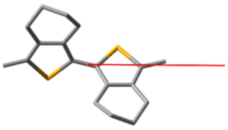	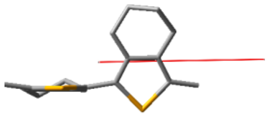	**PCPSe**	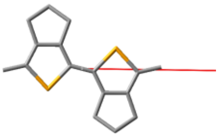	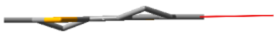

Table S6. Optimized geometry of the oligomer and polymers of **CHTe** and **CPTe**

	Front View	Side View		Front View	Side View
**CHTe**	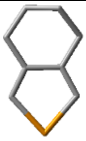	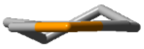	**CPTe**	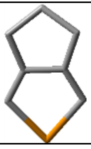	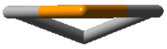
**2CHTe**	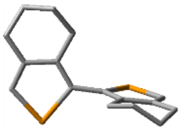	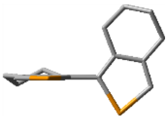	**2CPTe**	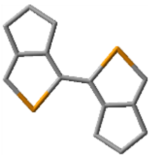	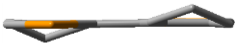
**3CHTe**	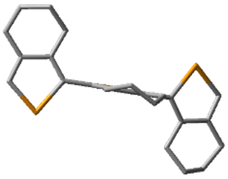	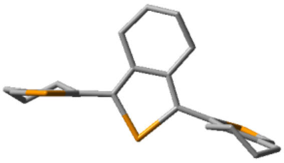	**3CPTe**	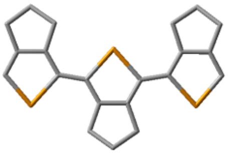	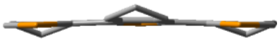
**4CHTe**	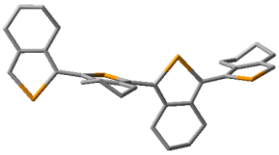	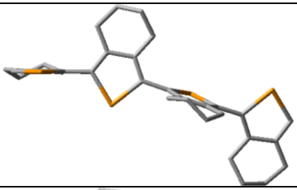	**4CPTe**	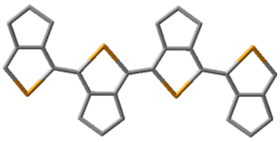	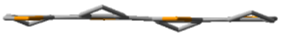
**5CHTe**	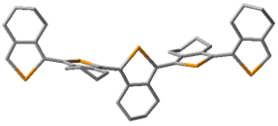	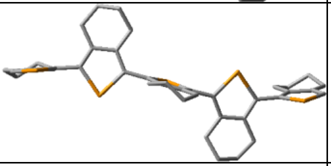	**5CPTe**	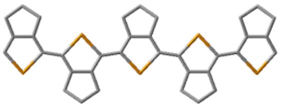	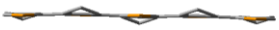
**6CHTe**	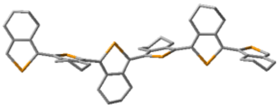	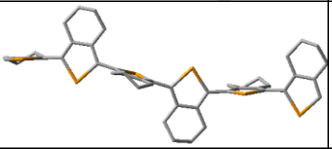	**6CPTe**	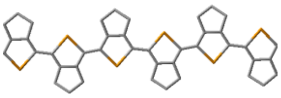	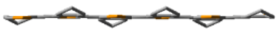
**PCHTe**	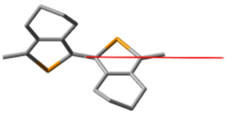	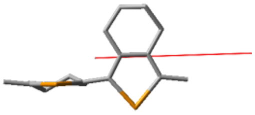	**PCPTe**	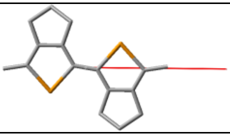	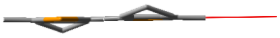

Computationally (TDDFT-B3LYP-SDD) obtained gas-phase UV−vis spectra

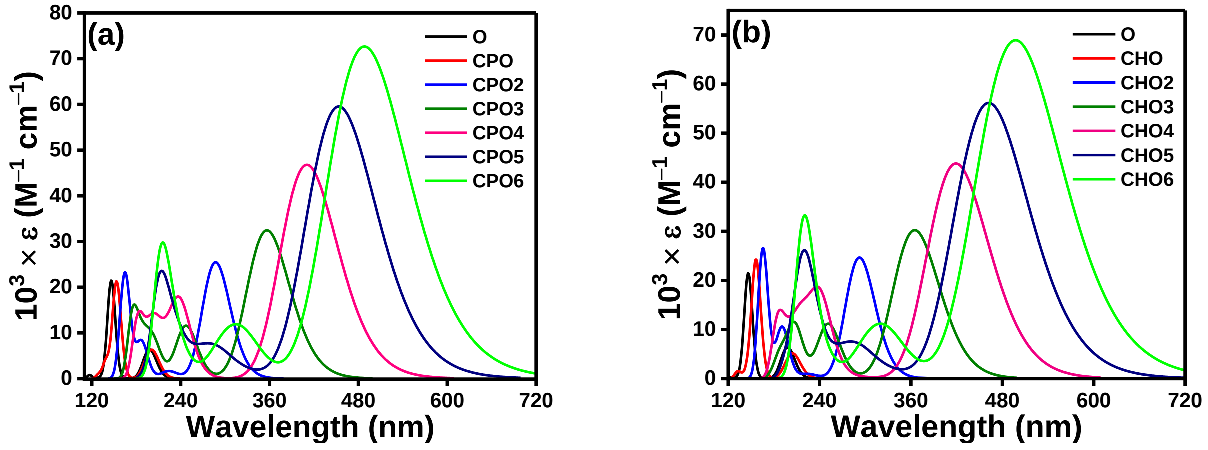

Figure S3. Gas phase UV−vis spectra of the (a) **CPO** and (b) **CHO**

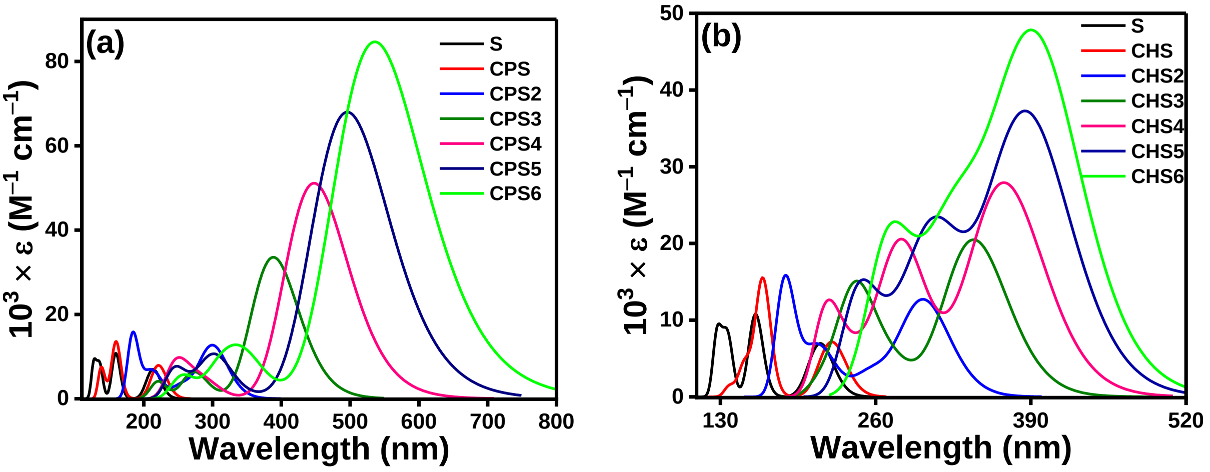

Figure S4. Gas phase UV−vis spectra of the (a) **CPS** and (b) **CHS**

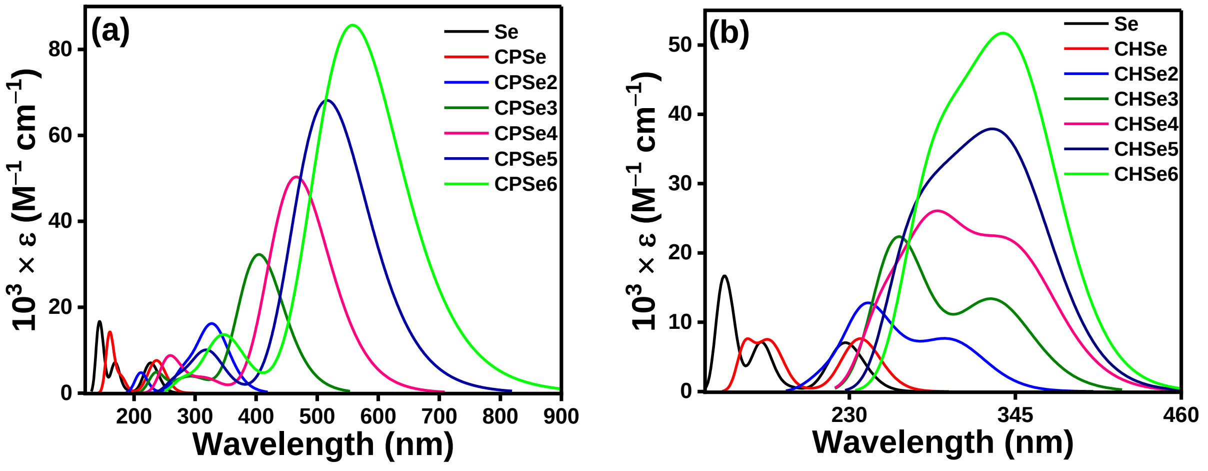

Figure S5. Gas phase UV−vis spectra of the (a) **CPSe** and (b) **CHSe**

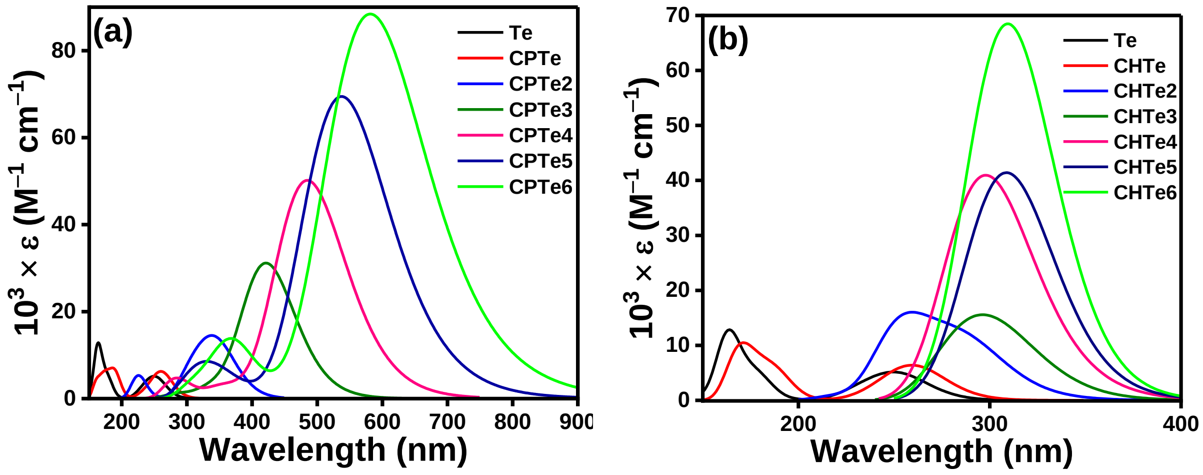

Figure S6. Gas phase UV−vis spectra of the (a) **CPTe** and (b) **CHTe**

Orbital Contributions

Table S7. Orbital Contribution of **(5,5-bis(methoxymethyl)-5,6-dihydro-4H-cyclopenta[c]thiophene-1,3-diyl)bis(trimethylsilane)**

Compound	State number	Oscillator Strength (*f*)	Transition energy (eV)	Wavelength (nm)	Major transitions	
					Orbitals involved	Contri-bution (%)
**(5,5-bis(methoxymethyl)-5,6-dihydro-4H-cyclopenta[c]thiophene-1,3-diyl)bis(trimethylsilane)**	1	0.3164	5.0809	244.02	H→L	95.81

Table S8. Orbital contribution of electronic transitions in chalcogenophene

Compounds	State	Oscillator Strength (*f*)	Transition energy (eV)	Wavelength (nm)	Major transitions			
					Orbitals involved	Contri-bution (%)	Orbitals involved	Contri-bution (%)
**Furan**	1	0.1567	6.2605	198.04	H→L	98.47	-	-
	5	0.4501	8.4232	147.19	H→L+1	57.82	H-1→L	38.42
**Thiophene**	1	0.1005	5.7780	214.58	H→L	95.62	H-2→L+1	2.11
	7	0.1885	7.7512	159.95	H→L+2	71.64	H-3→L	15.98
	16	0.2066	9.7285	127.44	H-2→L+1	85.56	H-5→L+1	5.0
**Selenophene**	1	0.1022	5.3899	233.68	H-1→L	90.50	H→L+4	6.19
	10	0.0755	7.2740	170.45	H-1→L+3	84.45	H→L+4	12.43
	20	0.2833	8.7996	140.90	H-3→L	59.05	H→L+4	20.00

**Tellurophene**	3	0.1063	4.8888	253.61	H→L+1	92.40	H-1→L+1	3.12
	10	0.2670	7.5686	177.77	H→L+5	43.02	H-2→L	41.48

Table S9. Orbital contribution of electronic transitions in **CPX**

**Compounds**	**State**	**Oscillator Strength (*f*)**	**Transition energy (eV)**	**Wavelength (nm)**	**Major transitions**			
					**Orbitals involved**	**Contri-bution (%)**	**Orbitals involved**	**Contri-bution (%)**
**CPO**	1	0.1302	6.1379	202.00	H→L	98.54	-	-
	5	0.3289	8.0285	154.43	H→L+1	50.07	H→L+3	17.05
	13	0.0175	8.8876	139.50	H-1→L+3	90.35	H→L+5	4.5
**CPS**	1	0.1196	5.5420	223.72	H-1→L	90.04	H→L+2	8.01
	2	0.0817	5.6829	218.17	H→L	95.74	-	-
	9	0.1610	7.6698	161.65	H→L+2	56.62	H-3→L	29.83
	10	0.1421	7.9206	156,53	H-3→L	64.01	H→L+2	22.91
	18	0.1320	9.0219	137.43	H-6→L	76.66	H-8→L	10.19
**CPSe**	3	0.1459	5.1632	240.13	H→L	92.17	H-1→L+5	4.80
	8	0.0492	6.8785	180.25	H→L+3	97.05	-	-
	18	0.2033	7.8119	158.71	H-1→L+5	43.01	H-3→L	34.76
**CPTe**	3	0.1459	4.7390	261.62	H→L+1	93.30	H-1→L+5	2.78
	10	0.0589	6.5352	189.72	H→L+3	97.26	-	-
	15	0.0997	7.1394	173.66	H→L+5	82.90	H-2→L	5.70
	20	0.0798	7.7258	160.48	H-1→L+5	42.06	H-6→L+1	18.99

Table S10. Orbital contribution of electronic transitions in **2CPX**

**Compound Name**	**State**	**Oscillator Strength (*f*)**	**Transition energy (eV)**	**Wavelength (nm)**	**Major transitions**	
					**Orbitals involved**	**Contribution (%)**
**2CPO**	1	0.6287	4.3176s	287.16	H→L	100
**2CPS**	1	0.4634	3.9413	314.58	H→L	98.50
	3	0.1110	4.7044	263.55	H-1→L	94.00
**2CPSe**	1	0.3852	3.7657	329.25	H→L	95.94
	4	0.1341	4.3798	283.08	H-1→L	93.50
**2CPTe**	2	0.2949	3.5688	347.41	H→L	88.75
	7	0.1658	4.0098	309.20	H-1→L	88.20

Table S11. Orbital contribution of electronic transitions in **3CPX**

**Compound Name**	**State**	**Oscillator Strength (*f*)**	**Transition energy (eV)**	**Wavelength (nm)**	**Major transitions**	
					**Orbitals involved**	**Contribution (%)**
**3CPO**	1	0.8012	3.4777	356.51	H→L	100
**3CPS**	1	0.8286	3.1920	388.42	H→L	100

**3CPSe**	1	0.7969	3.0641	404.63	H→L	99.38
**3CPTe**	1	0.7628	2.9380	422.00	H→L	98.14

Table S12. Orbital contribution of electronic transitions in **4CPX**

**Compound Name**	**State**	**Oscillator Strength (*f*)**	**Transition energy (eV)**	**Wavelength (nm)**	**Major transitions**	
					**Orbitals involved**	**Contribution (%)**
**4CPO**	1	1.1551	3.0207	410.45	H→L	100
**4CPS**	1	1.2625	2.7702	447.56	H→L	100
**4CPSe**	1	1.2429	2.6618	465.80	H→L	99.96
**4CPTe**	1	1.2382	2.5571	484.86	H→L	99.42

Table S13. Orbital contribution of electronic transitions in **5CPX**

**Compound Name**	**State**	**Oscillator Strength (*f*)**	**Transition energy (eV)**	**Wavelength (nm)**	**Major transitions**	
					**Orbitals involved**	**Contribution (%)**
**5CPO**	1	1.4700	2.7339	453.51	H→L	100
**5CPS**	1	1.6787	2.4997	495.99	H→L	100
**5CPSe**	1	1.6827	2.4014	516.30	H→L	100
**5CPTe**	1	1.7148	2.3071	537.40	H→L	99.71

Table S14. Orbital contribution of electronic transitions in **6CPX**

**Compound Name**	**State**	**Oscillator Strength (*f*)**	**Transition energy (eV)**	**Wavelength (nm)**	**Major transitions**	
					**Orbitals involved**	**Contribution (%)**
**6CPO**	1	1.7940	2.5399	488.14	H→L	99.96
**6CPS**	1	2.0902	2.3135	535.92	H→L	99.96
**6CPSe**	1	2.1151	2.2211	558.21	H→L	99.84
**6CPTe**	1	2.1831	2.1323	581.47	H→L	99.65

Table S15. Orbital contribution of electronic transitions in **CHX**

**Compound Name**	**State**	**Oscillator Strength (*f*)**	**Transition energy (eV)**	**Wavelength (nm)**	**Major transitions**			
					**Orbitals involved**	**Contri-bution (%)**	**Orbitals involved**	**Contri-bution (%)**
**CHO**	1	0.1156	6.0365	205.39	H→L	98.63	-	-
	5	0.5026	7.8876	157.19	H→L+1	64.64	H-1→L	24.35
**CHS**	1	0.1062	5.5450	223.60	H-1→L	88.43	H→L+2	9.61
	9	0.2848	7.4908	165.51	H→L+2	76.66	H-6→L	8.48
	13	0.1046	8.2328	150.60	H-6→L	86.39	H→L+2	7.35
	18	0.0235	9.0070	137.65	H-2→L+1	96.54	-	-

**CHSe**	3	0.1257	5.1888	238.94	H-1→L	75.40	H-1→L+1	16.48
	14	0.0733	7.2372	171.32	H→L+5	60.07	H-5→L	24.98
	18	0.0692	7.8237	158.47	H-2→L+1	82.66	H-6→L	11.49
**CHTe**	3	0.1419	4.7416	261.48	H→L+1	90.48	H-1→L+5	3.01
	19	0.1041	7.4198	167.10	H-5→L	56.91	H→L+5	14.46

Table S16. Orbital contribution of electronic transitions in **2CHX**

**Compound Name**	**State**	**Oscillator Strength (*f*)**	**Transition energy (eV)**	**Wavelength (nm)**	**Major transitions**			
					**Orbitals involved**	**Contri-bution (%)**	**Orbitals involved**	**Contri-bution (%)**
**2CHO**	1	0.6088	4.2402	292.40	H→L	100	-	-
**2CHS**	1	0.3098	4.1287	300.30	H→L	96.94	-	-
	4	0.0676	4.8350	256.43	H-2→L	93.19	-	-
**2CHSe**	1	0.1552	4.0725	304.44	H→L	90.60	-	-
	4	0.0510	4.4875	276.29	H-1→L	83.00	H-2→L+1	13.00
**2CHTe**	8	0.1456	4.3235	286.77	H-3→L+1	33.59	H-1→L	27.26
	14	0.1345	4.8469	255.80	H-1→L+3	50.07	H-2→L+3	20.43
	16	0.0044	4.9393	251.01	H-3→L+3	66.46	H-2→L+2	13.89

Table S17. Orbital contribution of electronic transitions in **3CHX**

**Compound Name**	**State**	**Oscillator Strength (*f*)**	**Transition energy (eV)**	**Wavelength (nm)**	**Major transitions**			
					**Orbitals involved**	**Contri-bution (%)**	**Orbitals involved**	**Contri-bution (%)**
**3CHO**	1	0.7466	3.3976	364.92	H→L	100	-	-
**3CHS**	1	0.5044	3.6227	342.24	H→L	99.37	-	-
**3CHSe**	1	0.3126	3.7427	331.27	H→L	94.93	-	-
**3CHTe**	8	0.0867	3.9617	312.96	H-1→L	36.77	H-3→L	27.80
	12	0.0076	4.1488	298.85	H-3→L+1	38.93	H→L+2	11.06
	19	0.0019	4.3570	284.56	H-5→L+3	27.08	H-3→L+3	16.32

Table S18. Orbital contribution of electronic transitions in **4CHX**

**Compound Name**	**State**	**Oscillator Strength (*f*)**	**Transition energy (eV)**	**Wavelength (nm)**	**Major transitions**			
					**Orbitals involved**	**Contri-bution (%)**	**Orbitals involved**	**Contri-bution (%)**
**4CHO**	1	1.0819	2.9587	419.05	H→L	100	-	-
**4CHS**	1	0.6838	3.3692	367.99	H→L	98.96	-	-
**4CHSe**	1	0.4491	3.5709	347.21	H→L	96.83	-	-
**4CHTe**	14	0.1581	4.0034	309.70	H-1→L+1	27.59	H→L	15.74
	20	0.1599	4.1361	299.76	H-1→L+3	29.19	H-2→L+2	13.94
	27	0.3616	4.2999	288.34	H-5→L+3	19.90	H-4→L+2	16.74

Table S19. Orbital contribution of electronic transitions in **5CHX**

Compound Name	State	Oscillator Strength (f)	Transition energy (eV)	Wavelength (nm)	Major transitions			
					Orbitals involved	Contri-bution (%)	Orbitals involved	Contri-bution (%)
**5CHO**	1	1.3866	2.6831	462.09	H→L	100	-	-
**5CHS**	1	0.9036	3.2053	386.81	H→L	98.82	-	-
**5CHSe**	1	0.5955	3.5595	348.32	H→L	96.56	-	-
	4	0.2404	3.8320	323.55	H-1→L+1	64.62	H-2→L	10.65
	8	0.2634	4.1393	299.53	H→L+2	40.21	H-2→L	26.29
**5CHTe**	15	0.2337	3.9098	317.11	H-1→L+1	35.47	-	-
	19	0.1655	4.0067	309.44	H-1→L+3	29.19	-	-
	29	0.1940	4.1437	299.21	H-5→L+3	24.04	H-6→L+1	15.69

Table S20. Orbital contribution of electronic transitions in **6CHX**

Compound Name	State	Oscillator Strength (f)	Transition energy (eV)	Wavelength (nm)	Major transitions			
					Orbitals involved	Contri-bution (%)	Orbitals involved	Contri-bution (%)
**6CHO**	1	1.7021	2.4927	497.38	H→L	99.92	-	-
**6CHS**	1	1.1183	3.1409	394.74	H→L	97.53	-	-
**6CHSe**	1	0.8661	3.5351	350.72	H→L	91.60	-	-
	8	0.3791	3.9880	310.89	H-2→L+2	26.94	H→L+2	20.72
	24	0.3018	4.3761	283.32	H-2→L+2	32.46	H-1→L+3	17.21
**6CHTe**	22	0.3470	3.9534	313.61	H-6→L+1	18.87	H-1→L+2	16.69
	25	0.3261	4.0175	308.61	H-5→L+2	10.86	H-2→L	10.77
	31	0.3059	4.0763	304.16	H-9→L+3	21.85	H→L+3	20.22

Optimized Energy (Hartree)

Table S21. Energy of the optimized **(5,5-bis(methoxymethyl)-5,6-dihydro-4H-cyclopenta[c]thiophene-1,3-diyl)bis(trimethylsilane)**

Compound	Energy (Hartree)
**(5,5-bis(methoxymethyl)-5,6-dihydro-4H-cyclopenta[c]thiophene-1,3-diyl)bis(trimethylsilane)**	-1794.5837070

Table S22. Energy of the optimized chalcogenophene

Compounds	Energy (Hartree)
**Furan**	-229.9860492
**Thiophene**	-552.9455852
**Selenophene**	-164.1228203
**Tellurophene**	-162.8486085

Table S23. Energy of the optimized **CPX**

Compounds	Energy (Hartree)
**CPO**	-346.6962664
**CPS**	-669.6609078
**CPSe**	-280.8391428
**CPTe**	-279.5664412

Table S24. Energy of the optimized **2CPX**

Compounds	Energy (Hartree)
**2CPO**	-692.2093466
**2CPS**	-1338.1331951
**2CPSe**	-560.4894701
**2CPTe**	-557.9404176

Table S25. Energy of the optimized **3CPX**

Compounds	Energy (Hartree)
**3CPO**	-1037.7229926
**3CPS**	-2006.6063009
**3CPSe**	-840.1407251
**3CPTe**	-836.315022

Table S26. Energy of the optimized **4CPX**

Compounds	Energy (Hartree)
**4CPO**	-1383.2368772
**4CPS**	-2675.0796139
**4CPSe**	-1119.7921924
**4CPTe**	-1114.6897604

Table S27. Energy of the optimized **5CPX**

Compounds	Energy (Hartree)
**5CPO**	-1728.7507897
**5CPS**	-3343.552938
**5CPSe**	-1399.4436633
**5CPTe**	-1393.0645297

Table S28. Energy of the optimized **6CPX**

Compounds	Energy (Hartree)
**6CPO**	-2074.2647228
**6CPS**	-4012.0263003
**6CPSe**	-1679.0951668
**6CPTe**	-1671.4393181

Table S29. Energy of the optimized **PCPXs**

Compounds	Energy (Hartree)
**PCPO**	-691.0278481
**PCPS**	-1337.1169193
**PCPSe**	-559.3029959
**PCPTe**	-556.7496582

Table S30. Energy of the optimized **CHXs**

Compounds	Energy (Hartree)
**CHO**	-386.0166933
**CHS**	-708.976755
**CHSe**	-320.1535886
**CHTe**	-318.8788132

Table S31. Energy of the optimized **2CHX**s

Compounds	Energy (Hartree)
**2CHO**	-770.8499267
**2CHS**	-1416.7607782
**2CHSe**	-639.1141401
**2CHTe**	-636.5627672

Table S32. Energy of the optimized **3CHX**s

Compounds	Energy (Hartree)
**3CHO**	-1155.6835761
**3CHS**	-2124.545092
**3CHSe**	-958.0749552
**3CHTe**	-954.2467532

Table S33. Energy of the optimized **4CHX**s

Compounds	Energy (Hartree)
**4CHO**	-1540.5175324
**4CHS**	-2832.3293363
**4CHSe**	-1277.0357326
**4CHTe**	-1271.9307992

Table S34. Energy of the optimized **5CHX**s

Compounds	Energy (Hartree)
**5CHO**	-1925.3515061
**5CHS**	-3540.1136015
**5CHSe**	-1595.9965555
**5CHTe**	-1589.6148387

Table S35. Energy of the optimized **6CHX**s

Compounds	Energy (Hartree)
**6CHO**	-2310.1854927
**6CHS**	-4247.8978621
**6CHSe**	-1914.9573536
**6CHTe**	-1907.2988719

Table S36. Energy of the optimized **PCHX**s

Compounds	Energy (Hartree)
**PCHO**	-769.6677978
**PCHS**	-1415.7558195
**PCHSe**	-637.9143631
**PCHTe**	-635.3637404

Table S37. Harmonic frequency of the optimized **6CHTe**

Mode	Harmonic frequency (cm^{-1})
1	4.648
2	5.2846
3	8.973
4	12.6446
5	12.9869
6	18.1884
7	23.7461

8	26.3202
9	34.3268
10	46.0878
11	50.9739
12	60.0452
13	75.4951
14	93.5984
15	93.7519
16	106.6511
17	108.9316
18	112.9711
19	124.4556
20	129.4895
21	135.0162
22	135.8753
23	139.562
24	140.954
25	143.6055
26	145.4383
27	148.2999
28	149.7118
29	150.2891
30	156.7138
31	156.8239
32	162.6508
33	177.7205
34	190.1736
35	203.9882
36	215.7206
37	219.8629
38	219.9361
39	224.9402
40	228.018
41	232.7737
42	235.4584
43	240.7707
44	264.2509
45	290.6696
46	299.6846
47	301.0133
48	303.2524

49	306.7021
50	315.2338
51	323.7485
52	329.6963
53	339.0434
54	358.4723
55	368.6929
56	383.0582
57	410.9383
58	413.409
59	444.7227
60	451.2648
61	468.3712
62	495.7589
63	528.958
64	557.4061
65	580.2214
66	597.2052
67	613.4995
68	618.3461
69	618.4412
70	621.1028
71	631.3299
72	633.0717
73	636.2156
74	645.1141
75	645.3117
76	653.8399
77	653.8985
78	659.8238
79	671.8498
80	677.1244
81	687.9782
82	697.0135
83	697.4035
84	699.4081
85	700.5068
86	701.7914
87	703.6263
88	709.4865
89	710.9996

90	727.2971
91	752.284
92	760.4828
93	762.722
94	776.3449
95	828.4356
96	846.3795
97	855.391
98	859.3232
99	860.7127
100	875.5495
101	875.6406
102	883.2181
103	891.3549
104	899.6663
105	905.8278
106	908.0567
107	908.3241
108	917.182
109	919.1747
110	921.1895
111	922.4265
112	935.4852
113	935.4977
114	947.9634
115	949.7016
116	952.9613
117	956.5558
118	978.0244
119	995.4482
120	1021.587
121	1036.775
122	1040.288
123	1054.206
124	1061.812
125	1062.01
126	1065.865
127	1066.516
128	1075.32
129	1075.741
130	1076.129

131	1076.425
132	1077.954
133	1078.189
134	1084.368
135	1097.383
136	1146.221
137	1146.605
138	1165.515
139	1165.561
140	1168.959
141	1169.405
142	1169.843
143	1170.058
144	1207.385
145	1213.976
146	1219.471
147	1221.956
148	1222.326
149	1222.986
150	1226.09
151	1234.572
152	1247.228
153	1254.4
154	1255.629
155	1260.807
156	1263.833
157	1263.882
158	1264.579
159	1266.464
160	1275.586
161	1304.687
162	1310.638
163	1313.352
164	1315.361
165	1317.151
166	1318.458
167	1321.969
168	1322.348
169	1322.883
170	1323.777
171	1325.393

172	1328.376
173	1336.965
174	1346.404
175	1357.513
176	1364.218
177	1364.239
178	1365.251
179	1367.75
180	1374.332
181	1374.379
182	1375.594
183	1378.164
184	1379.297
185	1459.062
186	1482.066
187	1494.896
188	1498.218
189	1505.698
190	1505.712
191	1507.847
192	1507.861
193	1508.14
194	1508.588
195	1509.358
196	1509.872
197	1511.543
198	1511.875
199	1513.056
200	1514.008
201	1515.933
202	1515.949
203	1528.356
204	1528.433
205	1528.863
206	1529.307
207	1530.539
208	1531.372
209	1542.646
210	1560.9
211	1576.941
212	1588.131

213	1604.874
214	1605.261
215	3021.518
216	3021.56
217	3021.731
218	3021.89
219	3021.921
220	3021.96
221	3022.346
222	3022.442
223	3022.644
224	3022.726
225	3039.629
226	3039.647
227	3061.677
228	3061.735
229	3061.821
230	3061.94
231	3062.323
232	3062.463
233	3080.444
234	3080.568
235	3081.312
236	3081.563
237	3082.357
238	3082.405
239	3086.275
240	3086.442
241	3087.54
242	3087.614
243	3112.001
244	3112.024
245	3131.192
246	3131.235
247	3131.413
248	3131.429
249	3131.608
250	3131.629
251	3251.892
252	3251.893

Optimized coordinates of the molecules

Optimization of (5,5-bis(methoxymethyl)-5,6-dihydro-4H-cyclopenta[c]thiophene-1,3-diyl)bis(trimethylsilane)

Center Number	Atomic Number	Atomic Type	Coordinates (Angstroms) X	Y	Z
1	6	0	1.569598	-1.101887	-0.234474
2	6	0	0.272736	-0.661539	-0.390643
3	6	0	0.080117	0.766244	-0.268890
4	6	0	1.219239	1.500123	-0.014885
5	16	0	2.629837	0.347579	0.084065
6	14	0	1.441397	3.368064	0.237325
7	14	0	2.274307	-2.863656	-0.276026
8	6	0	-2.120867	-0.263100	-0.297017
9	6	0	-1.050992	-1.345431	-0.687022
10	1	0	-1.125563	-1.584936	-1.759487
11	1	0	-1.215120	-2.273102	-0.130554
12	6	0	-1.384837	1.114225	-0.473226
13	1	0	-1.766042	1.871693	0.221060
14	1	0	-1.559664	1.502491	-1.486712
15	6	0	0.612218	3.884535	1.879683
16	1	0	0.715768	4.965737	2.046638
17	1	0	-0.459438	3.645753	1.877338
18	1	0	1.071409	3.363972	2.729965
19	6	0	3.301514	3.780164	0.296035
20	1	0	3.801927	3.501584	-0.640219
21	1	0	3.451541	4.857295	0.450704

22	1	0	3.804403	3.250796	1.115729
23	6	0	0.610146	4.292866	-1.211894
24	1	0	-0.458644	4.052138	-1.278364
25	1	0	0.702266	5.380200	-1.083154
26	1	0	1.077019	4.023767	-2.168135
27	6	0	1.646127	-3.824978	1.250757
28	1	0	0.548956	-3.851866	1.279108
29	1	0	2.010075	-4.861803	1.238278
30	1	0	1.994707	-3.353643	2.178815
31	6	0	1.674932	-3.733739	-1.866956
32	1	0	2.053715	-4.764180	-1.912451
33	1	0	0.579024	-3.777037	-1.908759
34	1	0	2.026892	-3.203122	-2.761033
35	6	0	4.178704	-2.783174	-0.253732
36	1	0	4.565961	-2.244704	-1.128216
37	1	0	4.548482	-2.273207	0.645298
38	1	0	4.606041	-3.795061	-0.264638
39	6	0	-3.370078	-0.374755	-1.179975
40	6	0	-2.514008	-0.420968	1.187092
41	8	0	-4.270416	0.724655	-0.839525
42	8	0	-3.057624	-1.762923	1.383748
43	6	0	-5.533544	0.677851	-1.552753
44	6	0	-3.529322	-2.000630	2.735560
45	1	0	-5.382745	0.749108	-2.642683
46	1	0	-6.083831	-0.251054	-1.331107
47	1	0	-6.118355	1.537083	-1.212391
48	1	0	-2.712743	-1.899287	3.469188
49	1	0	-4.337470	-1.301649	3.005774
50	1	0	-3.911919	-3.025032	2.759372

51	1	0	-1.630254	-0.281640	1.831897
52	1	0	-3.275605	0.329698	1.445677
53	1	0	-3.873428	-1.338170	-1.003953
54	1	0	-3.090381	-0.303498	-2.245946

Optimization of Chalcogenophene

Furan

Center Number	Atomic Number	Atomic Type	Coordinates (Angstroms) X	Y	Z
1	6	0	0.000024	-0.347132	1.122908
2	6	0	0.000024	0.969736	0.725189
3	6	0	0.000024	0.969736	-0.725189
4	6	0	0.000024	-0.347132	-1.122908
5	8	0	0.000146	-1.180992	0.000000
6	1	0	-0.001202	-0.845620	-2.078736
7	1	0	-0.001202	-0.845620	2.078736
8	1	0	0.000328	1.833966	-1.373233
9	1	0	0.000328	1.833966	1.373233

Thiophene

Center Number	Atomic Number	Atomic Type	Coordinates (Angstroms) X	Y	Z

1	6	0	-0.000010	-0.030230	1.268445
2	6	0	-0.000010	-1.294088	0.723139
3	6	0	-0.000010	-1.294088	-0.723139
4	6	0	-0.000010	-0.030230	-1.268445
5	1	0	0.000068	0.262427	-2.308367
6	1	0	0.000068	0.262427	2.308367
7	16	0	-0.000001	1.235310	0.000000
8	1	0	0.000054	-2.198999	1.321411
9	1	0	0.000054	-2.198999	-1.321411

Selenophene

Center Number	Atomic Number	Atomic Type	Coordinates (Angstroms) X	Y	Z
1	6	0	0.000170	0.459410	1.307188
2	6	0	0.000170	1.705472	0.724068
3	6	0	0.000170	1.705472	-0.724068
4	6	0	0.000170	0.459410	-1.307188
5	1	0	0.000014	0.219176	-2.362519
6	1	0	0.000014	0.219176	2.362519
7	34	0	-0.000115	-0.931194	0.000000
8	1	0	-0.000090	2.621834	-1.307456
9	1	0	-0.000090	2.621834	1.307456

Tellurophene

Center Atomic Atomic Coordinates (Angstroms)

Number	Number	Type	X	Y	Z
1	6	0	0.000632	0.787992	1.360178
2	6	0	0.000632	2.009918	0.725547
3	6	0	0.000632	2.009918	-0.725547
4	6	0	0.000632	0.787992	-1.360178
5	1	0	0.000945	0.629940	-2.431925
6	1	0	0.000945	0.629940	2.431925
7	52	0	-0.000367	-0.783054	0.000000
8	1	0	0.001025	2.941995	1.286845
9	1	0	0.001025	2.941995	-1.286845

Optimization of CPX

CPO

Center Number	Atomic Number	Atomic Type	Coordinates (Angstroms) X	Y	Z
1	6	0	-0.109217	-0.723756	0.049014
2	6	0	-0.109218	0.723755	0.049015
3	6	0	2.159390	0.000000	-0.249924
4	6	0	1.301988	-1.259723	0.132689
5	1	0	1.533551	-1.590547	1.155494
6	1	0	1.495179	-2.106822	-0.535586
7	6	0	1.301987	1.259723	0.132690
8	1	0	1.495176	2.106823	-0.535585
9	1	0	1.533549	1.590547	1.155495

10	1	0	3.140280	0.000000	0.239681
11	1	0	2.328348	0.000000	-1.334601
12	8	0	-2.248285	0.000000	-0.063460
13	6	0	-1.413546	-1.135355	-0.018263
14	1	0	-1.923417	-2.084087	-0.049480
15	6	0	-1.413546	1.135354	-0.018264
16	1	0	-1.923415	2.084088	-0.049483

CPS

Center Number	Atomic Number	Atomic Type	Coordinates (Angstroms) X	Y	Z
1	6	0	-0.059164	-1.012928	1.280570
2	6	0	-0.050702	0.238762	0.724773
3	6	0	-0.050702	0.238762	-0.724773
4	6	0	-0.059164	-1.012928	-1.280570
5	16	0	-0.066491	-2.285372	0.000000
6	6	0	0.415896	2.485564	0.000000
7	6	0	-0.050702	1.664146	1.248795
8	1	0	-1.065829	1.962644	1.549654
9	1	0	0.603809	1.813044	2.115198
10	6	0	-0.050702	1.664146	-1.248795
11	1	0	0.603809	1.813044	-2.115198
12	1	0	-1.065829	1.962644	-1.549654
13	1	0	0.021940	3.508671	0.000000
14	1	0	1.511904	2.548939	0.000000
15	1	0	-0.057253	-1.318092	-2.316964

16	1	0	-0.057253	-1.318092	2.316964

CPSe

Center Number	Atomic Number	Atomic Type	Coordinates (Angstroms) X	Y	Z
1	6	0	-0.043983	-0.466809	1.319705
2	6	0	-0.036217	0.766780	0.726349
3	6	0	-0.036217	0.766780	-0.726349
4	6	0	-0.043983	-0.466809	-1.319705
5	6	0	0.441491	3.013684	0.000000
6	6	0	-0.036217	2.196652	1.245384
7	1	0	-1.053828	2.497831	1.535803
8	1	0	0.610264	2.344757	2.117922
9	6	0	-0.036217	2.196652	-1.245384
10	1	0	0.610264	2.344757	-2.117922
11	1	0	-1.053828	2.497831	-1.535803
12	1	0	0.059504	4.041397	0.000000
13	1	0	1.538275	3.063406	0.000000
14	34	0	-0.055257	-1.864473	0.000000
15	1	0	-0.041920	-0.719736	-2.372067
16	1	0	-0.041920	-0.719736	2.372067

CPTe

Center Atomic Atomic Coordinates (Angstroms)

Number	Number	Type	X	Y	Z
1	6	0	0.033597	0.040656	1.372004
2	6	0	0.033597	-1.168595	0.729215
3	6	0	0.033597	-1.168595	-0.729215
4	6	0	0.033597	0.040656	-1.372004
5	6	0	-0.448004	-3.418186	0.000000
6	6	0	0.039165	-2.603995	1.240930
7	1	0	1.060798	-2.903854	1.519025
8	1	0	-0.596643	-2.753071	2.121082
9	6	0	0.039165	-2.603995	-1.240930
10	1	0	-0.596643	-2.753071	-2.121082
11	1	0	1.060798	-2.903854	-1.519025
12	1	0	-0.075337	-4.449398	0.000000
13	1	0	-1.545225	-3.457391	0.000000
14	52	0	0.039333	1.617167	0.000000
15	1	0	0.029320	0.210138	-2.442433
16	1	0	0.029320	0.210138	2.442433

Optimization of 2CPX

2CPO

Center Number	Atomic Number	Atomic Type	Coordinates (Angstroms) X	Y	Z
1	6	0	2.848837	0.906486	-0.045631
2	6	0	1.939665	-0.212860	-0.062215

3	6	0	4.148381	-1.091093	0.245873
4	6	0	4.282684	0.432444	-0.117350
5	1	0	4.681933	0.555964	-1.134426
6	1	0	4.958613	0.960736	0.564862
7	6	0	2.687559	-1.520676	-0.140157
8	1	0	2.288019	-2.292329	0.526456
9	1	0	2.653425	-1.929269	-1.160623
10	1	0	4.908340	-1.704196	-0.252432
11	1	0	4.281025	-1.211770	1.328938
12	8	0	0.731000	1.700941	0.046397
13	6	0	2.099754	2.051864	0.018692
14	1	0	2.304362	3.108814	0.057723
15	6	0	0.658411	0.288033	-0.007707
16	6	0	-1.939658	0.212867	0.062113
17	6	0	-2.848814	-0.906489	0.045530
18	6	0	-4.148480	1.091106	-0.245249
19	6	0	-2.687561	1.520649	0.140476
20	1	0	-2.653208	1.929010	1.161030
21	1	0	-2.288216	2.292468	-0.526059
22	6	0	-4.282647	-0.432506	0.117737
23	1	0	-4.958758	-0.960693	-0.564384
24	1	0	-4.681612	-0.556207	1.134899
25	1	0	-4.908302	1.704099	0.253397
26	1	0	-4.281431	1.211985	-1.328250
27	8	0	-0.730982	-1.700903	-0.047000
28	6	0	-0.658405	-0.288000	0.007325
29	6	0	-2.099728	-2.051847	-0.019098
30	1	0	-2.304328	-3.108790	-0.058343

2CPS

Center Number	Atomic Number	Atomic Type	Coordinates (Angstroms) X	Y	Z
1	6	0	2.993584	0.745438	-0.060006
2	6	0	1.923483	-0.223784	-0.052582
3	6	0	3.995264	-1.413923	0.273173
4	6	0	4.340325	0.051989	-0.148157
5	1	0	4.717217	0.079818	-1.181152
6	1	0	5.107274	0.502883	0.491911
7	6	0	2.494289	-1.630453	-0.112952
8	1	0	1.995204	-2.335117	0.561540
9	1	0	2.404882	-2.041819	-1.129738
10	1	0	4.656806	-2.152110	-0.194891
11	1	0	4.103347	-1.511251	1.361261
12	6	0	2.576744	2.048244	-0.004272
13	1	0	3.148388	2.964962	0.007313
14	6	0	0.651214	0.315503	-0.003736
15	6	0	-1.923482	0.223784	0.052581
16	6	0	-2.993585	-0.745437	0.060006
17	6	0	-3.995260	1.413924	-0.273187
18	6	0	-2.494286	1.630454	0.112943
19	1	0	-2.404883	2.041823	1.129728
20	1	0	-1.995198	2.335114	-0.561550
21	6	0	-4.340325	-0.051986	0.148149
22	1	0	-5.107272	-0.502881	-0.491920
23	1	0	-4.717221	-0.079809	1.181142

24	1	0	-4.656803	2.152114	0.194870
25	1	0	-4.103338	1.511247	-1.361276
26	6	0	-0.651214	-0.315505	0.003740
27	6	0	-2.576746	-2.048243	0.004278
28	1	0	-3.148391	-2.964961	-0.007305
29	16	0	0.778890	2.146298	0.057774
30	16	0	-0.778893	-2.146300	-0.057762

2CPSe

Center Number	Atomic Number	Atomic Type	Coordinates (Angstroms) X	Y	Z
1	6	0	3.095482	0.303801	-0.073442
2	6	0	1.876215	-0.474686	-0.048863
3	6	0	3.732535	-1.986747	0.290188
4	6	0	4.307380	-0.608240	-0.163753
5	1	0	4.666385	-0.661421	-1.202324
6	1	0	5.148153	-0.277641	0.456665
7	6	0	2.216379	-1.958638	-0.088613
8	1	0	1.619235	-2.564177	0.603509
9	1	0	2.055953	-2.366705	-1.098685
10	1	0	4.260157	-2.833987	-0.163038
11	1	0	3.828444	-2.077305	1.380045
12	6	0	2.945388	1.663464	-0.030664
13	1	0	3.706287	2.433520	-0.037098
14	6	0	0.686517	0.232454	-0.006373
15	6	0	-1.876225	0.474690	0.048854

16	6	0	-3.095483	-0.303810	0.073441
17	6	0	-3.732566	1.986726	-0.290206
18	6	0	-2.216409	1.958639	0.088591
19	1	0	-2.055985	2.366717	1.098658
20	1	0	-1.619276	2.564179	-0.603539
21	6	0	-4.307393	0.608217	0.163748
22	1	0	-5.148163	0.277602	-0.456664
23	1	0	-4.666395	0.661402	1.202319
24	1	0	-4.260198	2.833964	0.163014
25	1	0	-3.828480	2.077273	-1.380064
26	6	0	-0.686519	-0.232439	0.006371
27	6	0	-2.945375	-1.663472	0.030672
28	1	0	-3.706265	-2.433536	0.037114
29	34	0	1.097224	2.148392	0.039763
30	34	0	-1.097206	-2.148382	-0.039751

2CPTe

Center Number	Atomic Number	Atomic Type	Coordinates (Angstroms) X	Y	Z
1	6	0	3.055608	-0.750725	-0.086324
2	6	0	1.632056	-1.039017	-0.044616
3	6	0	2.842495	-3.115149	0.309874
4	6	0	3.855456	-2.041385	-0.183883
5	1	0	4.140589	-2.230662	-1.229629
6	1	0	4.777007	-2.023340	0.408880
7	6	0	1.433227	-2.553141	-0.058592

8	1	0	0.678359	-2.901728	0.657970
9	1	0	1.124371	-2.897641	-1.058119
10	1	0	3.023679	-4.104033	-0.126931
11	1	0	2.916853	-3.211513	1.400828
12	6	0	3.465187	0.555440	-0.051529
13	1	0	4.488798	0.911161	-0.075848
14	6	0	0.726711	0.014179	-0.007933
15	6	0	-1.632072	1.039025	0.044631
16	6	0	-3.055621	0.750712	0.086332
17	6	0	-2.842546	3.115143	-0.309835
18	6	0	-1.433270	2.553153	0.058623
19	1	0	-1.124418	2.897646	1.058153
20	1	0	-0.678412	2.901764	-0.657937
21	6	0	-3.855490	2.041357	0.183909
22	1	0	-4.777041	2.023304	-0.408854
23	1	0	-4.140626	2.230616	1.229658
24	1	0	-3.023746	4.104019	0.126983
25	1	0	-2.916908	3.211520	-1.400787
26	6	0	-0.726713	-0.014159	0.007939
27	6	0	-3.465181	-0.555459	0.051520
28	1	0	-4.488787	-0.911195	0.075833
29	52	0	1.841546	1.842640	0.032563
30	52	0	-1.841523	-1.842635	-0.032580

Optimization of 3CPX

3CPO

Center Number	Atomic Number	Atomic Type	Coordinates (Angstroms) X	Y	Z
1	6	0	0.717300	2.224836	0.066709
2	6	0	-0.717312	2.224837	0.066772
3	6	0	-0.000030	4.493617	-0.227850
4	6	0	1.262319	3.630004	0.134055
5	1	0	1.620644	3.858622	1.148223
6	1	0	2.100919	3.805410	-0.548136
7	6	0	-1.262337	3.629994	0.134191
8	1	0	-2.101012	3.805386	-0.547919
9	1	0	-1.620572	3.858592	1.148391
10	1	0	-0.000003	5.462658	0.284603
11	1	0	-0.000082	4.689188	-1.308109
12	8	0	-0.000013	0.073390	-0.018579
13	6	0	1.142295	0.914011	0.016827
14	6	0	-1.142326	0.914022	0.016964
15	6	0	-2.851898	-1.044679	-0.058534
16	6	0	-4.293368	-1.031078	-0.059892
17	6	0	-3.588717	-3.303969	0.255661
18	6	0	-2.321439	-2.455658	-0.120844
19	1	0	-1.973099	-2.697371	-1.135762
20	1	0	-1.481220	-2.636545	0.558307
21	6	0	-4.842520	-2.437903	-0.129531
22	1	0	-5.689299	-2.616878	0.542988
23	1	0	-5.179185	-2.673084	-1.149533
24	1	0	-3.595282	-4.284004	-0.235560
25	1	0	-3.593986	-3.474841	1.340069
26	8	0	-3.557081	1.107773	0.026294

27	6	0	-2.420562	0.263237	-0.008174
28	6	0	-4.699408	0.276674	-0.009673
29	1	0	-5.644757	0.792636	0.014455
30	6	0	4.293366	-1.031071	-0.060095
31	6	0	2.851881	-1.044696	-0.058218
32	6	0	3.588882	-3.303795	0.256915
33	6	0	4.842504	-2.437917	-0.129284
34	1	0	5.178673	-2.673580	-1.149341
35	1	0	5.689603	-2.616583	0.542914
36	6	0	2.321439	-2.455725	-0.119561
37	1	0	1.481483	-2.636291	0.559999
38	1	0	1.972740	-2.698003	-1.134225
39	1	0	3.595257	-4.284105	-0.233752
40	1	0	3.594593	-3.474040	1.341421
41	8	0	3.557063	1.107826	0.025242
42	6	0	4.699396	0.276713	-0.010656
43	6	0	2.420552	0.263247	-0.008374
44	1	0	5.644737	0.792715	0.012883

3CPS

Center Number	Atomic Number	Atomic Type	Coordinates (Angstroms) X	Y	Z
1	6	0	0.718191	1.860059	0.037652
2	6	0	-0.718192	1.860059	0.037651
3	6	0	-0.000000	4.138118	-0.274377
4	6	0	1.249131	3.280693	0.107980

5	1	0	1.592452	3.513694	1.127116
6	1	0	2.094818	3.464974	-0.563768
7	6	0	-1.249132	3.280693	0.107979
8	1	0	-2.094818	3.464974	-0.563771
9	1	0	-1.592454	3.513693	1.127113
10	1	0	-0.000001	5.117932	0.216831
11	1	0	0.000001	4.309328	-1.358597
12	6	0	1.310199	0.610005	-0.004841
13	6	0	-1.310199	0.610005	-0.004843
14	6	0	-3.287906	-1.039279	-0.054447
15	6	0	-4.730925	-1.031476	-0.050702
16	6	0	-4.034476	-3.309796	0.265107
17	6	0	-2.775472	-2.467762	-0.127992
18	1	0	-2.444590	-2.708979	-1.149538
19	1	0	-1.927559	-2.664100	0.537657
20	6	0	-5.272662	-2.445877	-0.141958
21	1	0	-6.139619	-2.624703	0.504241
22	1	0	-5.580369	-2.671181	-1.173686
23	1	0	-4.038890	-4.298995	-0.207287
24	1	0	-4.040952	-3.458965	1.352680
25	6	0	-2.698924	0.212492	-0.003566
26	6	0	-5.290175	0.216661	0.015752
27	1	0	-6.327726	0.517202	0.036737
28	6	0	4.730925	-1.031475	-0.050702
29	6	0	3.287906	-1.039278	-0.054454
30	6	0	4.034475	-3.309800	0.265073
31	6	0	5.272663	-2.445875	-0.141973
32	1	0	5.580376	-2.671166	-1.173702
33	1	0	6.139617	-2.624709	0.504228

34	6	0	2.775473	-2.467760	-0.128019
35	1	0	1.927557	-2.664107	0.537623
36	1	0	2.444594	-2.708964	-1.149570
37	1	0	4.038891	-4.298992	-0.207334
38	1	0	4.040945	-3.458984	1.352644
39	6	0	5.290175	0.216662	0.015770
40	6	0	2.698924	0.212493	-0.003562
41	1	0	6.327726	0.517202	0.036763
42	16	0	-4.017134	1.489735	0.074839
43	16	0	-0.000000	-0.668377	-0.065677
44	16	0	4.017133	1.489734	0.074866

3CPSe

Center Number	Atomic Number	Atomic Type	Coordinates (Angstroms) X	Y	Z
1	6	0	0.719819	-1.612264	-0.000451
2	6	0	-0.719826	-1.612254	-0.000455
3	6	0	-0.000024	-3.892315	0.339034
4	6	0	1.244892	-3.039305	-0.054119
5	1	0	1.588941	-3.285918	-1.070471
6	1	0	2.085814	-3.221350	0.625381
7	6	0	-1.244922	-3.039286	-0.054131
8	1	0	-2.085856	-3.221316	0.625359
9	1	0	-1.588964	-3.285892	-1.070487
10	1	0	-0.000030	-4.878494	-0.139084
11	1	0	-0.000031	-4.048791	1.425467

12	6	0	1.355672	-0.380984	0.025070
13	6	0	-1.355664	-0.380969	0.025067
14	6	0	-3.395077	1.186920	0.051456
15	6	0	-4.840871	1.178341	0.043961
16	6	0	-4.152025	3.460165	-0.275647
17	6	0	-2.893192	2.623288	0.121070
18	1	0	-2.563052	2.869994	1.142292
19	1	0	-2.050249	2.826883	-0.549941
20	6	0	-5.382764	2.594581	0.139705
21	1	0	-6.255999	2.770605	-0.498703
22	1	0	-5.681947	2.817755	1.174614
23	1	0	-4.157364	4.452589	0.189858
24	1	0	-4.160469	3.601212	-1.364370
25	6	0	-2.763429	-0.046649	0.009988
26	6	0	-5.438499	-0.051336	-0.025852
27	1	0	-6.492923	-0.295942	-0.044934
28	6	0	4.840863	1.178353	0.043966
29	6	0	3.395069	1.186912	0.051469
30	6	0	4.151980	3.460171	-0.275614
31	6	0	5.382735	2.594601	0.139720
32	1	0	5.681922	2.817768	1.174630
33	1	0	6.255963	2.770644	-0.498692
34	6	0	2.893162	2.623271	0.121104
35	1	0	2.050209	2.826858	-0.549896
36	1	0	2.563027	2.869960	1.142332
37	1	0	4.157307	4.452590	0.189902
38	1	0	4.160413	3.601229	-1.364336
39	6	0	5.438507	-0.051314	-0.025862
40	6	0	2.763437	-0.046662	0.009990

41	1	0	6.492935	-0.295906	-0.044952
42	34	0	-4.133385	-1.446697	-0.082701
43	34	0	0.000012	1.018563	0.078634
44	34	0	4.133411	-1.446691	-0.082720

3CPTe

Center Number	Atomic Number	Atomic Type	Coordinates (Angstroms) X	Y	Z
1	6	0	0.723716	-1.372193	0.080260
2	6	0	-0.723727	-1.372184	0.080257
3	6	0	-0.000025	-3.644474	0.513516
4	6	0	1.237544	-2.807914	0.081298
5	1	0	1.577053	-3.100053	-0.924888
6	1	0	2.074145	-2.969292	0.772748
7	6	0	-1.237577	-2.807896	0.081287
8	1	0	-2.074189	-2.969262	0.772727
9	1	0	-1.577080	-3.100029	-0.924903
10	1	0	-0.000030	-4.650391	0.078821
11	1	0	-0.000030	-3.751986	1.605613
12	6	0	1.421976	-0.169234	0.062942
13	6	0	-1.421974	-0.169219	0.062940
14	6	0	-3.548380	1.274789	0.051876
15	6	0	-5.000053	1.259084	0.029338
16	6	0	-4.333021	3.550236	-0.282875
17	6	0	-3.071212	2.724375	0.120450
18	1	0	-2.744683	2.980361	1.140624

19	1	0	-2.236203	2.943582	-0.557413
20	6	0	-5.549077	2.674044	0.136206
21	1	0	-6.430418	2.843131	-0.492753
22	1	0	-5.840544	2.888091	1.175416
23	1	0	-4.345249	4.544422	0.178604
24	1	0	-4.340471	3.685862	-1.372216
25	6	0	-2.853349	0.069439	0.023943
26	6	0	-5.646475	0.054685	-0.059766
27	1	0	-6.718924	-0.100672	-0.084630
28	6	0	5.000040	1.259097	0.029340
29	6	0	3.548367	1.274782	0.051882
30	6	0	4.332974	3.550241	-0.282870
31	6	0	5.549044	2.674065	0.136206
32	1	0	5.840512	2.888115	1.175416
33	1	0	6.430381	2.843165	-0.492755
34	6	0	3.071177	2.724362	0.120460
35	1	0	2.236160	2.943555	-0.557397
36	1	0	2.744651	2.980340	1.140637
37	1	0	4.345189	4.544426	0.178609
38	1	0	4.340417	3.685867	-1.372211
39	6	0	5.646478	0.054706	-0.059768
40	6	0	2.853350	0.069426	0.023948
41	1	0	6.718928	-0.100636	-0.084635
42	52	0	-4.292467	-1.513278	-0.113651
43	52	0	0.000009	1.406361	0.085754
44	52	0	4.292489	-1.513273	-0.113654

Optimization of 4CPX

4CPO

Center Number	Atomic Number	Atomic Type	Coordinates (Angstroms) X	Y	Z
1	6	0	0.623935	-1.851141	0.063170
2	6	0	2.012999	-2.205552	0.065704
3	6	0	0.759246	-4.227293	-0.229805
4	6	0	-0.251118	-3.078469	0.130139
5	1	0	-0.656088	-3.211547	1.143962
6	1	0	-1.105456	-3.042644	-0.554473
7	6	0	2.194832	-3.701599	0.134293
8	1	0	2.965325	-4.078716	-0.546495
9	1	0	2.484089	-4.010991	1.149062
10	1	0	0.519187	-5.166195	0.282588
11	1	0	0.712869	-4.416974	-1.310120
12	8	0	1.851585	0.056385	-0.020823
13	6	0	0.535739	-0.474321	0.012952
14	6	0	2.750062	-1.040680	0.016627
15	6	0	4.891863	0.432645	-0.056079
16	6	0	6.284770	0.061875	-0.054420
17	6	0	6.165388	2.438763	0.259169
18	6	0	4.728072	1.931051	-0.119859
19	1	0	4.452734	2.250848	-1.135603
20	1	0	3.957547	2.315313	0.557270
21	6	0	7.165921	1.288450	-0.123152
22	1	0	8.029274	1.252192	0.551119
23	1	0	7.552443	1.431961	-1.142493

24	1	0	6.415782	3.386118	-0.232319
25	1	0	6.210682	2.603905	1.343525
26	8	0	5.040892	-1.827684	0.030921
27	6	0	4.149341	-0.727510	-0.006140
28	6	0	6.353694	-1.305738	-0.002955
29	1	0	7.141592	-2.039916	0.023282
30	6	0	-6.284770	-0.061868	0.054455
31	6	0	-4.891863	-0.432639	0.056185
32	6	0	-6.165385	-2.438804	-0.258769
33	6	0	-7.165923	-1.288432	0.123364
34	1	0	-7.552457	-1.431787	1.142722
35	1	0	-8.029267	-1.252277	-0.550923
36	6	0	-4.728074	-1.931035	0.120201
37	1	0	-3.957539	-2.315405	-0.556856
38	1	0	-4.452753	-2.250673	1.135999
39	1	0	-6.415787	-3.386084	0.232859
40	1	0	-6.210665	-2.604110	-1.343101
41	8	0	-5.040890	1.827678	-0.031150
42	6	0	-6.353693	1.305737	0.002791
43	1	0	-7.141590	2.039912	-0.023560
44	6	0	-4.149340	0.727508	0.006082
45	6	0	-2.013000	2.205550	-0.065789
46	6	0	-0.623935	1.851142	-0.063260
47	6	0	-0.759247	4.227297	0.229681
48	6	0	-2.194835	3.701597	-0.134396
49	1	0	-2.484102	4.010974	-1.149168
50	1	0	-2.965323	4.078723	0.546392
51	6	0	0.251115	3.078471	-0.130259
52	1	0	1.105463	3.042658	0.554341

53	1	0	0.656070	3.211534	-1.144090
54	1	0	-0.519194	5.166194	-0.282725
55	1	0	-0.712860	4.416991	1.309994
56	8	0	-1.851583	-0.056385	0.020753
57	6	0	-2.750060	1.040678	-0.016703
58	6	0	-0.535737	0.474323	-0.013025

4CPS

Center Number	Atomic Number	Atomic Type	Coordinates (Angstroms) X	Y	Z
1	6	0	-1.064935	1.618893	0.040205
2	6	0	-2.485209	1.825022	0.038448
3	6	0	-1.447927	3.976913	-0.269766
4	6	0	-0.335690	2.948674	0.113878
5	1	0	0.034929	3.128963	1.134197
6	1	0	0.528900	3.010771	-0.556241
7	6	0	-2.807574	3.306969	0.109508
8	1	0	-3.616777	3.610963	-0.563633
9	1	0	-3.116083	3.585733	1.128264
10	1	0	-1.308384	4.946284	0.222412
11	1	0	-1.421445	4.146982	-1.353842
12	6	0	-0.657433	0.295074	-0.001681
13	6	0	-3.251772	0.672992	-0.005029
14	6	0	-5.445980	-0.674116	-0.056853
15	6	0	-6.872649	-0.458623	-0.054536
16	6	0	-6.511980	-2.813689	0.260399

17	6	0	-5.144413	-2.161415	-0.130690
18	1	0	-4.850371	-2.447250	-1.151987
19	1	0	-4.334470	-2.478142	0.535839
20	6	0	-7.612428	-1.780192	-0.147104
21	1	0	-8.496620	-1.832596	0.498356
22	1	0	-7.948535	-1.958249	-1.179196
23	1	0	-6.658211	-3.791554	-0.212936
24	1	0	-6.541065	-2.961255	1.347808
25	6	0	-4.682704	0.479992	-0.005100
26	6	0	-7.246588	0.857045	0.011813
27	1	0	-8.230173	1.303619	0.031843
28	6	0	6.872649	0.458624	0.054534
29	6	0	5.445980	0.674116	0.056849
30	6	0	6.511980	2.813688	-0.260412
31	6	0	7.612428	1.780194	0.147096
32	1	0	7.948534	1.958255	1.179187
33	1	0	8.496619	1.832594	-0.498364
34	6	0	5.144412	2.161416	0.130679
35	1	0	4.334469	2.478140	-0.535852
36	1	0	4.850370	2.447256	1.151975
37	1	0	6.658210	3.791555	0.212918
38	1	0	6.541065	2.961248	-1.347822
39	6	0	7.246588	-0.857045	-0.011809
40	1	0	8.230173	-1.303619	-0.031837
41	6	0	4.682704	-0.479992	0.005101
42	6	0	2.485209	-1.825022	-0.038446
43	6	0	1.064935	-1.618893	-0.040203
44	6	0	1.447927	-3.976914	0.269768
45	6	0	2.807574	-3.306969	-0.109507

46	1	0	3.116084	-3.585733	-1.128262
47	1	0	3.616777	-3.610963	0.563635
48	6	0	0.335690	-2.948674	-0.113876
49	1	0	-0.528900	-3.010771	0.556243
50	1	0	-0.034929	-3.128963	-1.134195
51	1	0	1.308384	-4.946284	-0.222411
52	1	0	1.421445	-4.146983	1.353844
53	6	0	3.251772	-0.672992	0.005030
54	6	0	0.657433	-0.295074	0.001683
55	16	0	5.803725	-1.933773	-0.072395
56	16	0	2.139530	0.780899	0.064855
57	16	0	-2.139529	-0.780899	-0.064854
58	16	0	-5.803724	1.933773	0.072404

4CPSe

Center Number	Atomic Number	Atomic Type	Coordinates (Angstroms) X	Y	Z
1	6	0	-1.236584	1.490179	0.012555
2	6	0	-2.671123	1.593698	0.006138
3	6	0	-1.788691	3.817067	-0.323558
4	6	0	-0.610524	2.875827	0.073056
5	1	0	-0.254708	3.094344	1.091893
6	1	0	0.244155	2.999401	-0.602402
7	6	0	-3.093334	3.054452	0.061144
8	1	0	-3.915534	3.297732	-0.622047
9	1	0	-3.424126	3.322366	1.076500

10	1	0	-1.720435	4.799620	0.157169
11	1	0	-1.772214	3.975505	-1.409618
12	6	0	-0.689199	0.214704	-0.010764
13	6	0	-3.394983	0.411264	-0.023928
14	6	0	-5.541991	-1.004862	-0.061651
15	6	0	-6.983225	-0.891728	-0.060069
16	6	0	-6.462679	-3.217986	0.257434
17	6	0	-5.144998	-2.473618	-0.132402
18	1	0	-4.829451	-2.741546	-1.152934
19	1	0	-4.321805	-2.739118	0.541434
20	6	0	-7.625809	-2.264845	-0.160967
21	1	0	-8.512138	-2.378357	0.473548
22	1	0	-7.936046	-2.463830	-1.197555
23	1	0	-6.537842	-4.206542	-0.210212
24	1	0	-6.485800	-3.360061	1.345816
25	6	0	-4.822764	0.179974	-0.015168
26	6	0	-7.490782	0.377870	0.009706
27	1	0	-8.524867	0.697954	0.024857
28	6	0	6.983224	0.891731	0.060067
29	6	0	5.541990	1.004864	0.061647
30	6	0	6.462675	3.217987	-0.257446
31	6	0	7.625806	2.264849	0.160960
32	1	0	7.936041	2.463839	1.197548
33	1	0	8.512136	2.378360	-0.473555
34	6	0	5.144994	2.473619	0.132391
35	1	0	4.321802	2.739116	-0.541445
36	1	0	4.829447	2.741552	1.152923
37	1	0	6.537837	4.206545	0.210196
38	1	0	6.485797	3.360058	-1.345829

39	6	0	7.490782	-0.377866	-0.009703
40	1	0	8.524868	-0.697950	-0.024851
41	6	0	4.822764	-0.179973	0.015168
42	6	0	2.671125	-1.593699	-0.006138
43	6	0	1.236585	-1.490183	-0.012555
44	6	0	1.788696	-3.817070	0.323558
45	6	0	3.093337	-3.054453	-0.061145
46	1	0	3.424130	-3.322366	-1.076500
47	1	0	3.915538	-3.297732	0.622047
48	6	0	0.610528	-2.875831	-0.073055
49	1	0	-0.244151	-2.999407	0.602404
50	1	0	0.254711	-3.094349	-1.091892
51	1	0	1.720441	-4.799623	-0.157170
52	1	0	1.772220	-3.975508	1.409618
53	6	0	3.394983	-0.411265	0.023928
54	6	0	0.689199	-0.214708	0.010764
55	34	0	6.088599	-1.675163	-0.074559
56	34	0	2.144238	1.082324	0.072689
57	34	0	-2.144240	-1.082326	-0.072689
58	34	0	-6.088598	1.675165	0.074566

4CPTe

Center Number	Atomic Number	Atomic Type	Coordinates (Angstroms) X	Y	Z
1	6	0	-1.393517	1.343575	-0.046407
2	6	0	-2.839181	1.367961	-0.060824

3	6	0	-2.076032	3.632059	-0.467410
4	6	0	-0.856399	2.771179	-0.032895
5	1	0	-0.517123	3.049376	0.977167
6	1	0	-0.014190	2.925378	-0.719257
7	6	0	-3.330567	2.811457	-0.054963
8	1	0	-4.157380	2.991633	-0.753441
9	1	0	-3.675813	3.100125	0.950278
10	1	0	-2.063692	4.633494	-0.022654
11	1	0	-2.064568	3.750378	-1.558363
12	6	0	-0.712764	0.127466	-0.024794
13	6	0	-3.559373	0.176838	-0.058520
14	6	0	-5.710622	-1.229190	-0.083006
15	6	0	-7.161719	-1.188131	-0.074213
16	6	0	-6.538273	-3.494276	0.217197
17	6	0	-5.258220	-2.686074	-0.163746
18	1	0	-4.925512	-2.935839	-1.183499
19	1	0	-4.434366	-2.928022	0.519947
20	6	0	-7.734576	-2.591797	-0.202825
21	1	0	-8.624712	-2.752539	0.415840
22	1	0	-8.019861	-2.788453	-1.247175
23	1	0	-6.563383	-4.482465	-0.256444
24	1	0	-6.558851	-3.642826	1.304693
25	6	0	-4.994540	-0.036379	-0.035596
26	6	0	-7.787994	0.026453	0.021934
27	1	0	-8.857772	0.200295	0.037845
28	6	0	7.161539	1.188421	0.074168
29	6	0	5.710438	1.229245	0.083021
30	6	0	6.537688	3.494469	-0.217172
31	6	0	7.734166	2.592181	0.202775

32	1	0	8.019469	2.788867	1.247115
33	1	0	8.624245	2.753083	-0.415930
34	6	0	5.257788	2.686047	0.163823
35	1	0	4.433843	2.927861	-0.519811
36	1	0	4.925099	2.935727	1.183603
37	1	0	6.562656	4.482654	0.256484
38	1	0	6.558185	3.643041	-1.304668
39	6	0	7.788003	-0.026062	-0.022029
40	1	0	8.857807	-0.199734	-0.037985
41	6	0	4.994539	0.036331	0.035606
42	6	0	2.839210	-1.368144	0.060872
43	6	0	1.393544	-1.343765	0.046456
44	6	0	2.076049	-3.632248	0.467466
45	6	0	3.330588	-2.811645	0.055035
46	1	0	3.675855	-3.100326	-0.950196
47	1	0	4.157377	-2.991827	0.753537
48	6	0	0.856425	-2.771369	0.032939
49	1	0	0.014211	-2.925568	0.719294
50	1	0	0.517158	-3.049567	-0.977125
51	1	0	2.063714	-4.633682	0.022710
52	1	0	2.064573	-3.750565	1.558419
53	6	0	3.559389	-0.177005	0.058556
54	6	0	0.712780	-0.127661	0.024844
55	52	0	6.407831	-1.569585	-0.105694
56	52	0	2.165396	1.421548	0.071626
57	52	0	-2.165395	-1.421729	-0.071585
58	52	0	-6.407585	1.569763	0.105611

Optimization of 5CPX

5CPO

Center Number	Atomic Number	Atomic Type	Coordinates (Angstroms) X	Y	Z
1	6	0	-2.843532	1.992683	0.063177
2	6	0	-4.276883	1.991282	0.062802
3	6	0	-3.563466	4.260718	-0.232771
4	6	0	-2.300384	3.398737	0.130138
5	1	0	-1.942886	3.628782	1.144437
6	1	0	-1.462579	3.575544	-0.552980
7	6	0	-4.824456	3.395458	0.129180
8	1	0	-5.662956	3.569052	-0.553556
9	1	0	-5.183711	3.623958	1.143013
10	1	0	-3.564861	5.230047	0.279148
11	1	0	-3.563378	4.455484	-1.313163
12	8	0	-3.559348	-0.160273	-0.020016
13	6	0	-2.416238	0.680515	0.015118
14	6	0	-4.701903	0.679813	0.014233
15	6	0	-6.411926	-1.278202	-0.059928
16	6	0	-7.853241	-1.263795	-0.062205
17	6	0	-7.150100	-3.536743	0.256215
18	6	0	-5.882138	-2.689496	-0.120317
19	1	0	-5.533218	-2.932515	-1.134729
20	1	0	-5.042469	-2.870160	0.559559
21	6	0	-8.403188	-2.670383	-0.130595
22	1	0	-9.250449	-2.848102	0.541640

23	1	0	-8.739435	-2.906399	-1.150534
24	1	0	-7.156845	-4.517205	-0.234100
25	1	0	-7.156108	-3.706626	1.340766
26	8	0	-7.116133	0.875003	0.021957
27	6	0	-5.979815	0.029787	-0.010817
28	6	0	-8.258735	0.044261	-0.013761
29	1	0	-9.203928	0.560567	0.009191
30	6	0	4.276891	1.991264	0.062757
31	6	0	2.843538	1.992671	0.063139
32	6	0	3.563464	4.260701	-0.232831
33	6	0	4.824463	3.395441	0.129099
34	1	0	5.183737	3.623951	1.142927
35	1	0	5.662949	3.569024	-0.553651
36	6	0	2.300390	3.398725	0.130108
37	1	0	1.462576	3.575524	-0.553003
38	1	0	1.942904	3.628781	1.144406
39	1	0	3.564875	5.230034	0.279082
40	1	0	3.563341	4.455460	-1.313225
41	8	0	3.559334	-0.160284	-0.020078
42	6	0	4.701886	0.679784	0.014204
43	6	0	2.416220	0.680513	0.015094
44	6	0	0.716197	-1.284646	-0.055551
45	6	0	-0.716237	-1.284642	-0.055518
46	6	0	-0.000011	-3.553443	0.240467
47	6	0	1.261735	-2.689736	-0.122339
48	1	0	1.619911	-2.919307	-1.136485
49	1	0	2.099509	-2.865105	0.561186
50	6	0	-1.261778	-2.689733	-0.122258
51	1	0	-2.099510	-2.865098	0.561316

52	1	0	-1.620017	-2.919303	-1.136386
53	1	0	-0.000031	-4.523089	-0.270982
54	1	0	0.000032	-3.747406	1.321015
55	8	0	-0.000007	0.867783	0.026569
56	6	0	1.143310	0.027847	-0.007885
57	6	0	-1.143324	0.027862	-0.007834
58	6	0	7.853257	-1.263770	-0.062168
59	6	0	6.411929	-1.278208	-0.059860
60	6	0	7.150176	-3.536720	0.256314
61	6	0	8.403215	-2.670352	-0.130614
62	1	0	8.739339	-2.906350	-1.150600
63	1	0	9.250552	-2.848092	0.541517
64	6	0	5.882182	-2.689520	-0.120207
65	1	0	5.042530	-2.870211	0.559682
66	1	0	5.533258	-2.932569	-1.134613
67	1	0	7.156915	-4.517215	-0.233931
68	1	0	7.156253	-3.706517	1.340879
69	8	0	7.116106	0.875018	0.021916
70	6	0	8.258726	0.044292	-0.013777
71	6	0	5.979807	0.029775	-0.010775
72	1	0	9.203903	0.560628	0.009153

5CPS

Center Number	Atomic Number	Atomic Type	Coordinates (Angstroms) X	Y	Z
1	6	0	-3.291454	1.697153	0.040854

2	6	0	-4.726320	1.696358	0.040739
3	6	0	-4.010587	3.975810	-0.265493
4	6	0	-2.761257	3.118012	0.115161
5	1	0	-2.418587	3.348780	1.135073
6	1	0	-1.915820	3.304749	-0.556255
7	6	0	-5.259069	3.116235	0.113633
8	1	0	-6.104007	3.300946	-0.558911
9	1	0	-5.604115	3.346473	1.132777
10	1	0	-4.011646	4.954221	0.228534
11	1	0	-4.010122	4.150015	-1.349221
12	6	0	-2.697123	0.445598	-0.002749
13	6	0	-5.319086	0.445777	-0.003072
14	6	0	-7.296368	-1.203348	-0.054047
15	6	0	-8.739137	-1.195568	-0.050257
16	6	0	-8.042719	-3.474447	0.262074
17	6	0	-6.783708	-2.631613	-0.129344
18	1	0	-6.452188	-2.871405	-1.151017
19	1	0	-5.936124	-2.828764	0.536499
20	6	0	-9.281004	-2.609818	-0.143208
21	1	0	-10.147554	-2.789457	0.503312
22	1	0	-9.589427	-2.833622	-1.175029
23	1	0	-8.047093	-4.462660	-0.212346
24	1	0	-8.049154	-3.625889	1.349321
25	6	0	-6.707146	0.048706	-0.002021
26	6	0	-9.298621	0.052533	0.017413
27	1	0	-10.336225	0.352859	0.038683
28	6	0	4.726320	1.696358	0.040739
29	6	0	3.291454	1.697153	0.040854
30	6	0	4.010587	3.975810	-0.265493

31	6	0	5.259069	3.116235	0.113634
32	1	0	5.604115	3.346473	1.132778
33	1	0	6.104007	3.300946	-0.558911
34	6	0	2.761257	3.118012	0.115161
35	1	0	1.915820	3.304749	-0.556255
36	1	0	2.418587	3.348780	1.135073
37	1	0	4.011646	4.954221	0.228534
38	1	0	4.010122	4.150015	-1.349221
39	6	0	5.319086	0.445777	-0.003071
40	6	0	2.697123	0.445598	-0.002748
41	6	0	0.716903	-1.200115	-0.044844
42	6	0	-0.716903	-1.200115	-0.044844
43	6	0	0.000000	-3.479415	0.259910
44	6	0	1.248642	-2.620419	-0.119807
45	1	0	1.592068	-2.850142	-1.139681
46	1	0	2.093890	-2.806582	0.551986
47	6	0	-1.248642	-2.620419	-0.119807
48	1	0	-2.093890	-2.806582	0.551986
49	1	0	-1.592068	-2.850142	-1.139681
50	1	0	0.000000	-4.457807	-0.234265
51	1	0	-0.000000	-3.653587	1.343658
52	6	0	1.311550	0.051652	-0.001230
53	6	0	-1.311550	0.051652	-0.001231
54	6	0	8.739137	-1.195568	-0.050258
55	6	0	7.296368	-1.203348	-0.054048
56	6	0	8.042719	-3.474447	0.262071
57	6	0	9.281004	-2.609817	-0.143209
58	1	0	9.589427	-2.833620	-1.175031
59	1	0	10.147554	-2.789457	0.503311

60	6	0	6.783708	-2.631613	-0.129347
61	1	0	5.936123	-2.828764	0.536496
62	1	0	6.452188	-2.871404	-1.151021
63	1	0	8.047093	-4.462660	-0.212350
64	1	0	8.049154	-3.625890	1.349317
65	6	0	9.298620	0.052533	0.017415
66	6	0	6.707146	0.048706	-0.002021
67	1	0	10.336225	0.352859	0.038686
68	16	0	-8.025859	1.325819	0.077727
69	16	0	-4.009071	-0.832728	-0.065528
70	16	0	-0.000000	1.329990	0.061730
71	16	0	4.009071	-0.832728	-0.065527
72	16	0	8.025859	1.325819	0.077730

5CPSe

Center Number	Atomic Number	Atomic Type	Coordinates (Angstroms) X	Y	Z
1	6	0	3.401003	-1.526840	0.013587
2	6	0	4.838939	-1.526357	0.013217
3	6	0	4.121145	-3.808952	-0.311794
4	6	0	2.876314	-2.953849	0.076150
5	1	0	2.532164	-3.194176	1.094107
6	1	0	2.036145	-3.141083	-0.602824
7	6	0	5.365660	-2.952501	0.074641
8	1	0	6.205679	-3.137707	-0.605079
9	1	0	5.711521	-3.192447	1.091957

10	1	0	4.122095	-4.791898	0.172911
11	1	0	4.120696	-3.972588	-1.397207
12	6	0	2.762590	-0.294006	-0.015704
13	6	0	5.475821	-0.294667	-0.017517
14	6	0	7.514994	1.272737	-0.050911
15	6	0	8.960540	1.263935	-0.044025
16	6	0	8.272346	3.547385	0.265203
17	6	0	7.013213	2.708784	-0.126856
18	1	0	6.682459	2.950751	-1.149068
19	1	0	6.170772	2.915707	0.543733
20	6	0	9.502801	2.679571	-0.146305
21	1	0	10.376118	2.858288	0.491228
22	1	0	9.801982	2.897827	-1.182258
23	1	0	8.277651	4.537485	-0.205175
24	1	0	8.281185	3.693789	1.353224
25	6	0	6.882944	0.039084	-0.004023
26	6	0	9.558208	0.034424	0.030610
27	1	0	10.612646	-0.210108	0.050162
28	6	0	-4.838939	-1.526357	0.013217
29	6	0	-3.401003	-1.526840	0.013586
30	6	0	-4.121145	-3.808952	-0.311795
31	6	0	-5.365660	-2.952501	0.074640
32	1	0	-5.711521	-3.192447	1.091956
33	1	0	-6.205679	-3.137707	-0.605080
34	6	0	-2.876314	-2.953849	0.076149
35	1	0	-2.036145	-3.141083	-0.602826
36	1	0	-2.532165	-3.194177	1.094106
37	1	0	-4.122095	-4.791898	0.172909
38	1	0	-4.120696	-3.972587	-1.397209

39	6	0	-5.475821	-0.294667	-0.017517
40	6	0	-2.762590	-0.294007	-0.015704
41	6	0	-0.718371	1.267093	-0.029659
42	6	0	0.718371	1.267093	-0.029659
43	6	0	-0.000000	3.549832	0.293793
44	6	0	-1.244435	2.693619	-0.092617
45	1	0	-1.589789	2.932879	-1.110372
46	1	0	-2.084113	2.880367	0.587101
47	6	0	1.244435	2.693619	-0.092617
48	1	0	2.084112	2.880367	0.587101
49	1	0	1.589789	2.932879	-1.110372
50	1	0	-0.000000	4.532476	-0.191593
51	1	0	-0.000000	3.714085	1.379141
52	6	0	-1.357357	0.034089	-0.000892
53	6	0	1.357357	0.034089	-0.000892
54	6	0	-8.960540	1.263935	-0.044025
55	6	0	-7.514994	1.272737	-0.050911
56	6	0	-8.272346	3.547385	0.265202
57	6	0	-9.502801	2.679571	-0.146305
58	1	0	-9.801982	2.897826	-1.182259
59	1	0	-10.376118	2.858288	0.491228
60	6	0	-7.013213	2.708784	-0.126858
61	1	0	-6.170772	2.915708	0.543732
62	1	0	-6.682460	2.950750	-1.149069
63	1	0	-8.277651	4.537485	-0.205177
64	1	0	-8.281184	3.693789	1.353223
65	6	0	-9.558207	0.034424	0.030610
66	6	0	-6.882944	0.039084	-0.004023
67	1	0	-10.612646	-0.210108	0.050163

68	34	0	8.253180	-1.360575	0.093880
69	34	0	4.120739	1.104557	-0.075629
70	34	0	0.000000	-1.364503	0.057249
71	34	0	-4.120739	1.104557	-0.075629
72	34	0	-8.253180	-1.360575	0.093881

5CPTe

Center Number	Atomic Number	Atomic Type	Coordinates (Angstroms) X	Y	Z
1	6	0	-3.557439	-1.351944	0.045227
2	6	0	-5.002954	-1.351197	0.045473
3	6	0	-4.283574	-3.631889	0.439470
4	6	0	-3.044848	-2.788454	0.025261
5	1	0	-2.699099	-3.063869	-0.983395
6	1	0	-2.213138	-2.963144	0.719432
7	6	0	-5.519565	-2.785694	0.022817
8	1	0	-6.355745	-2.957062	0.712308
9	1	0	-5.860720	-3.059626	-0.987919
10	1	0	-4.284224	-4.629119	-0.014776
11	1	0	-4.284956	-3.760778	1.529286
12	6	0	-2.854951	-0.147466	0.039047
13	6	0	-5.702662	-0.147624	0.045472
14	6	0	-7.829250	1.295301	0.060616
15	6	0	-9.280599	1.279572	0.038130
16	6	0	-8.614577	3.576761	-0.229018
17	6	0	-7.352335	2.743360	0.157084

18	1	0	-7.024709	2.979296	1.181782
19	1	0	-6.518155	2.976219	-0.517177
20	6	0	-9.830273	2.691941	0.172559
21	1	0	-10.711567	2.872924	-0.453149
22	1	0	-10.122086	2.885350	1.215701
23	1	0	-8.626975	4.561414	0.252437
24	1	0	-8.622400	3.734364	-1.315402
25	6	0	-7.133451	0.090523	0.010586
26	6	0	-9.926970	0.076799	-0.073215
27	1	0	-10.999420	-0.078164	-0.100351
28	6	0	5.003088	-1.351273	0.045559
29	6	0	3.557570	-1.352221	0.045366
30	6	0	4.284107	-3.632050	0.439684
31	6	0	5.519931	-2.785683	0.022909
32	1	0	5.861051	-3.059594	-0.987845
33	1	0	6.356199	-2.956886	0.712337
34	6	0	3.045220	-2.788823	0.025529
35	1	0	2.213619	-2.963628	0.719798
36	1	0	2.699424	-3.064357	-0.983078
37	1	0	4.284880	-4.629299	-0.014521
38	1	0	4.285588	-3.760893	1.529505
39	6	0	5.702645	-0.147619	0.045490
40	6	0	2.854940	-0.147809	0.039132
41	6	0	0.722248	1.286393	-0.006192
42	6	0	-0.722007	1.286556	-0.006237
43	6	0	0.000440	3.566013	-0.408364
44	6	0	1.237380	2.722019	0.009054
45	1	0	1.582901	3.000034	1.017047
46	1	0	2.069862	2.892563	-0.685215

47	6	0	-1.236761	2.722323	0.008917
48	1	0	-2.069090	2.893091	-0.685475
49	1	0	-1.582341	3.000449	1.016858
50	1	0	0.000541	4.564399	0.043370
51	1	0	0.000518	3.692160	-1.498526
52	6	0	1.425382	0.081755	0.004747
53	6	0	-1.425382	0.082041	0.004684
54	6	0	9.280559	1.279674	0.038018
55	6	0	7.829209	1.295408	0.060503
56	6	0	8.614555	3.576847	-0.229294
57	6	0	9.830241	2.692049	0.172352
58	1	0	10.122049	2.885529	1.215482
59	1	0	10.711540	2.872984	-0.453363
60	6	0	7.352307	2.743480	0.156857
61	1	0	6.518144	2.976304	-0.517436
62	1	0	7.024668	2.979497	1.181532
63	1	0	8.626956	4.561533	0.252094
64	1	0	8.622387	3.734375	-1.315689
65	6	0	9.926931	0.076894	-0.073242
66	6	0	7.133417	0.090617	0.010565
67	1	0	10.999381	-0.078070	-0.100368
68	52	0	-8.572838	-1.489617	-0.156227
69	52	0	-4.281394	1.426336	0.083708
70	52	0	-0.000157	-1.491000	-0.026538
71	52	0	4.281190	1.426171	0.083677
72	52	0	8.572806	-1.489534	-0.156131

Optimization of 6CPX

6CPO

Center Number	Atomic Number	Atomic Type	Coordinates (Angstroms) X	Y	Z
1	6	0	-4.446081	2.072592	0.063252
2	6	0	-5.873025	2.207441	0.064049
3	6	0	-4.947409	4.398977	-0.231026
4	6	0	-3.771738	3.420638	0.130589
5	1	0	-3.393486	3.615103	1.144830
6	1	0	-2.921308	3.517516	-0.552954
7	6	0	-6.284670	3.657258	0.131461
8	1	0	-7.103315	3.910126	-0.550599
9	1	0	-6.619874	3.918411	1.145653
10	1	0	-4.856336	5.363785	0.281347
11	1	0	-4.929552	4.593395	-1.311338
12	8	0	-5.363381	-0.002477	-0.020084
13	6	0	-4.145447	0.725680	0.014277
14	6	0	-6.420874	0.942294	0.015398
15	6	0	-8.308725	-0.844882	-0.056278
16	6	0	-9.742175	-0.694052	-0.057468
17	6	0	-9.257213	-3.023159	0.262120
18	6	0	-7.915028	-2.300060	-0.116042
19	1	0	-7.591614	-2.575720	-1.130565
20	1	0	-7.095656	-2.559046	0.563261
21	6	0	-10.422911	-2.042280	-0.124456
22	1	0	-11.282703	-2.138511	0.548458
23	1	0	-10.780716	-2.246072	-1.144010

24	1	0	-9.357188	-3.998914	-0.227419
25	1	0	-9.278395	-3.190936	1.346810
26	8	0	-8.805791	1.365383	0.024966
27	6	0	-7.754652	0.416364	-0.008307
28	6	0	-10.021937	0.646563	-0.009506
29	1	0	-10.913989	1.250043	0.013840
30	6	0	2.640889	1.392736	0.058519
31	6	0	1.215272	1.529736	0.059331
32	6	0	2.144877	3.719960	-0.235034
33	6	0	3.318386	2.739154	0.126208
34	1	0	3.697308	2.932343	1.140405
35	1	0	4.168803	2.834253	-0.557562
36	6	0	0.806467	2.980449	0.127427
37	1	0	-0.010871	3.235744	-0.555615
38	1	0	0.472053	3.242392	1.141842
39	1	0	2.237735	4.684532	0.277521
40	1	0	2.162988	3.914340	-1.315347
41	8	0	1.722350	-0.681547	-0.024752
42	6	0	2.940653	0.045302	0.009774
43	6	0	0.664423	0.263853	0.011102
44	6	0	-1.215303	-1.529732	-0.059571
45	6	0	-2.640920	-1.392725	-0.058696
46	6	0	-2.144906	-3.719959	0.234777
47	6	0	-0.806511	-2.980446	-0.127738
48	1	0	-0.472158	-3.242360	-1.142180
49	1	0	0.010864	-3.235769	0.555249
50	6	0	-3.318428	-2.739136	-0.126376
51	1	0	-4.168806	-2.834244	0.557442
52	1	0	-3.697409	-2.932301	-1.140556

53	1	0	-2.237798	-4.684516	-0.277801
54	1	0	-2.162958	-3.914369	1.315084
55	8	0	-1.722366	0.681550	0.024613
56	6	0	-0.664445	-0.263857	-0.011283
57	6	0	-2.940674	-0.045291	-0.009881
58	6	0	9.742199	0.694010	0.057767
59	6	0	8.308745	0.844888	0.056389
60	6	0	9.257363	3.023072	-0.262286
61	6	0	10.422950	2.042229	0.124715
62	1	0	10.780454	2.246160	1.144352
63	1	0	11.282932	2.138382	-0.547963
64	6	0	7.915099	2.300094	0.115847
65	1	0	7.095814	2.558994	-0.563590
66	1	0	7.591606	2.575973	1.130289
67	1	0	9.357285	3.998937	0.227040
68	1	0	9.278726	3.190576	-1.347014
69	8	0	8.805754	-1.365410	-0.024404
70	6	0	10.021919	-0.646617	0.010028
71	6	0	7.754638	-0.416340	0.008506
72	6	0	5.873025	-2.207412	-0.064012
73	6	0	4.446077	-2.072574	-0.063282
74	6	0	4.947415	-4.398969	0.230915
75	6	0	6.284683	-3.657221	-0.131486
76	1	0	6.619932	-3.918320	-1.145678
77	1	0	7.103305	-3.910115	0.550592
78	6	0	3.771749	-3.420624	-0.130700
79	1	0	2.921293	-3.517541	0.552803
80	1	0	3.393541	-3.615046	-1.144966
81	1	0	4.856368	-5.363756	-0.281504

82	1	0	4.929521	-4.593435	1.311218
83	8	0	5.363358	0.002492	0.020294
84	6	0	6.420856	-0.942262	-0.015275
85	6	0	4.145430	-0.725664	-0.014301
86	1	0	10.913955	-1.250129	-0.013132

6CPS

Center Number	Atomic Number	Atomic Type	Coordinates (Angstroms) X	Y	Z
1	6	0	5.204796	1.747285	-0.040306
2	6	0	6.637282	1.828095	-0.039533
3	6	0	5.792911	4.063159	0.266147
4	6	0	4.594617	3.135649	-0.115075
5	1	0	4.239947	3.346441	-1.135221
6	1	0	3.739548	3.273998	0.555866
7	6	0	7.088442	3.275987	-0.112345
8	1	0	7.921184	3.508559	0.560555
9	1	0	7.420279	3.525407	-1.131363
10	1	0	5.738559	5.040044	-0.227921
11	1	0	5.782015	4.237052	1.349872
12	6	0	4.682489	0.463885	0.003107
13	6	0	7.300255	0.613216	0.004560
14	6	0	9.368248	-0.920552	0.056088
15	6	0	10.808218	-0.830550	0.052648
16	6	0	10.242917	-3.145423	-0.259824
17	6	0	8.937820	-2.375731	0.131310

18	1	0	8.620336	-2.634016	1.152935
19	1	0	8.102991	-2.620931	-0.534688
20	6	0	11.429791	-2.211616	0.145767
21	1	0	12.305348	-2.341561	-0.500513
22	1	0	11.750184	-2.417470	1.177676
23	1	0	10.303499	-4.131781	0.214602
24	1	0	10.258242	-3.296245	-1.347064
25	6	0	8.708617	0.295897	0.003892
26	6	0	11.295686	0.447407	-0.014927
27	1	0	12.314511	0.806334	-0.035952
28	6	0	-2.798906	1.291587	-0.044480
29	6	0	-1.367747	1.373176	-0.043693
30	6	0	-2.213236	3.608175	0.259493
31	6	0	-3.410716	2.679255	-0.120337
32	1	0	-3.766232	2.888495	-1.140474
33	1	0	-4.265491	2.817218	0.551027
34	6	0	-0.917512	2.821367	-0.118897
35	1	0	-0.084776	3.055656	0.553450
36	1	0	-0.586878	3.069859	-1.138601
37	1	0	-2.268569	4.584612	-0.235410
38	1	0	-2.223802	3.782852	1.343098
39	6	0	-3.321682	0.007931	-0.000909
40	6	0	-0.702522	0.156976	0.000496
41	6	0	1.367747	-1.373175	0.043689
42	6	0	2.798906	-1.291587	0.044477
43	6	0	2.213236	-3.608174	-0.259501
44	6	0	0.917512	-2.821367	0.118891
45	1	0	0.586878	-3.069860	1.138594
46	1	0	0.084776	-3.055654	-0.553457

47	6	0	3.410716	-2.679255	0.120332
48	1	0	4.265491	-2.817216	-0.551032
49	1	0	3.766231	-2.888497	1.140468
50	1	0	2.268569	-4.584612	0.235400
51	1	0	2.223802	-3.782848	-1.343107
52	6	0	0.702522	-0.156975	-0.000498
53	6	0	3.321682	-0.007930	0.000907
54	6	0	-10.808219	0.830548	-0.052643
55	6	0	-9.368249	0.920551	-0.056088
56	6	0	-10.242918	3.145424	0.259812
57	6	0	-11.429792	2.211613	-0.145769
58	1	0	-11.750188	2.417461	-1.177679
59	1	0	-12.305348	2.341562	0.500512
60	6	0	-8.937821	2.375729	-0.131320
61	1	0	-8.102991	2.620934	0.534674
62	1	0	-8.620340	2.634008	-1.152947
63	1	0	-10.303501	4.131779	-0.214619
64	1	0	-10.258240	3.296252	1.347052
65	6	0	-11.295686	-0.447408	0.014940
66	6	0	-8.708617	-0.295897	-0.003886
67	6	0	-6.637281	-1.828095	0.039534
68	6	0	-5.204796	-1.747284	0.040305
69	6	0	-5.792911	-4.063158	-0.266152
70	6	0	-7.088441	-3.275987	0.112344
71	1	0	-7.420276	-3.525410	1.131361
72	1	0	-7.921185	-3.508558	-0.560556
73	6	0	-4.594616	-3.135649	0.115070
74	1	0	-3.739548	-3.273996	-0.555873
75	1	0	-4.239945	-3.346443	1.135215

76	1	0	-5.738558	-5.040044	0.227913
77	1	0	-5.782016	-4.237048	-1.349878
78	6	0	-7.300255	-0.613215	-0.004557
79	6	0	-4.682489	-0.463884	-0.003107
80	1	0	-12.314510	-0.806336	0.035970
81	16	0	9.952445	1.646105	-0.075566
82	16	0	6.065066	-0.737761	0.066591
83	16	0	1.939638	1.193912	-0.063132
84	16	0	-1.939638	-1.193911	0.063132
85	16	0	-6.065066	0.737761	-0.066586
86	16	0	-9.952444	-1.646105	0.075583

6CPSe

Center Number	Atomic Number	Atomic Type	Coordinates (Angstroms) X	Y	Z
1	6	0	5.418822	1.552663	-0.012633
2	6	0	6.856098	1.593252	-0.010032
3	6	0	6.072866	3.854503	0.312864
4	6	0	4.853638	2.964033	-0.076772
5	1	0	4.504561	3.193977	-1.095473
6	1	0	4.007260	3.127436	0.600642
7	6	0	7.341935	3.033850	-0.071356
8	1	0	8.175330	3.243319	0.609469
9	1	0	7.682247	3.283168	-1.088295
10	1	0	6.046500	4.836956	-0.172131
11	1	0	6.066026	4.018310	1.398234

12	6	0	4.815756	0.302027	0.016329
13	6	0	7.527916	0.380231	0.022279
14	6	0	9.610997	-1.128160	0.059452
15	6	0	11.055701	-1.078041	0.054399
16	6	0	10.433479	-3.380385	-0.254441
17	6	0	9.150420	-2.577939	0.135663
18	1	0	8.825611	-2.828699	1.157671
19	1	0	8.315007	-2.809327	-0.535714
20	6	0	11.638089	-2.477537	0.158150
21	1	0	12.516977	-2.631528	-0.478179
22	1	0	11.942070	-2.686630	1.194595
23	1	0	10.466508	-4.369703	0.216446
24	1	0	10.447855	-3.526990	-1.342375
25	6	0	8.943985	0.086918	0.010958
26	6	0	11.618074	0.168011	-0.020290
27	1	0	12.665119	0.442566	-0.038679
28	6	0	-2.816891	1.316759	-0.028074
29	6	0	-1.381092	1.357967	-0.025265
30	6	0	-2.165129	3.619651	0.293540
31	6	0	-3.383721	2.727459	-0.093781
32	1	0	-3.734134	2.955284	-1.112428
33	1	0	-4.229561	2.890798	0.584316
34	6	0	-0.895897	2.798866	-0.088997
35	1	0	-0.063478	3.010603	0.592291
36	1	0	-0.555251	3.046568	-1.106353
37	1	0	-2.192338	4.601076	-0.193511
38	1	0	-2.171963	3.785637	1.378605
39	6	0	-3.420629	0.065876	0.000698
40	6	0	-0.706703	0.143483	0.005962

41	6	0	1.381092	-1.357965	0.025256
42	6	0	2.816891	-1.316757	0.028066
43	6	0	2.165129	-3.619649	-0.293554
44	6	0	0.895896	-2.798864	0.088985
45	1	0	0.555251	-3.046568	1.106341
46	1	0	0.063477	-3.010600	-0.592303
47	6	0	3.383721	-2.727458	0.093771
48	1	0	4.229561	-2.890796	-0.584326
49	1	0	3.734132	-2.955285	1.112417
50	1	0	2.192337	-4.601075	0.193495
51	1	0	2.171963	-3.785632	-1.378619
52	6	0	0.706703	-0.143481	-0.005970
53	6	0	3.420629	-0.065875	-0.000705
54	6	0	-11.055702	1.078038	-0.054387
55	6	0	-9.610998	1.128158	-0.059447
56	6	0	-10.433480	3.380384	0.254440
57	6	0	-11.638091	2.477533	-0.158141
58	1	0	-11.942077	2.686622	-1.194586
59	1	0	-12.516977	2.631526	0.478191
60	6	0	-9.150423	2.577937	-0.135666
61	1	0	-8.315008	2.809329	0.535707
62	1	0	-8.825618	2.828693	-1.157677
63	1	0	-10.466513	4.369700	-0.216451
64	1	0	-10.447851	3.526994	1.342373
65	6	0	-11.618074	-0.168014	0.020309
66	6	0	-8.943985	-0.086919	-0.010952
67	6	0	-6.856097	-1.593252	0.010029
68	6	0	-5.418821	-1.552663	0.012626
69	6	0	-6.072865	-3.854502	-0.312877

70	6	0	-7.341934	-3.033850	0.071349
71	1	0	-7.682241	-3.283172	1.088289
72	1	0	-8.175331	-3.243318	-0.609473
73	6	0	-4.853637	-2.964032	0.076760
74	1	0	-4.007260	-3.127433	-0.600656
75	1	0	-4.504558	-3.193980	1.095459
76	1	0	-6.046498	-4.836956	0.172114
77	1	0	-6.066027	-4.018304	-1.398248
78	6	0	-7.527916	-0.380232	-0.022278
79	6	0	-4.815756	-0.302026	-0.016334
80	1	0	-12.665119	-0.442570	0.038703
81	34	0	10.273777	1.525134	-0.086064
82	34	0	6.213308	-1.057131	0.079092
83	34	0	2.023935	1.293342	-0.062810
84	34	0	-2.023935	-1.293340	0.062804
85	34	0	-6.213309	1.057131	-0.079090
86	34	0	-10.273776	-1.525136	0.086082

6CPTe

Center Number	Atomic Number	Atomic Type	Coordinates (Angstroms) X	Y	Z
1	6	0	-5.688340	-1.359313	0.042726
2	6	0	-7.133716	-1.368471	0.048634
3	6	0	-6.397217	-3.645754	0.430414
4	6	0	-5.165975	-2.792157	0.014767
5	1	0	-4.822478	-3.061057	-0.996436

6	1	0	-4.330277	-2.963965	0.704844
7	6	0	-7.640619	-2.806360	0.022148
8	1	0	-8.472835	-2.986272	0.714245
9	1	0	-7.983931	-3.078474	-0.988348
10	1	0	-6.392861	-4.641108	-0.027902
11	1	0	-6.393355	-3.779047	1.519698
12	6	0	-4.994030	-0.149911	0.038685
13	6	0	-7.841721	-0.169692	0.056173
14	6	0	-9.978047	1.258593	0.084884
15	6	0	-11.429307	1.232993	0.067766
16	6	0	-10.780058	3.535684	-0.193102
17	6	0	-9.510704	2.709512	0.185125
18	1	0	-9.180875	2.943796	1.209492
19	1	0	-8.680670	2.950644	-0.491348
20	6	0	-11.988144	2.641038	0.209604
21	1	0	-12.872973	2.818394	-0.412131
22	1	0	-12.277350	2.828457	1.254567
23	1	0	-10.797398	4.518407	0.292114
24	1	0	-10.793002	3.697299	-1.278850
25	6	0	-9.274177	0.058771	0.027651
26	6	0	-12.067857	0.026255	-0.045666
27	1	0	-13.139318	-0.135939	-0.069364
28	6	0	2.871270	-1.298998	0.008781
29	6	0	1.427497	-1.309067	0.015473
30	6	0	2.167168	-3.586225	0.399691
31	6	0	3.396396	-2.730844	-0.017253
32	1	0	3.740076	-3.000061	-1.028241
33	1	0	4.232678	-2.899780	0.672827
34	6	0	0.922210	-2.748104	-0.005501

35	1	0	0.095238	-2.928814	0.692724
36	1	0	0.572350	-3.022222	-1.013088
37	1	0	2.171926	-4.581244	-0.059345
38	1	0	2.173261	-3.720368	1.488885
39	6	0	3.566469	-0.089402	0.000802
40	6	0	0.715291	-0.109047	0.013474
41	6	0	-1.427698	1.309607	-0.015623
42	6	0	-2.871472	1.299299	-0.008869
43	6	0	-2.167823	3.586639	-0.399848
44	6	0	-0.922687	2.748746	0.005265
45	1	0	-0.572787	3.022964	1.012812
46	1	0	-0.095828	2.929611	-0.693050
47	6	0	-3.396863	2.731045	0.017191
48	1	0	-4.233229	2.899817	-0.672827
49	1	0	-3.740519	3.000208	1.028202
50	1	0	-2.172730	4.581663	0.059177
51	1	0	-2.174020	3.720767	-1.489043
52	6	0	-0.715313	0.109677	-0.013612
53	6	0	-3.566482	0.089599	-0.000848
54	6	0	11.429304	-1.233316	-0.067723
55	6	0	9.978042	-1.258898	-0.084844
56	6	0	10.780037	-3.536006	0.193089
57	6	0	11.988128	-2.641362	-0.209598
58	1	0	12.277330	-2.828758	-1.254566
59	1	0	12.872957	-2.818741	0.412130
60	6	0	9.510690	-2.709814	-0.185112
61	1	0	8.680663	-2.950958	0.491364
62	1	0	9.180851	-2.944075	-1.209482
63	1	0	10.797367	-4.518717	-0.292151

64	1	0	10.792984	-3.697648	1.278834
65	6	0	12.067870	-0.026589	0.045738
66	6	0	9.274191	-0.059063	-0.027589
67	6	0	7.133842	1.368307	-0.048553
68	6	0	5.688466	1.359275	-0.042673
69	6	0	6.397553	3.645665	-0.430270
70	6	0	7.640873	2.806149	-0.022001
71	1	0	7.984182	3.078195	0.988514
72	1	0	8.473129	2.986007	-0.714067
73	6	0	5.166225	2.792162	-0.014675
74	1	0	4.330551	2.964061	-0.704758
75	1	0	4.822734	3.061056	0.996531
76	1	0	6.393274	4.641005	0.028077
77	1	0	6.393726	3.778992	-1.519550
78	6	0	7.841746	0.169473	-0.056122
79	6	0	4.994049	0.149937	-0.038686
80	1	0	13.139333	0.135590	0.069442
81	52	0	-10.703399	-1.530499	-0.139676
82	52	0	-6.431174	1.413794	0.095396
83	52	0	-2.130265	-1.472991	-0.046171
84	52	0	2.130485	1.473404	0.046112
85	52	0	6.431060	-1.413889	-0.095425
86	52	0	10.703435	1.530183	0.139775

Optimization of PCPXs

PCPO

Center Atomic Atomic Coordinates (Angstroms)

Number Number Type X Y Z

1	6	0	2.528481	1.588473	-0.003457
2	6	0	1.096825	1.617805	-0.006846
3	6	0	1.859879	3.856877	-0.396716
4	6	0	3.102885	2.983610	0.005190
5	1	0	3.464265	3.248395	1.009577
6	1	0	3.945196	3.112587	-0.683065
7	6	0	0.580199	3.035283	-0.000684
8	1	0	-0.252886	3.198714	-0.692797
9	1	0	0.225504	3.314672	1.002109
10	1	0	1.879063	4.846950	0.073447
11	1	0	1.865411	4.005026	-1.484457
12	8	0	1.768562	-0.551102	0.003128
13	6	0	2.928964	0.266285	0.005170
14	6	0	0.642687	0.312882	-0.000615
15	6	0	-1.096820	-1.617802	0.007090
16	6	0	-2.528476	-1.588470	0.003683
17	6	0	-1.859879	-3.856919	0.396691
18	6	0	-0.580193	-3.035279	0.000775
19	1	0	-0.225479	-3.314553	-1.002044
20	1	0	0.252878	-3.198788	0.692885
21	6	0	-3.102880	-2.983606	-0.005130
22	1	0	-3.945198	-3.112661	0.683102
23	1	0	-3.464250	-3.248277	-1.009551
24	1	0	-1.879056	-4.846937	-0.073587
25	1	0	-1.865426	-4.005194	1.484415
26	8	0	-1.768558	0.551106	-0.002669

27	6	0	-0.642683	-0.312878	0.000989
28	6	0	-2.928960	-0.266281	-0.004814
29	-2	0	7.116408	-0.145820	0.016879

PCPS

Center Number	Atomic Number	Atomic Type	Coordinates (Angstroms) X	Y	Z
1	6	0	2.712482	1.405793	-0.006001
2	6	0	1.294711	1.432413	-0.014993
3	6	0	2.048500	3.670920	-0.409299
4	6	0	3.273198	2.808293	0.002892
5	1	0	3.630774	3.074976	1.007123
6	1	0	4.118328	2.946898	-0.678203
7	6	0	0.787121	2.855051	-0.012838
8	1	0	-0.043492	3.025284	-0.704536
9	1	0	0.427149	3.135281	0.986845
10	1	0	2.064361	4.666831	0.041976
11	1	0	2.057898	3.805576	-1.496114
12	6	0	3.263810	0.137668	0.013138
13	6	0	0.695905	0.185925	-0.003135
14	6	0	-1.294711	-1.432413	0.014989
15	6	0	-2.712482	-1.405793	0.005997

16	6	0	-2.048500	-3.670919	0.409302
17	6	0	-0.787121	-2.855051	0.012838
18	1	0	-0.427150	-3.135284	-0.986844
19	1	0	0.043493	-3.025282	0.704536
20	6	0	-3.273198	-2.808294	-0.002892
21	1	0	-4.118328	-2.946897	0.678203
22	1	0	-3.630774	-3.074979	-1.007122
23	1	0	-2.064361	-4.666832	-0.041969
24	1	0	-2.057897	-3.805571	1.496117
25	6	0	-0.695906	-0.185925	0.003128
26	6	0	-3.263811	-0.137668	-0.013145
27	16	0	1.957034	-1.054688	0.000029
28	16	0	-1.957034	1.054687	-0.000040
29	-2	0	7.904395	-0.148487	0.050196

PCPSe

Center Number	Atomic Number	Atomic Type	Coordinates (Angstroms) X	Y	Z
1	6	0	2.795693	1.360791	-0.008175
2	6	0	1.360229	1.379364	-0.020598
3	6	0	2.110985	3.639227	-0.421695
4	6	0	3.340703	2.781520	0.006064
5	1	0	3.681715	3.053732	1.017032
6	1	0	4.187797	2.931753	-0.673464
7	6	0	0.852235	2.813801	-0.015346
8	1	0	0.021096	2.986106	-0.709146

9	1	0	0.501248	3.094836	0.989789
10	1	0	2.120114	4.638710	0.027798
11	1	0	2.122058	3.763956	-1.512247
12	6	0	3.419521	0.118808	0.015294
13	6	0	0.704150	0.153855	-0.007809
14	6	0	-1.360226	-1.379364	0.020595
15	6	0	-2.795691	-1.360789	0.008171
16	6	0	-2.110986	-3.639226	0.421696
17	6	0	-0.852235	-2.813802	0.015346
18	1	0	-0.501248	-3.094839	-0.989788
19	1	0	-0.021096	-2.986107	0.709147
20	6	0	-3.340703	-2.781518	-0.006066
21	1	0	-4.187798	-2.931748	0.673460
22	1	0	-3.681714	-3.053730	-1.017034
23	1	0	-2.120116	-4.638709	-0.027796
24	1	0	-2.122060	-3.763953	1.512248
25	6	0	-0.704147	-0.153856	0.007804
26	6	0	-3.419517	-0.118806	-0.015300
27	34	0	2.043766	-1.263303	-0.001254
28	34	0	-2.043761	1.263304	0.001246
29	-2	0	8.238407	-0.106458	0.070031

PCPTe

Center Number	Atomic Number	Atomic Type	Coordinates (Angstroms) X	Y	Z
1	6	0	2.845701	1.352797	-0.008195

2	6	0	1.402959	1.335889	-0.021191
3	6	0	2.101617	3.623345	-0.420705
4	6	0	3.344562	2.794190	0.007866
5	1	0	3.679601	3.077563	1.017958
6	1	0	4.180345	2.972620	-0.680410
7	6	0	0.870782	2.765351	-0.014184
8	1	0	0.043513	2.924728	-0.717285
9	1	0	0.511369	3.040880	0.989680
10	1	0	2.086057	4.621708	0.030753
11	1	0	2.109925	3.749462	-1.510828
12	6	0	3.564115	0.155945	0.014325
13	6	0	0.712740	0.122505	-0.011445
14	6	0	-1.403130	-1.335782	0.021137
15	6	0	-2.845871	-1.352906	0.008243
16	6	0	-2.101341	-3.623340	0.420710
17	6	0	-0.870697	-2.765144	0.014056
18	1	0	-0.511358	-3.040600	-0.989855
19	1	0	-0.043300	-2.924362	0.717046
20	6	0	-3.344473	-2.794394	-0.007737
21	1	0	-4.180128	-2.972978	0.680649
22	1	0	-3.679581	-3.077845	-1.017785
23	1	0	-2.085658	-4.621702	-0.030744
24	1	0	-2.109512	-3.749452	1.510835
25	6	0	-0.713068	-0.122323	0.011364
26	6	0	-3.564440	-0.156133	-0.014272
27	52	0	-2.157447	1.433734	0.023426
28	52	0	2.156911	-1.433740	-0.023406
29	-2	0	8.559000	0.101232	0.077392

Optimization of CHX

CHO

Center Number	Atomic Number	Atomic Type	Coordinates (Angstroms) X	Y	Z
1	6	0	-1.702328	1.122621	0.042580
2	6	0	-0.386461	0.727584	0.028734
3	6	0	-0.386463	-0.727574	-0.028948
4	6	0	-1.702326	-1.122625	-0.042567
5	6	0	2.104040	0.694181	-0.342293
6	1	0	2.085537	0.556169	-1.433998
7	1	0	3.030181	1.232962	-0.100137
8	6	0	2.103890	-0.694248	0.342756
9	1	0	2.084883	-0.556323	1.434459
10	1	0	3.030140	-1.233005	0.100961
11	6	0	0.883863	1.545205	0.098032
12	1	0	0.805262	2.448221	-0.522145
13	1	0	1.047213	1.887924	1.131466
14	6	0	0.883877	-1.545159	-0.098231
15	1	0	0.805096	-2.448481	0.521469
16	1	0	1.047576	-1.887354	-1.131789
17	8	0	-2.539939	-0.000000	-0.000108
18	1	0	-2.202454	-2.076691	-0.080911
19	1	0	-2.202471	2.076671	0.081116

CHS

Center Number	Atomic Number	Atomic Type	Coordinates (Angstroms) X	Y	Z
1	6	0	1.319381	1.262148	-0.033676
2	6	0	0.051852	0.728098	-0.023665
3	6	0	0.051853	-0.728102	0.023593
4	6	0	1.319382	-1.262150	0.033611
5	6	0	-2.461200	0.692364	0.343289
6	1	0	-2.444330	0.554769	1.435025
7	1	0	-3.383915	1.237002	0.100444
8	6	0	-2.461164	-0.692378	-0.343439
9	1	0	-2.444193	-0.554798	-1.435176
10	1	0	-3.383893	-1.237024	-0.100669
11	6	0	-1.238206	1.530220	-0.102407
12	1	0	-1.158943	2.444379	0.500884
13	1	0	-1.397375	1.854469	-1.142692
14	6	0	-1.238204	-1.530209	0.102396
15	1	0	-1.158917	-2.444433	-0.500791
16	1	0	-1.397427	-1.854343	1.142712
17	1	0	1.613388	-2.301762	0.068329
18	1	0	1.613394	2.301761	-0.068299
19	16	0	2.592503	0.000002	0.000126

CHSe

Center Atomic Atomic Coordinates (Angstroms)

Number	Number	Type	X	Y	Z
1	6	0	-0.010347	1.299506	0.761903
2	6	0	-0.000000	0.729852	-0.489468
3	6	0	-0.000000	-0.729852	-0.489468
4	6	0	0.010347	-1.299506	0.761903
5	6	0	-0.362224	0.682153	-3.010139
6	1	0	-1.449815	0.514483	-2.993687
7	1	0	-0.134146	1.234966	-3.931864
8	6	0	0.362224	-0.682153	-3.010139
9	1	0	1.449815	-0.514483	-2.993687
10	1	0	0.134146	-1.234966	-3.931864
11	6	0	0.061772	1.528192	-1.786183
12	1	0	-0.560420	2.429731	-1.708706
13	1	0	1.095598	1.873852	-1.943782
14	6	0	-0.061772	-1.528192	-1.786183
15	1	0	0.560420	-2.429731	-1.708706
16	1	0	-1.095598	-1.873852	-1.943782
17	1	0	0.008520	-2.355065	1.004418
18	1	0	-0.008520	2.355065	1.004418
19	34	0	-0.000000	0.000000	2.159820

CHTe

Center Number	Atomic Number	Atomic Type	Coordinates (Angstroms) X	Y	Z
1	6	0	-0.000000	1.349168	0.318926

2	6	0	0.007616	0.732472	-0.911311
3	6	0	-0.007616	-0.732472	-0.911311
4	6	0	-0.000000	-1.349168	0.318926
5	6	0	-0.351004	0.687358	-3.443007
6	1	0	-1.441266	0.537940	-3.427307
7	1	0	-0.113254	1.239097	-4.363037
8	6	0	0.351004	-0.687358	-3.443007
9	1	0	1.441266	-0.537940	-3.427307
10	1	0	0.113254	-1.239097	-4.363037
11	6	0	0.089202	1.520704	-2.217612
12	1	0	-0.508762	2.438462	-2.141508
13	1	0	1.132462	1.838589	-2.372521
14	6	0	-0.089202	-1.520704	-2.217612
15	1	0	0.508762	-2.438462	-2.141508
16	1	0	-1.132462	-1.838589	-2.372521
17	1	0	-0.013957	-2.421222	0.481344
18	1	0	0.013957	2.421222	0.481344
19	52	0	0.000000	0.000000	1.897733

Optimization of 2CHX

2CHO

Center Number	Atomic Number	Atomic Type	Coordinates (Angstroms) X	Y	Z
1	6	0	0.004904	0.719861	-0.009337
2	6	0	1.004620	1.677358	0.014407

3	6	0	0.348754	2.971717	-0.011848
4	6	0	-1.004620	2.737882	-0.049877
5	6	0	3.197875	2.852635	0.405818
6	1	0	3.090661	3.031334	1.486767
7	1	0	4.273711	2.775488	0.197990
8	6	0	2.593328	4.051885	-0.363648
9	1	0	2.667742	3.861504	-1.444999
10	1	0	3.168537	4.964130	-0.154662
11	6	0	2.506765	1.517545	0.026681
12	1	0	2.805416	0.716242	0.712900
13	1	0	2.849811	1.201767	-0.970668
14	6	0	1.109445	4.277614	0.026494
15	1	0	0.646871	5.020309	-0.637308
16	1	0	1.072000	4.698221	1.043253
17	8	0	-1.251757	1.356542	-0.048371
18	1	0	-1.878494	3.368078	-0.078441
19	6	0	-0.004904	-0.719861	-0.009337
20	6	0	-1.004620	-1.677358	0.014407
21	8	0	1.251757	-1.356542	-0.048371
22	6	0	-0.348754	-2.971717	-0.011848
23	6	0	-2.506765	-1.517545	0.026681
24	6	0	1.004620	-2.737882	-0.049877
25	6	0	-1.109445	-4.277614	0.026494
26	6	0	-3.197875	-2.852635	0.405818
27	1	0	-2.805416	-0.716242	0.712900
28	1	0	-2.849811	-1.201767	-0.970668
29	1	0	1.878494	-3.368078	-0.078441
30	6	0	-2.593328	-4.051885	-0.363648
31	1	0	-0.646871	-5.020309	-0.637308

32	1	0	-1.072000	-4.698221	1.043253
33	1	0	-3.090661	-3.031334	1.486767
34	1	0	-4.273711	-2.775488	0.197990
35	1	0	-2.667742	-3.861504	-1.444999
36	1	0	-3.168537	-4.964130	-0.154662

2CHS

Center Number	Atomic Number	Atomic Type	Coordinates (Angstroms) X	Y	Z
1	6	0	-0.336298	0.646734	-0.365590
2	6	0	0.115742	1.884562	0.066429
3	6	0	-0.837369	2.961584	-0.142523
4	6	0	-1.999669	2.544785	-0.747769
5	6	0	1.508477	3.480686	1.472110
6	1	0	0.926725	3.392884	2.402369
7	1	0	2.544416	3.706265	1.759693
8	6	0	0.927718	4.626800	0.615023
9	1	0	1.487655	4.691063	-0.330113
10	1	0	1.049598	5.589901	1.129326
11	6	0	1.475662	2.141777	0.697761
12	1	0	1.754458	1.306606	1.352804
13	1	0	2.241454	2.178169	-0.092030
14	6	0	-0.572592	4.384340	0.326336
15	1	0	-0.942307	5.104715	-0.415616
16	1	0	-1.140363	4.569135	1.251607
17	1	0	-2.870475	3.132837	-1.002979

18	6	0	0.336298	-0.646734	-0.365590
19	6	0	-0.115742	-1.884562	0.066429
20	6	0	0.837369	-2.961584	-0.142523
21	6	0	-1.475662	-2.141777	0.697761
22	6	0	1.999669	-2.544785	-0.747769
23	6	0	0.572592	-4.384340	0.326336
24	6	0	-1.508477	-3.480686	1.472110
25	1	0	-1.754458	-1.306606	1.352804
26	1	0	-2.241454	-2.178169	-0.092030
27	1	0	2.870475	-3.132837	-1.002979
28	6	0	-0.927718	-4.626800	0.615023
29	1	0	0.942307	-5.104715	-0.415616
30	1	0	1.140363	-4.569135	1.251607
31	1	0	-0.926725	-3.392884	2.402369
32	1	0	-2.544416	-3.706265	1.759693
33	1	0	-1.487655	-4.691063	-0.330113
34	1	0	-1.049598	-5.589901	1.129326
35	16	0	1.999669	-0.793578	-1.099107
36	16	0	-1.999669	0.793578	-1.099107

2CHSe

Center Number	Atomic Number	Atomic Type	Coordinates (Angstroms) X	Y	Z
1	6	0	-0.235798	0.691716	-0.194969
2	6	0	0.353128	1.813464	0.365594
3	6	0	-0.353128	3.067991	0.144212

4	6	0	-1.493587	2.960100	-0.616617
5	6	0	1.855267	3.025269	2.031165
6	1	0	1.167531	2.992466	2.889975
7	1	0	2.876701	3.027783	2.435603
8	6	0	1.594455	4.312649	1.219900
9	1	0	2.259178	4.329033	0.342913
10	1	0	1.831032	5.200744	1.821890
11	6	0	1.660689	1.771777	1.147071
12	1	0	1.709696	0.856400	1.750123
13	1	0	2.501710	1.714203	0.438088
14	6	0	0.117268	4.377144	0.767452
15	1	0	-0.033384	5.206820	0.063938
16	1	0	-0.506313	4.596875	1.648396
17	1	0	-2.172338	3.758272	-0.892305
18	6	0	0.235798	-0.691716	-0.194969
19	6	0	-0.353128	-1.813464	0.365594
20	6	0	0.353128	-3.067991	0.144212
21	6	0	-1.660689	-1.771777	1.147071
22	6	0	1.493587	-2.960100	-0.616617
23	6	0	-0.117268	-4.377144	0.767452
24	6	0	-1.855267	-3.025269	2.031165
25	1	0	-1.709696	-0.856400	1.750123
26	1	0	-2.501710	-1.714203	0.438088
27	1	0	2.172338	-3.758272	-0.892305
28	6	0	-1.594455	-4.312649	1.219900
29	1	0	0.033384	-5.206820	0.063938
30	1	0	0.506313	-4.596875	1.648396
31	1	0	-1.167531	-2.992466	2.889975
32	1	0	-2.876701	-3.027783	2.435603

33	1	0	-2.259178	-4.329033	0.342913
34	1	0	-1.831032	-5.200744	1.821890
35	34	0	-1.845605	1.174099	-1.167102
36	34	0	1.845605	-1.174099	-1.167102

2CHTe

Center Number	Atomic Number	Atomic Type	Coordinates (Angstroms) X	Y	Z
1	6	0	-0.018584	0.733052	-0.034620
2	6	0	0.754657	1.591127	0.730552
3	6	0	0.549234	3.029041	0.552398
4	6	0	-0.381014	3.414913	-0.384893
5	6	0	2.285412	2.118077	2.717747
6	1	0	1.502433	2.271467	3.476240
7	1	0	3.174323	1.743644	3.243839
8	6	0	2.591179	3.463775	2.028519
9	1	0	3.353041	3.309955	1.249362
10	1	0	3.005812	4.180776	2.750553
11	6	0	1.825579	1.064898	1.684670
12	1	0	1.465990	0.159262	2.188284
13	1	0	2.699744	0.751216	1.091468
14	6	0	1.303187	4.047432	1.406074
15	1	0	1.532933	4.939033	0.807931
16	1	0	0.644991	4.380248	2.224461
17	1	0	-0.635538	4.442825	-0.619630
18	6	0	0.018584	-0.733052	-0.034620

19	6	0	-0.754657	-1.591127	0.730552
20	6	0	-0.549234	-3.029041	0.552398
21	6	0	-1.825579	-1.064898	1.684670
22	6	0	0.381014	-3.414913	-0.384893
23	6	0	-1.303187	-4.047432	1.406074
24	6	0	-2.285412	-2.118077	2.717747
25	1	0	-1.465990	-0.159262	2.188284
26	1	0	-2.699744	-0.751216	1.091468
27	1	0	0.635538	-4.442825	-0.619630
28	6	0	-2.591179	-3.463775	2.028519
29	1	0	-1.532933	-4.939033	0.807931
30	1	0	-0.644991	-4.380248	2.224461
31	1	0	-1.502433	-2.271467	3.476240
32	1	0	-3.174323	-1.743644	3.243839
33	1	0	-3.353041	-3.309955	1.249362
34	1	0	-3.005812	-4.180776	2.750553
35	52	0	1.303187	-1.812415	-1.319523
36	52	0	-1.303187	1.812415	-1.319523

Optimization of 3CHX

3CHO

Center Number	Atomic Number	Atomic Type	Coordinates (Angstroms) X	Y	Z
1	6	0	1.132967	1.003345	-0.010745
2	6	0	0.722092	2.327175	-0.005320

3	6	0	-0.722090	2.327177	0.005329
4	6	0	-1.132970	1.003349	0.010764
5	6	0	0.678537	4.815543	0.371416
6	1	0	0.493488	4.800051	1.456439
7	1	0	1.230936	5.739317	0.151854
8	6	0	-0.678524	4.815532	-0.371458
9	1	0	-0.493482	4.799997	-1.456481
10	1	0	-1.230919	5.739315	-0.151925
11	6	0	1.544560	3.593879	-0.027351
12	1	0	2.415109	3.499573	0.632259
13	1	0	1.949774	3.750249	-1.038915
14	6	0	-1.544547	3.593887	0.027362
15	1	0	-2.415120	3.499578	-0.632211
16	1	0	-1.949715	3.750283	1.038945
17	8	0	-0.000002	0.162800	0.000004
18	6	0	2.416985	0.357419	-0.027296
19	6	0	2.842090	-0.960103	0.040538
20	8	0	3.539480	1.203167	-0.139661
21	6	0	4.290644	-0.940480	-0.033008
22	6	0	2.043490	-2.238720	0.137367
23	6	0	4.672071	0.375153	-0.140874
24	6	0	5.133857	-2.193291	0.037278
25	6	0	2.953070	-3.423980	0.550833
26	1	0	1.211705	-2.122953	0.842137
27	1	0	1.585348	-2.459918	-0.839330
28	1	0	5.620258	0.881255	-0.220776
29	6	0	4.270535	-3.446352	-0.261220
30	1	0	5.980144	-2.135738	-0.660334
31	1	0	5.564222	-2.293368	1.045656

32	1	0	3.194916	-3.349124	1.622157
33	1	0	2.407327	-4.366803	0.409346
34	1	0	4.031883	-3.485374	-1.334867
35	1	0	4.846046	-4.351388	-0.023907
36	6	0	-4.672077	0.375161	0.140833
37	6	0	-4.290647	-0.940475	0.032997
38	8	0	-3.539481	1.203168	0.139717
39	6	0	-2.842091	-0.960099	-0.040518
40	6	0	-5.133860	-2.193284	-0.037318
41	6	0	-2.416989	0.357424	0.027306
42	6	0	-2.043491	-2.238717	-0.137339
43	6	0	-4.270548	-3.446347	0.261200
44	1	0	-5.980168	-2.135733	0.660269
45	1	0	-5.564195	-2.293356	-1.045709
46	6	0	-2.953066	-3.423976	-0.550824
47	1	0	-1.211696	-2.122948	-0.842097
48	1	0	-1.585364	-2.459919	0.839364
49	1	0	-4.031920	-3.485370	1.334852
50	1	0	-4.846055	-4.351382	0.023874
51	1	0	-3.194889	-3.349119	-1.622153
52	1	0	-2.407327	-4.366800	-0.409327
53	1	0	-5.620266	0.881265	0.220696

3CHS

Center Number	Atomic Number	Atomic Type	Coordinates (Angstroms) X	Y	Z

1	6	0	-1.276817	0.510677	0.185942
2	6	0	-0.716215	1.777084	0.110912
3	6	0	0.716215	1.777083	-0.110907
4	6	0	1.276816	0.510677	-0.185936
5	6	0	-0.722474	4.293200	-0.270996
6	1	0	-0.697087	4.274355	-1.371141
7	1	0	-1.244702	5.213429	0.024268
8	6	0	0.722476	4.293200	0.270995
9	1	0	0.697089	4.274357	1.371141
10	1	0	1.244704	5.213427	-0.024270
11	6	0	-1.502516	3.070763	0.264364
12	1	0	-2.476581	2.981458	-0.233326
13	1	0	-1.720072	3.238073	1.330174
14	6	0	1.502516	3.070761	-0.264363
15	1	0	2.476582	2.981457	0.233327
16	1	0	1.720072	3.238069	-1.330173
17	6	0	-2.654731	0.112716	0.446702
18	6	0	-3.457378	-0.798763	-0.223486
19	6	0	-4.773704	-0.966721	0.368819
20	6	0	-3.041246	-1.578111	-1.461940
21	6	0	-4.961078	-0.200789	1.495450
22	6	0	-5.837206	-1.860001	-0.251855
23	6	0	-4.262350	-2.140970	-2.226951
24	1	0	-2.427146	-0.948942	-2.118542
25	1	0	-2.400330	-2.421156	-1.161914
26	1	0	-5.843892	-0.125841	2.115389
27	6	0	-5.231477	-2.861033	-1.263793
28	1	0	-6.390983	-2.393766	0.532101
29	1	0	-6.570814	-1.230940	-0.779795

30	1	0	-4.797010	-1.325033	-2.736712
31	1	0	-3.912985	-2.833077	-3.005135
32	1	0	-4.687300	-3.654307	-0.729400
33	1	0	-6.040835	-3.346432	-1.826126
34	6	0	4.961076	-0.200781	-1.495448
35	6	0	4.773703	-0.966719	-0.368822
36	6	0	3.457378	-0.798765	0.223485
37	6	0	5.837207	-1.860001	0.251847
38	6	0	2.654729	0.112716	-0.446698
39	6	0	3.041248	-1.578119	1.461937
40	6	0	5.231480	-2.861038	1.263782
41	1	0	6.390983	-2.393763	-0.532112
42	1	0	6.570815	-1.230942	0.779789
43	6	0	4.262354	-2.140979	2.226944
44	1	0	2.427149	-0.948954	2.118542
45	1	0	2.400333	-2.421163	1.161908
46	1	0	4.687303	-3.654310	0.729386
47	1	0	6.040839	-3.346438	1.826112
48	1	0	4.797013	-1.325045	2.736707
49	1	0	3.912991	-2.833090	3.005126
50	1	0	5.843890	-0.125828	-2.115388
51	16	0	3.528168	0.785128	-1.900705
52	16	0	-0.000001	-0.767253	0.000002
53	16	0	-3.528170	0.785117	1.900712

3CHSe

Center Atomic Atomic Coordinates (Angstroms)

Number	Number	Type	X	Y	Z
1	6	0	1.323228	-0.344775	0.117371
2	6	0	0.723245	-1.593082	0.074227
3	6	0	-0.723245	-1.593081	-0.074233
4	6	0	-1.323228	-0.344774	-0.117372
5	6	0	0.710456	-4.117291	-0.300542
6	1	0	0.639575	-4.099574	-1.398850
7	1	0	1.245646	-5.036626	-0.026221
8	6	0	-0.710458	-4.117292	0.300523
9	1	0	-0.639577	-4.099580	1.398832
10	1	0	-1.245647	-5.036626	0.026199
11	6	0	1.508966	-2.893266	0.201083
12	1	0	2.465447	-2.805554	-0.329509
13	1	0	1.763684	-3.057529	1.260021
14	6	0	-1.508967	-2.893264	-0.201096
15	1	0	-2.465447	-2.805554	0.329496
16	1	0	-1.763685	-3.057522	-1.260034
17	6	0	2.732958	-0.009700	0.311667
18	6	0	3.596845	0.652087	-0.545918
19	6	0	4.938322	0.883288	-0.027415
20	6	0	3.193580	1.136169	-1.933531
21	6	0	5.145009	0.434588	1.255879
22	6	0	6.031312	1.519734	-0.878204
23	6	0	4.417785	1.416288	-2.835330
24	1	0	2.519215	0.410093	-2.404729
25	1	0	2.615536	2.068679	-1.835108
26	1	0	6.063174	0.492942	1.828055
27	6	0	5.456723	2.284194	-2.093269

28	1	0	6.650333	2.185871	-0.262512
29	1	0	6.699584	0.727460	-1.250903
30	1	0	4.887895	0.468570	-3.139401
31	1	0	4.083418	1.919606	-3.752736
32	1	0	4.977523	3.217340	-1.760250
33	1	0	6.276291	2.564599	-2.769075
34	6	0	-5.145008	0.434595	-1.255878
35	6	0	-4.938322	0.883287	0.027418
36	6	0	-3.596845	0.652084	0.545922
37	6	0	-6.031313	1.519730	0.878210
38	6	0	-2.732958	-0.009698	-0.311667
39	6	0	-3.193580	1.136158	1.933537
40	6	0	-5.456724	2.284183	2.093279
41	1	0	-6.650333	2.185869	0.262522
42	1	0	-6.699584	0.727453	1.250905
43	6	0	-4.417786	1.416274	2.835337
44	1	0	-2.519216	0.410080	2.404731
45	1	0	-2.615537	2.068669	1.835119
46	1	0	-4.977524	3.217331	1.760265
47	1	0	-6.276292	2.564584	2.769087
48	1	0	-4.887897	0.468554	3.139403
49	1	0	-4.083419	1.919587	3.752746
50	1	0	-6.063173	0.492951	-1.828054
51	34	0	-3.603822	-0.390314	-2.004968
52	34	0	0.000000	1.059160	0.000002
53	34	0	3.603823	-0.390325	2.004966

3CHTe

Center Number	Atomic Number	Atomic Type	Coordinates (Angstroms) X	Y	Z
1	6	0	1.377567	-0.070060	-0.230940
2	6	0	0.729782	-0.044584	-1.454948
3	6	0	-0.729781	0.044595	-1.454948
4	6	0	-1.377568	0.070061	-0.230940
5	6	0	0.700661	0.321049	-3.991295
6	1	0	0.597161	1.416922	-3.977598
7	1	0	1.246481	0.059820	-4.908345
8	6	0	-0.700660	-0.321012	-3.991298
9	1	0	-0.597160	-1.416885	-3.977612
10	1	0	-1.246479	-0.059775	-4.908346
11	6	0	1.509776	-0.152852	-2.764330
12	1	0	2.452713	0.401141	-2.682283
13	1	0	1.793471	-1.206606	-2.918941
14	6	0	-1.509775	0.152878	-2.764329
15	1	0	-2.452712	-0.401114	-2.682289
16	1	0	-1.793468	1.206634	-2.918931
17	6	0	2.822280	-0.165102	0.004657
18	6	0	3.714873	0.880644	0.175132
19	6	0	5.118001	0.541496	0.415741
20	6	0	3.249998	2.335898	0.171359
21	6	0	5.434325	-0.795701	0.482062
22	6	0	6.184371	1.628708	0.538613
23	6	0	4.407408	3.339965	-0.029131
24	1	0	2.475585	2.479769	-0.592121
25	1	0	2.763463	2.550832	1.136592

26	1	0	6.430016	-1.190346	0.653406
27	6	0	5.589007	3.006547	0.904766
28	1	0	6.941834	1.327461	1.274152
29	1	0	6.705939	1.724075	-0.427116
30	1	0	4.754905	3.313665	-1.073391
31	1	0	4.039917	4.357480	0.162405
32	1	0	5.239769	3.003620	1.948423
33	1	0	6.370448	3.775008	0.826409
34	6	0	-5.434325	0.795696	0.482065
35	6	0	-5.118001	-0.541500	0.415740
36	6	0	-3.714873	-0.880646	0.175124
37	6	0	-6.184370	-1.628713	0.538607
38	6	0	-2.822280	0.165101	0.004657
39	6	0	-3.249998	-2.335900	0.171343
40	6	0	-5.589005	-3.006554	0.904751
41	1	0	-6.941832	-1.327470	1.274149
42	1	0	-6.705939	-1.724074	-0.427121
43	6	0	-4.407407	-3.339966	-0.029150
44	1	0	-2.475585	-2.479767	-0.592138
45	1	0	-2.763461	-2.550838	1.136575
46	1	0	-5.239765	-3.003633	1.948407
47	1	0	-6.370446	-3.775015	0.826391
48	1	0	-4.754906	-3.313661	-1.073409
49	1	0	-4.039916	-4.357482	0.162381
50	1	0	-6.430017	1.190341	0.653412
51	52	0	-3.796998	2.031138	0.189782
52	52	0	-0.000000	-0.000006	1.360774
53	52	0	3.796998	-2.031140	0.189772

Optimization of 4CHX

4CHO

Center Number	Atomic Number	Atomic Type	Coordinates (Angstroms) X	Y	Z
1	6	0	2.671135	1.188744	-0.087883
2	6	0	1.882621	2.324430	-0.192847
3	6	0	0.508194	1.887983	-0.245544
4	6	0	0.511671	0.502589	-0.165366
5	6	0	1.072869	4.701147	-0.013672
6	1	0	0.826273	4.698055	1.059198
7	1	0	1.337641	5.733292	-0.280479
8	6	0	-0.167644	4.243265	-0.816840
9	1	0	0.088492	4.213989	-1.886976
10	1	0	-0.983962	4.969192	-0.700264
11	6	0	2.289533	3.776841	-0.271986
12	1	0	3.099235	3.989471	0.436094
13	1	0	2.701430	3.988063	-1.270668
14	6	0	-0.654446	2.845436	-0.358586
15	1	0	-1.412366	2.450540	-1.045631
16	1	0	-1.151002	2.938436	0.619680
17	8	0	1.843888	0.046650	-0.067691
18	6	0	4.087387	0.961878	-0.003195
19	6	0	4.879332	-0.156485	0.205542
20	8	0	4.913002	2.093876	-0.162285
21	6	0	6.258526	0.291371	0.174010

22	6	0	4.490255	-1.601893	0.408766
23	6	0	6.238223	1.647270	-0.048591
24	6	0	7.429550	-0.639835	0.391308
25	6	0	5.685152	-2.418444	0.964268
26	1	0	3.623343	-1.678562	1.075779
27	1	0	4.172485	-2.035929	-0.552050
28	1	0	6.996785	2.406519	-0.147339
29	6	0	6.993599	-2.115065	0.195262
30	1	0	8.258272	-0.391832	-0.285383
31	1	0	7.813539	-0.518399	1.415927
32	1	0	5.833200	-2.181020	2.028936
33	1	0	5.451958	-3.490146	0.903109
34	1	0	6.837803	-2.316776	-0.875361
35	1	0	7.796866	-2.781706	0.537238
36	6	0	-4.087387	-0.961877	-0.003198
37	6	0	-4.879333	0.156484	0.205546
38	6	0	-6.258526	-0.291372	0.174004
39	6	0	-6.238222	-1.647269	-0.048610
40	6	0	-5.685157	2.418437	0.964283
41	1	0	-5.833210	2.181006	2.028948
42	1	0	-5.451963	3.490140	0.903133
43	6	0	-6.993600	2.115064	0.195268
44	1	0	-6.837798	2.316783	-0.875353
45	1	0	-7.796868	2.781702	0.537244
46	6	0	-4.490257	1.601890	0.408780
47	1	0	-3.623348	1.678555	1.075798
48	1	0	-4.172483	2.035933	-0.552031
49	6	0	-7.429552	0.639832	0.391301
50	1	0	-8.258269	0.391834	-0.285398

51	1	0	-7.813548	0.518390	1.415916
52	8	0	-4.913000	-2.093877	-0.162288
53	1	0	-6.996784	-2.406516	-0.147371
54	6	0	-2.671134	-1.188742	-0.087884
55	6	0	-1.882620	-2.324429	-0.192846
56	8	0	-1.843888	-0.046648	-0.067689
57	6	0	-0.508193	-1.887982	-0.245540
58	6	0	-2.289532	-3.776840	-0.271984
59	6	0	-0.511670	-0.502587	-0.165366
60	6	0	0.654448	-2.845434	-0.358579
61	6	0	-1.072868	-4.701145	-0.013666
62	1	0	-3.099235	-3.989469	0.436095
63	1	0	-2.701426	-3.988063	-1.270667
64	6	0	0.167647	-4.243264	-0.816832
65	1	0	1.412368	-2.450539	-1.045624
66	1	0	1.151002	-2.938432	0.619688
67	1	0	-0.826274	-4.698052	1.059205
68	1	0	-1.337639	-5.733291	-0.280472
69	1	0	-0.088487	-4.213990	-1.886969
70	1	0	0.983964	-4.969191	-0.700253

4CHS

Center Number	Atomic Number	Atomic Type	Coordinates (Angstroms) X	Y	Z
1	6	0	3.238946	0.399792	0.397007
2	6	0	2.683103	1.013060	1.510103

3	6	0	1.252851	1.227389	1.414273
4	6	0	0.695960	0.803508	0.216261
5	6	0	2.571709	1.712898	3.954553
6	1	0	2.205076	0.759187	4.363672
7	1	0	3.160544	2.198215	4.744760
8	6	0	1.367352	2.592973	3.558922
9	1	0	1.733857	3.531201	3.115446
10	1	0	0.779494	2.863421	4.446548
11	6	0	3.478775	1.453332	2.730160
12	1	0	4.249499	0.708703	2.966799
13	1	0	4.017349	2.383736	2.494588
14	6	0	0.458735	1.848703	2.554129
15	1	0	-0.312517	2.519629	2.155024
16	1	0	-0.079342	1.055629	3.095177
17	6	0	4.629501	0.071998	0.107335
18	6	0	5.183139	-1.107702	-0.368409
19	6	0	6.618456	-1.036086	-0.583774
20	6	0	4.401817	-2.380421	-0.658536
21	6	0	7.147834	0.199006	-0.291611
22	6	0	7.435548	-2.239257	-1.029735
23	6	0	5.323908	-3.620740	-0.727295
24	1	0	3.614202	-2.522395	0.092236
25	1	0	3.885721	-2.277215	-1.625241
26	1	0	8.179884	0.516406	-0.350094
27	6	0	6.544400	-3.349873	-1.634121
28	1	0	8.205922	-1.930736	-1.749092
29	1	0	7.968189	-2.653947	-0.159721
30	1	0	5.673696	-3.887257	0.281715
31	1	0	4.749344	-4.476999	-1.105961

32	1	0	6.193310	-3.049533	-2.632869
33	1	0	7.138092	-4.265366	-1.761748
34	6	0	-4.629501	0.071998	-0.107335
35	6	0	-5.183139	-1.107702	0.368409
36	6	0	-6.618456	-1.036086	0.583774
37	6	0	-7.147834	0.199006	0.291611
38	6	0	-5.323908	-3.620740	0.727296
39	1	0	-5.673696	-3.887257	-0.281714
40	1	0	-4.749344	-4.476999	1.105962
41	6	0	-6.544400	-3.349872	1.634122
42	1	0	-6.193310	-3.049533	2.632870
43	1	0	-7.138092	-4.265366	1.761749
44	6	0	-4.401818	-2.380421	0.658537
45	1	0	-3.614202	-2.522395	-0.092236
46	1	0	-3.885721	-2.277215	1.625242
47	6	0	-7.435548	-2.239257	1.029735
48	1	0	-8.205922	-1.930735	1.749092
49	1	0	-7.968189	-2.653947	0.159722
50	1	0	-8.179884	0.516407	0.350094
51	6	0	-3.238946	0.399792	-0.397007
52	6	0	-2.683103	1.013059	-1.510103
53	6	0	-1.252851	1.227389	-1.414273
54	6	0	-3.478775	1.453331	-2.730160
55	6	0	-0.695960	0.803508	-0.216261
56	6	0	-0.458735	1.848702	-2.554130
57	6	0	-2.571709	1.712897	-3.954553
58	1	0	-4.249499	0.708702	-2.966799
59	1	0	-4.017349	2.383736	-2.494588
60	6	0	-1.367352	2.592972	-3.558923

61	1	0	0.312517	2.519629	-2.155025
62	1	0	0.079342	1.055628	-3.095177
63	1	0	-2.205076	0.759186	-4.363672
64	1	0	-3.160544	2.198214	-4.744761
65	1	0	-1.733857	3.531200	-3.115447
66	1	0	-0.779494	2.863420	-4.446549
67	16	0	-5.912872	1.357985	-0.274653
68	16	0	-1.968015	0.079256	0.859724
69	16	0	1.968015	0.079255	-0.859724
70	16	0	5.912872	1.357985	0.274653

4CHSe

Center Number	Atomic Number	Atomic Type	Coordinates (Angstroms) X	Y	Z
1	6	0	-3.333804	0.260380	-0.250796
2	6	0	-2.810910	1.014239	-1.289189
3	6	0	-1.375168	1.240046	-1.244913
4	6	0	-0.715605	0.704672	-0.149704
5	6	0	-2.844641	2.012160	-3.637684
6	1	0	-2.501897	1.117244	-4.179283
7	1	0	-3.481094	2.583124	-4.327430
8	6	0	-1.620212	2.848341	-3.211526
9	1	0	-1.960885	3.724868	-2.639450
10	1	0	-1.084818	3.226232	-4.093154
11	6	0	-3.673877	1.600727	-2.400628
12	1	0	-4.462375	0.889269	-2.676623

13	1	0	-4.192042	2.494863	-2.019540
14	6	0	-0.655895	1.996311	-2.356368
15	1	0	0.142346	2.617480	-1.930905
16	1	0	-0.156031	1.272182	-3.018957
17	6	0	-4.726957	-0.091760	0.018003
18	6	0	-5.312327	-1.346228	0.077265
19	6	0	-6.726155	-1.361219	0.427377
20	6	0	-4.550158	-2.640632	-0.179816
21	6	0	-7.270466	-0.123626	0.678481
22	6	0	-7.536399	-2.652148	0.448744
23	6	0	-5.493717	-3.822619	-0.500804
24	1	0	-3.819370	-2.494101	-0.985121
25	1	0	-3.965976	-2.898236	0.717872
26	1	0	-8.297817	0.096388	0.943013
27	6	0	-6.636564	-3.906672	0.533743
28	1	0	-8.252610	-2.635577	1.281066
29	1	0	-8.132232	-2.714289	-0.475540
30	1	0	-5.923508	-3.700613	-1.506794
31	1	0	-4.914445	-4.755997	-0.508375
32	1	0	-6.205923	-3.994947	1.542744
33	1	0	-7.245264	-4.805014	0.361979
34	6	0	4.726957	-0.091760	-0.018003
35	6	0	5.312327	-1.346228	-0.077265
36	6	0	6.726155	-1.361219	-0.427378
37	6	0	7.270465	-0.123626	-0.678482
38	6	0	5.493717	-3.822619	0.500802
39	1	0	5.923508	-3.700613	1.506793
40	1	0	4.914445	-4.755997	0.508373
41	6	0	6.636564	-3.906672	-0.533744

42	1	0	6.205923	-3.994947	-1.542746
43	1	0	7.245264	-4.805014	-0.361981
44	6	0	4.550158	-2.640632	0.179815
45	1	0	3.819370	-2.494101	0.985121
46	1	0	3.965976	-2.898235	-0.717873
47	6	0	7.536399	-2.652147	-0.448745
48	1	0	8.252610	-2.635576	-1.281067
49	1	0	8.132232	-2.714289	0.475539
50	1	0	8.297817	0.096389	-0.943013
51	6	0	3.333804	0.260380	0.250796
52	6	0	2.810910	1.014238	1.289190
53	6	0	1.375168	1.240045	1.244914
54	6	0	3.673877	1.600725	2.400629
55	6	0	0.715605	0.704672	0.149704
56	6	0	0.655895	1.996309	2.356370
57	6	0	2.844641	2.012157	3.637685
58	1	0	4.462375	0.889267	2.676624
59	1	0	4.192042	2.494862	2.019542
60	6	0	1.620212	2.848338	3.211528
61	1	0	-0.142346	2.617478	1.930907
62	1	0	0.156031	1.272179	3.018958
63	1	0	2.501897	1.117241	4.179284
64	1	0	3.481094	2.583120	4.327432
65	1	0	1.960885	3.724866	2.639452
66	1	0	1.084819	3.226228	4.093156
67	34	0	6.011977	1.288285	-0.480967
68	34	0	1.948639	-0.241657	-1.000219
69	34	0	-1.948639	-0.241658	1.000219
70	34	0	-6.011977	1.288285	0.480967

4CHTe

Center Number	Atomic Number	Atomic Type	Coordinates (Angstroms) X	Y	Z
1	6	0	3.464486	-0.171081	-0.143685
2	6	0	2.910045	-1.079404	-1.030116
3	6	0	1.458245	-1.254558	-1.012506
4	6	0	0.728009	-0.523501	-0.090147
5	6	0	3.006516	-2.523767	-3.145226
6	1	0	2.731015	-1.740484	-3.868014
7	1	0	3.651341	-3.238845	-3.674350
8	6	0	1.727406	-3.217834	-2.637240
9	1	0	2.000482	-3.982461	-1.893873
10	1	0	1.214957	-3.735609	-3.459667
11	6	0	3.789591	-1.908032	-1.965299
12	1	0	4.624251	-1.296630	-2.329110
13	1	0	4.247250	-2.725935	-1.385342
14	6	0	0.767661	-2.183659	-2.009776
15	1	0	-0.080717	-2.682956	-1.526029
16	1	0	0.335091	-1.573505	-2.819401
17	6	0	4.885368	0.154521	0.019706
18	6	0	5.585802	1.182094	-0.590952
19	6	0	7.003857	1.326406	-0.259162
20	6	0	4.897241	2.168032	-1.533023
21	6	0	7.522802	0.443273	0.659187
22	6	0	7.872378	2.381309	-0.942666

23	6	0	5.892077	2.962372	-2.408833
24	1	0	4.165857	1.640595	-2.157706
25	1	0	4.315538	2.882593	-0.928308
26	1	0	8.553535	0.432852	0.996874
27	6	0	7.040983	3.532344	-1.551712
28	1	0	8.612405	2.773122	-0.232471
29	1	0	8.441176	1.900007	-1.754177
30	1	0	6.313722	2.310461	-3.189298
31	1	0	5.355081	3.772340	-2.921314
32	1	0	6.621976	4.161881	-0.752118
33	1	0	7.694219	4.172958	-2.160161
34	6	0	-4.885368	0.154521	-0.019706
35	6	0	-5.585801	1.182094	0.590952
36	6	0	-7.003857	1.326406	0.259162
37	6	0	-7.522802	0.443273	-0.659186
38	6	0	-5.892076	2.962373	2.408832
39	1	0	-6.313722	2.310462	3.189298
40	1	0	-5.355081	3.772341	2.921313
41	6	0	-7.040983	3.532345	1.551711
42	1	0	-6.621976	4.161882	0.752117
43	1	0	-7.694218	4.172958	2.160161
44	6	0	-4.897240	2.168032	1.533023
45	1	0	-4.165857	1.640595	2.157706
46	1	0	-4.315538	2.882593	0.928307
47	6	0	-7.872377	2.381309	0.942666
48	1	0	-8.612405	2.773122	0.232471
49	1	0	-8.441176	1.900008	1.754177
50	1	0	-8.553535	0.432852	-0.996873
51	6	0	-3.464486	-0.171081	0.143685

52	6	0	-2.910045	-1.079404	1.030117
53	6	0	-1.458245	-1.254558	1.012506
54	6	0	-3.789591	-1.908032	1.965299
55	6	0	-0.728009	-0.523501	0.090147
56	6	0	-0.767660	-2.183659	2.009776
57	6	0	-3.006515	-2.523767	3.145226
58	1	0	-4.624251	-1.296629	2.329110
59	1	0	-4.247250	-2.725935	1.385342
60	6	0	-1.727406	-3.217833	2.637241
61	1	0	0.080717	-2.682955	1.526030
62	1	0	-0.335091	-1.573504	2.819401
63	1	0	-2.731015	-1.740483	3.868014
64	1	0	-3.651341	-3.238845	3.674350
65	1	0	-2.000482	-3.982461	1.893874
66	1	0	-1.214957	-3.735608	3.459668
67	52	0	-6.128057	-0.927857	-1.342786
68	52	0	-1.982034	0.720136	-1.058581
69	52	0	1.982034	0.720136	1.058581
70	52	0	6.128057	-0.927857	1.342786

Optimization of 5CHX

5CHO

Center Number	Atomic Number	Atomic Type	Coordinates (Angstroms) X	Y	Z
1	6	0	4.672315	0.725118	-0.188790

2	6	0	4.251256	2.032947	-0.376628
3	6	0	2.808557	2.022709	-0.375050
4	6	0	2.405338	0.708952	-0.180214
5	6	0	4.186766	4.549342	-0.385181
6	1	0	3.989509	4.700863	0.687192
7	1	0	4.734738	5.432779	-0.739931
8	6	0	2.838059	4.421758	-1.132304
9	1	0	3.035008	4.237160	-2.199436
10	1	0	2.277307	5.364107	-1.066196
11	6	0	5.065983	3.288298	-0.580808
12	1	0	5.927438	3.303347	0.097347
13	1	0	5.485490	3.291267	-1.598456
14	6	0	1.976995	3.270702	-0.553943
15	1	0	1.112560	3.067585	-1.197367
16	1	0	1.564327	3.580998	0.418453
17	8	0	3.545908	-0.114443	-0.063189
18	6	0	5.960504	0.094586	-0.104886
19	6	0	6.394411	-1.189329	0.186533
20	8	0	7.076776	0.914151	-0.370573
21	6	0	7.841986	-1.175711	0.097841
22	6	0	5.603961	-2.433441	0.517057
23	6	0	8.214371	0.103707	-0.238149
24	6	0	8.693321	-2.393647	0.376090
25	6	0	6.525123	-3.523854	1.121761
26	1	0	4.780221	-2.197289	1.201226
27	1	0	5.134300	-2.825058	-0.398694
28	1	0	9.158902	0.593111	-0.411226
29	6	0	7.833897	-3.682818	0.311029
30	1	0	9.531562	-2.455661	-0.330790

31	1	0	9.135230	-2.314389	1.381306
32	1	0	6.777964	-3.260980	2.160390
33	1	0	5.983664	-4.479067	1.154420
34	1	0	7.584089	-3.909747	-0.736486
35	1	0	8.417091	-4.530261	0.696143
36	6	0	-2.405349	0.708950	0.180267
37	6	0	-2.808573	2.022704	0.375114
38	6	0	-4.251272	2.032941	0.376647
39	6	0	-4.672323	0.725114	0.188778
40	6	0	-2.838101	4.421757	1.132354
41	1	0	-3.035085	4.237163	2.199480
42	1	0	-2.277347	5.364106	1.066260
43	6	0	-4.186783	4.549338	0.385186
44	1	0	-3.989490	4.700853	-0.687181
45	1	0	-4.734767	5.432777	0.739915
46	6	0	-1.977018	3.270698	0.554026
47	1	0	-1.112603	3.067585	1.197478
48	1	0	-1.564320	3.580989	-0.418359
49	6	0	-5.066007	3.288295	0.580787
50	1	0	-5.927435	3.303340	-0.097401
51	1	0	-5.485551	3.291271	1.598420
52	8	0	-3.545912	-0.114456	0.063269
53	6	0	-1.131119	0.058855	0.083949
54	6	0	-0.718736	-1.266435	0.055335
55	8	0	-0.000006	0.898330	0.000019
56	6	0	0.718727	-1.266435	-0.055244
57	6	0	-1.540440	-2.532610	0.107540
58	6	0	1.131109	0.058855	-0.083887
59	6	0	1.540432	-2.532612	-0.107387

60	6	0	-0.642818	-3.753509	0.430520
61	1	0	-2.348675	-2.436597	0.842463
62	1	0	-2.032323	-2.692156	-0.864376
63	6	0	0.642814	-3.753519	-0.430347
64	1	0	2.348687	-2.436626	-0.842288
65	1	0	2.032282	-2.692127	0.864553
66	1	0	-0.361743	-3.735726	1.494685
67	1	0	-1.211631	-4.678224	0.262643
68	1	0	0.361744	-3.735756	-1.494514
69	1	0	1.211627	-4.678229	-0.262447
70	6	0	-8.214382	0.103707	0.237920
71	6	0	-7.841973	-1.175725	-0.097993
72	8	0	-7.076795	0.914151	0.370408
73	6	0	-6.394391	-1.189350	-0.186568
74	6	0	-8.693288	-2.393662	-0.376283
75	6	0	-5.960504	0.094579	0.104815
76	6	0	-5.603916	-2.433467	-0.517020
77	6	0	-7.833876	-3.682833	-0.311087
78	1	0	-9.531600	-2.455645	0.330513
79	1	0	-9.135092	-2.314438	-1.381549
80	6	0	-6.525041	-3.523909	-1.121730
81	1	0	-4.780155	-2.197325	-1.201172
82	1	0	-5.134276	-2.825049	0.398754
83	1	0	-7.584152	-3.909712	0.736458
84	1	0	-8.417042	-4.530292	-0.696210
85	1	0	-6.777811	-3.261085	-2.160389
86	1	0	-5.983580	-4.479124	-1.154307
87	1	0	-9.158925	0.593117	0.410914

5CHS

Center Number	Atomic Number	Atomic Type	Coordinates (Angstroms) X	Y	Z
1	6	0	5.163065	-0.699595	-0.203417
2	6	0	4.494321	-1.626681	-0.989518
3	6	0	3.054992	-1.616570	-0.820216
4	6	0	2.601181	-0.697881	0.115793
5	6	0	4.198858	-3.203139	-2.966012
6	1	0	3.920517	-2.447622	-3.716396
7	1	0	4.695797	-4.023033	-3.502214
8	6	0	2.924739	-3.711790	-2.259598
9	1	0	3.206246	-4.439600	-1.483505
10	1	0	2.271946	-4.234975	-2.971483
11	6	0	5.182098	-2.596700	-1.939110
12	1	0	6.017955	-2.098779	-2.446921
13	1	0	5.626203	-3.418916	-1.357760
14	6	0	2.148395	-2.533842	-1.628170
15	1	0	1.326575	-2.900870	-1.000355
16	1	0	1.677277	-1.956165	-2.437841
17	6	0	6.592427	-0.452002	-0.061021
18	6	0	7.291378	0.746610	-0.065674
19	6	0	8.718726	0.593138	0.159523
20	6	0	6.666722	2.117861	-0.275690
21	6	0	9.097818	-0.714309	0.354127
22	6	0	9.680648	1.770897	0.118574
23	6	0	7.719927	3.176558	-0.679736

24	1	0	5.866231	2.060575	-1.024066
25	1	0	6.186461	2.444957	0.659118
26	1	0	10.091093	-1.105237	0.526916
27	6	0	8.943426	3.123244	0.261228
28	1	0	10.444869	1.665452	0.900223
29	1	0	10.215121	1.764622	-0.844189
30	1	0	8.050834	3.003542	-1.715130
31	1	0	7.258998	4.173235	-0.650852
32	1	0	8.607299	3.261558	1.299903
33	1	0	9.637983	3.943534	0.034103
34	6	0	-2.601180	0.697881	0.115794
35	6	0	-3.054992	1.616572	-0.820213
36	6	0	-4.494320	1.626683	-0.989515
37	6	0	-5.163064	0.699595	-0.203416
38	6	0	-2.924739	3.711796	-2.259589
39	1	0	-3.206246	4.439604	-1.483494
40	1	0	-2.271946	4.234983	-2.971473
41	6	0	-4.198857	3.203146	-2.966005
42	1	0	-3.920516	2.447631	-3.716390
43	1	0	-4.695797	4.023042	-3.502204
44	6	0	-2.148395	2.533846	-1.628164
45	1	0	-1.326575	2.900873	-1.000347
46	1	0	-1.677276	1.956171	-2.437836
47	6	0	-5.182098	2.596705	-1.939104
48	1	0	-6.017955	2.098785	-2.446916
49	1	0	-5.626202	3.418920	-1.357752
50	6	0	-1.238636	0.364384	0.511144
51	6	0	-0.697081	0.197722	1.778052
52	6	0	0.697081	-0.197727	1.778051

53	6	0	-1.475167	0.386623	3.072293
54	6	0	1.238637	-0.364386	0.511144
55	6	0	1.475166	-0.386632	3.072292
56	6	0	-0.543197	0.547881	4.294698
57	1	0	-2.153170	1.244905	2.983350
58	1	0	-2.117289	-0.491275	3.240142
59	6	0	0.543195	-0.547894	4.294697
60	1	0	2.153169	-1.244915	2.983347
61	1	0	2.117288	0.491264	3.240144
62	1	0	-0.060912	1.537000	4.275909
63	1	0	-1.141487	0.497836	5.214547
64	1	0	0.060910	-1.537014	4.275904
65	1	0	1.141485	-0.497852	5.214546
66	6	0	-9.097817	0.714309	0.354127
67	6	0	-8.718726	-0.593137	0.159522
68	6	0	-7.291378	-0.746610	-0.065676
69	6	0	-9.680648	-1.770896	0.118571
70	6	0	-6.592427	0.452002	-0.061021
71	6	0	-6.666723	-2.117860	-0.275694
72	6	0	-8.943426	-3.123244	0.261223
73	1	0	-10.444869	-1.665453	0.900220
74	1	0	-10.215122	-1.764620	-0.844192
75	6	0	-7.719928	-3.176557	-0.679741
76	1	0	-5.866231	-2.060573	-1.024069
77	1	0	-6.186462	-2.444958	0.659113
78	1	0	-8.607299	-3.261559	1.299898
79	1	0	-9.637983	-3.943533	0.034097
80	1	0	-8.050835	-3.003538	-1.715136
81	1	0	-7.258999	-4.173234	-0.650860

82	1	0	-10.091093	1.105237	0.526917
83	16	0	-7.720757	1.847759	0.267543
84	16	0	-3.991785	-0.231267	0.825315
85	16	0	0.000000	0.000001	-0.766610
86	16	0	3.991785	0.231264	0.825316
87	16	0	7.720757	-1.847759	0.267541

5CHSe

Center Number	Atomic Number	Atomic Type	Coordinates (Angstroms) X	Y	Z
1	6	0	5.300430	-0.470349	-0.142812
2	6	0	4.663389	-1.624510	-0.568971
3	6	0	3.230077	-1.679227	-0.329810
4	6	0	2.688325	-0.586818	0.328332
5	6	0	4.456431	-3.736569	-1.985983
6	1	0	4.099615	-3.255856	-2.909727
7	1	0	5.009544	-4.637805	-2.283765
8	6	0	3.244849	-4.109898	-1.106644
9	1	0	3.602967	-4.559031	-0.167675
10	1	0	2.621624	-4.863005	-1.607823
11	6	0	5.404796	-2.784832	-1.222902
12	1	0	6.190576	-2.400654	-1.885532
13	1	0	5.922776	-3.365294	-0.443161
14	6	0	2.389709	-2.858688	-0.805873
15	1	0	1.610413	-3.085611	-0.067404
16	1	0	1.859457	-2.572862	-1.728116

17	6	0	6.722844	-0.135808	-0.189417
18	6	0	7.353536	0.891421	-0.872389
19	6	0	8.791994	0.990208	-0.665134
20	6	0	6.616389	1.879115	-1.768706
21	6	0	9.313215	0.062763	0.206150
22	6	0	9.644463	2.009791	-1.411706
23	6	0	7.568407	2.607179	-2.745342
24	1	0	5.813947	1.366680	-2.314192
25	1	0	6.121358	2.635530	-1.139210
26	1	0	10.351355	-0.051104	0.494510
27	6	0	8.798380	3.164219	-1.996801
28	1	0	10.428024	2.404082	-0.750971
29	1	0	10.161176	1.502712	-2.241792
30	1	0	7.905375	1.914348	-3.531513
31	1	0	7.022862	3.420090	-3.243702
32	1	0	8.459536	3.830983	-1.189379
33	1	0	9.420272	3.765952	-2.673703
34	6	0	-2.688325	0.586816	0.328334
35	6	0	-3.230077	1.679227	-0.329805
36	6	0	-4.663388	1.624512	-0.568966
37	6	0	-5.300430	0.470350	-0.142811
38	6	0	-3.244847	4.109901	-1.106631
39	1	0	-3.602966	4.559031	-0.167660
40	1	0	-2.621622	4.863009	-1.607807
41	6	0	-4.456430	3.736576	-1.985971
42	1	0	-4.099613	3.255865	-2.909716
43	1	0	-5.009542	4.637813	-2.283751
44	6	0	-2.389708	2.858690	-0.805863
45	1	0	-1.610412	3.085610	-0.067394

46	1	0	-1.859456	2.572867	-1.728107
47	6	0	-5.404795	2.784837	-1.222893
48	1	0	-6.190575	2.400661	-1.885525
49	1	0	-5.922775	3.365296	-0.443150
50	6	0	-1.290008	0.317774	0.661147
51	6	0	-0.708514	0.163456	1.909255
52	6	0	0.708514	-0.163465	1.909255
53	6	0	-1.493190	0.296174	3.209571
54	6	0	1.290008	-0.317777	0.661145
55	6	0	1.493190	-0.296189	3.209570
56	6	0	-0.575563	0.513525	4.433478
57	1	0	-2.227998	1.106450	3.122479
58	1	0	-2.075226	-0.624559	3.373370
59	6	0	0.575563	-0.513545	4.433476
60	1	0	2.227997	-1.106464	3.122474
61	1	0	2.075226	0.624544	3.373373
62	1	0	-0.154340	1.530356	4.415829
63	1	0	-1.170733	0.426378	5.352650
64	1	0	0.154340	-1.530376	4.415823
65	1	0	1.170733	-0.426402	5.352648
66	6	0	-9.313215	-0.062761	0.206148
67	6	0	-8.791995	-0.990205	-0.665136
68	6	0	-7.353537	-0.891418	-0.872392
69	6	0	-9.644464	-2.009786	-1.411710
70	6	0	-6.722844	0.135810	-0.189418
71	6	0	-6.616390	-1.879112	-1.768709
72	6	0	-8.798381	-3.164213	-1.996807
73	1	0	-10.428025	-2.404078	-0.750976
74	1	0	-10.161176	-1.502706	-2.241796

75	6	0	-7.568408	-2.607173	-2.745347
76	1	0	-5.813947	-1.366676	-2.314194
77	1	0	-6.121360	-2.635527	-1.139215
78	1	0	-8.459537	-3.830979	-1.189386
79	1	0	-9.420273	-3.765945	-2.673710
80	1	0	-7.905375	-1.914340	-3.531517
81	1	0	-7.022863	-3.420083	-3.243708
82	1	0	-10.351355	0.051107	0.494508
83	34	0	-7.994760	1.120445	0.897923
84	34	0	-4.048328	-0.734835	0.703371
85	34	0	0.000000	0.000002	-0.743410
86	34	0	4.048328	0.734833	0.703374
87	34	0	7.994760	-1.120444	0.897922

5CHTe

Center Number	Atomic Number	Atomic Type	Coordinates (Angstroms) X	Y	Z
1	6	0	5.515857	0.094674	-0.286771
2	6	0	4.910817	0.057868	-1.532052
3	6	0	3.468868	-0.179731	-1.586002
4	6	0	2.800389	-0.383687	-0.390235
5	6	0	4.867189	0.578113	-4.040870
6	1	0	4.521070	1.620393	-3.965189
7	1	0	5.481370	0.507089	-4.949087
8	6	0	3.645325	-0.356698	-4.136764
9	1	0	3.990775	-1.400791	-4.185484

10	1	0	3.078964	-0.160816	-5.057593
11	6	0	5.729206	0.212731	-2.812945
12	1	0	6.522567	0.954286	-2.659953
13	1	0	6.244289	-0.739728	-3.018734
14	6	0	2.719010	-0.158632	-2.917165
15	1	0	1.922483	-0.912597	-2.907133
16	1	0	2.210511	0.813947	-3.019453
17	6	0	6.938490	0.306088	0.001284
18	6	0	7.571081	1.509959	0.265544
19	6	0	9.007645	1.474077	0.542478
20	6	0	6.794182	2.823929	0.325576
21	6	0	9.611590	0.238030	0.547130
22	6	0	9.801802	2.759769	0.767220
23	6	0	7.703671	4.069264	0.226693
24	1	0	6.026571	2.841488	-0.457875
25	1	0	6.247976	2.864389	1.281965
26	1	0	10.665268	0.062572	0.735683
27	6	0	8.905655	3.946641	1.185819
28	1	0	10.588143	2.587380	1.513862
29	1	0	10.313721	3.028287	-0.170643
30	1	0	8.074844	4.186209	-0.803102
31	1	0	7.114128	4.966444	0.460297
32	1	0	8.539246	3.801274	2.213364
33	1	0	9.498291	4.871883	1.180683
34	6	0	-2.800391	-0.383730	0.390206
35	6	0	-3.468855	-0.179784	1.585984
36	6	0	-4.910803	0.057822	1.532054
37	6	0	-5.515858	0.094644	0.286781
38	6	0	-3.645273	-0.356727	4.136749

39	1	0	-3.990727	-1.400818	4.185481
40	1	0	-3.078897	-0.160842	5.057568
41	6	0	-4.867133	0.578091	4.040867
42	1	0	-4.521010	1.620368	3.965175
43	1	0	-5.481301	0.507076	4.949094
44	6	0	-2.718976	-0.158673	2.917135
45	1	0	-1.922449	-0.912638	2.907097
46	1	0	-2.210476	0.813907	3.019406
47	6	0	-5.729171	0.212706	2.812958
48	1	0	-6.522526	0.954269	2.659970
49	1	0	-6.244262	-0.739745	3.018763
50	6	0	-1.364405	-0.621154	0.205851
51	6	0	-0.724221	-1.844739	0.101003
52	6	0	0.724237	-1.844729	-0.101063
53	6	0	-1.510022	-3.154251	0.149459
54	6	0	1.364403	-0.621134	-0.205904
55	6	0	1.510057	-3.154229	-0.149502
56	6	0	-0.623061	-4.381162	0.453581
57	1	0	-2.324107	-3.072187	0.879905
58	1	0	-1.997239	-3.309161	-0.827032
59	6	0	0.623117	-4.381156	-0.453616
60	1	0	2.324145	-3.072160	-0.879946
61	1	0	1.997273	-3.309122	0.826992
62	1	0	-0.304379	-4.367533	1.507200
63	1	0	-1.209857	-5.298158	0.305697
64	1	0	0.304434	-4.367540	-1.507234
65	1	0	1.209926	-5.298142	-0.305724
66	6	0	-9.611604	0.238108	-0.547039
67	6	0	-9.007621	1.474137	-0.542422

68	6	0	-7.571050	1.509981	-0.265521
69	6	0	-9.801747	2.759850	-0.767160
70	6	0	-6.938489	0.306096	-0.001255
71	6	0	-6.794115	2.823928	-0.325584
72	6	0	-8.905574	3.946691	-1.185792
73	1	0	-10.588110	2.587476	-1.513781
74	1	0	-10.313635	3.028393	0.170713
75	6	0	-7.703565	4.069291	-0.226695
76	1	0	-6.026487	2.841474	0.457851
77	1	0	-6.247927	2.864362	-1.281984
78	1	0	-8.539192	3.801302	-2.213344
79	1	0	-9.498183	4.871950	-1.180654
80	1	0	-8.074712	4.186259	0.803107
81	1	0	-7.114002	4.966451	-0.460322
82	1	0	-10.665292	0.062680	-0.735561
83	52	0	-8.298661	-1.306892	-0.121099
84	52	0	-4.116769	-0.251368	-1.249140
85	52	0	-0.000017	0.970622	-0.000058
86	52	0	4.116732	-0.251259	1.249135
87	52	0	8.298616	-1.306936	0.121160

Optimization of 6CHX

6CHO

Center Number	Atomic Number	Atomic Type	Coordinates (Angstroms) X	Y	Z

1	6	0	6.376029	1.022184	-0.143374
2	6	0	5.827554	2.263395	-0.429281
3	6	0	4.395436	2.097042	-0.481118
4	6	0	4.125216	0.761035	-0.218689
5	6	0	5.497794	4.753170	-0.599498
6	1	0	5.238712	4.945351	0.453020
7	1	0	5.964296	5.668275	-0.988771
8	6	0	4.204249	4.436536	-1.386904
9	1	0	4.466278	4.211934	-2.432143
10	1	0	3.544567	5.314875	-1.401457
11	6	0	6.513093	3.585370	-0.681923
12	1	0	7.337422	3.733482	0.025678
13	1	0	6.974312	3.573750	-1.681296
14	6	0	3.445232	3.234967	-0.769627
15	1	0	2.636475	2.901927	-1.431020
16	1	0	2.959641	3.555480	0.165071
17	8	0	5.340404	0.074504	-0.007193
18	6	0	7.718785	0.540978	0.028258
19	6	0	8.273523	-0.670973	0.409369
20	8	0	8.752208	1.462560	-0.238462
21	6	0	9.713881	-0.503835	0.378629
22	6	0	7.606512	-1.975735	0.776295
23	6	0	9.962543	0.789262	-0.014421
24	6	0	10.676872	-1.605109	0.759884
25	6	0	8.609809	-2.926224	1.478555
26	1	0	6.730824	-1.796861	1.411491
27	1	0	7.225495	-2.462926	-0.134778
28	1	0	10.856360	1.369149	-0.176999
29	6	0	9.964727	-2.982375	0.732549

30	1	0	11.549180	-1.611274	0.092639
31	1	0	11.060098	-1.425946	1.776286
32	1	0	8.783992	-2.584386	2.510346
33	1	0	8.172838	-3.932031	1.542416
34	1	0	9.790194	-3.289186	-0.309848
35	1	0	10.616710	-3.739709	1.188556
36	6	0	-0.669767	0.254512	-0.050781
37	6	0	-1.222653	1.525680	0.030693
38	6	0	-2.655148	1.374846	-0.023033
39	6	0	-2.924394	0.016814	-0.128481
40	6	0	-1.546580	3.958747	0.585081
41	1	0	-1.775068	3.839779	1.655304
42	1	0	-1.088085	4.949317	0.461446
43	6	0	-2.862836	3.874587	-0.223599
44	1	0	-2.630647	3.958277	-1.296364
45	1	0	-3.518976	4.718059	0.031056
46	6	0	-0.539287	2.867943	0.142267
47	1	0	0.306650	2.811871	0.837823
48	1	0	-0.110913	3.145252	-0.833220
49	6	0	-3.609033	2.543813	0.046470
50	1	0	-4.435351	2.408460	-0.661592
51	1	0	-4.069671	2.583629	1.045598
52	8	0	-1.707311	-0.697095	-0.148676
53	6	0	0.669765	-0.254570	-0.050783
54	6	0	1.222657	-1.525735	0.030708
55	8	0	1.707307	0.697038	-0.148693
56	6	0	2.655154	-1.374895	-0.023024
57	6	0	0.539298	-2.867997	0.142337
58	6	0	2.924393	-0.016862	-0.128482

59	6	0	3.609044	-2.543856	0.046520
60	6	0	1.546600	-3.958782	0.585180
61	1	0	-0.306633	-2.811901	0.837899
62	1	0	0.110914	-3.145342	-0.833134
63	6	0	2.862854	-3.874640	-0.223508
64	1	0	4.435359	-2.408521	-0.661545
65	1	0	4.069682	-2.583638	1.045651
66	1	0	1.775095	-3.839779	1.655398
67	1	0	1.088111	-4.949359	0.461581
68	1	0	2.630664	-3.958363	-1.296271
69	1	0	3.519001	-4.718098	0.031171
70	6	0	-7.718790	-0.540943	0.028266
71	6	0	-8.273496	0.671024	0.409365
72	6	0	-9.713859	0.503917	0.378649
73	6	0	-9.962555	-0.789175	-0.014399
74	6	0	-8.609722	2.926273	1.478579
75	1	0	-8.783900	2.584432	2.510370
76	1	0	-8.172731	3.932072	1.542443
77	6	0	-9.964647	2.982456	0.732591
78	1	0	-9.790123	3.289270	-0.309807
79	1	0	-10.616610	3.739798	1.188612
80	6	0	-7.606451	1.975767	0.776300
81	1	0	-6.730760	1.796867	1.411486
82	1	0	-7.225431	2.462959	-0.134771
83	6	0	-10.676820	1.605206	0.759931
84	1	0	-11.549136	1.611395	0.092698
85	1	0	-11.060036	1.426043	1.776337
86	8	0	-8.752238	-1.462504	-0.238437
87	6	0	-6.376042	-1.022180	-0.143372

88	6	0	-5.827590	-2.263403	-0.429270
89	8	0	-5.340401	-0.074521	-0.007188
90	6	0	-4.395468	-2.097071	-0.481121
91	6	0	-6.513151	-3.585364	-0.681925
92	6	0	-4.125225	-0.761071	-0.218687
93	6	0	-3.445286	-3.235004	-0.769673
94	6	0	-5.497868	-4.753177	-0.599534
95	1	0	-7.337471	-3.733477	0.025684
96	1	0	-6.974381	-3.573716	-1.681295
97	6	0	-4.204334	-4.436550	-1.386959
98	1	0	-2.636535	-2.901961	-1.431074
99	1	0	-2.959684	-3.555545	0.165007
100	1	0	-5.238771	-4.945380	0.452977
101	1	0	-5.964391	-5.668268	-0.988814
102	1	0	-4.466382	-4.211923	-2.432189
103	1	0	-3.544668	-5.314901	-1.401545
104	1	0	-10.856388	-1.369039	-0.176972

6CHS

Center Number	Atomic Number	Atomic Type	Coordinates (Angstroms) X	Y	Z
1	6	0	-7.182911	-0.421828	-0.138504
2	6	0	-6.652170	-1.656228	0.205869
3	6	0	-5.210878	-1.736906	0.076268
4	6	0	-4.618583	-0.572875	-0.392484
5	6	0	-6.623424	-3.922315	1.363925
6	1	0	-6.319260	-3.572967	2.362317

7	1	0	-7.226029	-4.829252	1.508881
8	6	0	-5.363882	-4.236876	0.529674
9	1	0	-5.667108	-4.550648	-0.480711
10	1	0	-4.806315	-5.072714	0.973647
11	6	0	-7.483212	-2.846337	0.662397
12	1	0	-8.296720	-2.509269	1.317427
13	1	0	-7.967969	-3.306191	-0.212117
14	6	0	-4.443738	-2.997481	0.448389
15	1	0	-3.625775	-3.165583	-0.263627
16	1	0	-3.967208	-2.855003	1.430149
17	6	0	-8.571808	0.018228	-0.186060
18	6	0	-9.155035	1.172837	0.315228
19	6	0	-10.573791	1.287031	0.022429
20	6	0	-8.419181	2.250000	1.097959
21	6	0	-11.059980	0.233021	-0.714966
22	6	0	-11.422303	2.437203	0.543211
23	6	0	-9.392105	3.150018	1.895925
24	1	0	-7.675500	1.794737	1.764160
25	1	0	-7.853462	2.884399	0.398686
26	1	0	-12.072680	0.063280	-1.053965
27	6	0	-10.554523	3.633132	1.001124
28	1	0	-12.142495	2.755913	-0.222219
29	1	0	-12.014358	2.086154	1.402759
30	1	0	-9.801197	2.595454	2.754159
31	1	0	-8.839485	4.008731	2.300745
32	1	0	-10.143085	4.157306	0.125376
33	1	0	-11.182316	4.353212	1.543558
34	6	0	0.680704	0.137214	0.260117
35	6	0	1.159209	0.547918	1.496569

36	6	0	2.581297	0.337796	1.679752
37	6	0	3.209810	-0.259695	0.596450
38	6	0	1.132036	1.883813	3.663527
39	1	0	1.522173	2.829678	3.257987
40	1	0	0.487584	2.139275	4.515613
41	6	0	2.312191	1.001818	4.122077
42	1	0	1.924380	0.040948	4.493058
43	1	0	2.847943	1.476987	4.955001
44	6	0	0.292297	1.151260	2.592362
45	1	0	-0.454650	1.825590	2.154597
46	1	0	-0.275928	0.349161	3.087282
47	6	0	3.295931	0.763698	2.954073
48	1	0	4.053867	0.019090	3.228531
49	1	0	3.843867	1.699535	2.766631
50	6	0	-0.680704	0.137214	-0.260118
51	6	0	-1.159209	0.547916	-1.496570
52	6	0	-2.581297	0.337794	-1.679753
53	6	0	-0.292297	1.151256	-2.592364
54	6	0	-3.209810	-0.259696	-0.596451
55	6	0	-3.295931	0.763693	-2.954075
56	6	0	-1.132036	1.883807	-3.663531
57	1	0	0.454650	1.825586	-2.154600
58	1	0	0.275928	0.349156	-3.087283
59	6	0	-2.312191	1.001811	-4.122079
60	1	0	-4.053867	0.019084	-3.228532
61	1	0	-3.843867	1.699531	-2.766635
62	1	0	-1.522173	2.829672	-3.257993
63	1	0	-0.487584	2.139267	-4.515617
64	1	0	-1.924380	0.040940	-4.493059

65	1	0	-2.847943	1.476979	-4.955004
66	6	0	8.571808	0.018228	0.186060
67	6	0	9.155035	1.172836	-0.315229
68	6	0	10.573791	1.287030	-0.022429
69	6	0	11.059980	0.233021	0.714967
70	6	0	9.392106	3.150016	-1.895927
71	1	0	9.801198	2.595451	-2.754161
72	1	0	8.839486	4.008729	-2.300749
73	6	0	10.554524	3.633131	-1.001127
74	1	0	10.143085	4.157305	-0.125380
75	1	0	11.182316	4.353210	-1.543562
76	6	0	8.419182	2.249999	-1.097961
77	1	0	7.675500	1.794735	-1.764161
78	1	0	7.853462	2.884399	-0.398688
79	6	0	11.422303	2.437202	-0.543212
80	1	0	12.142495	2.755913	0.222217
81	1	0	12.014359	2.086153	-1.402760
82	6	0	7.182911	-0.421828	0.138504
83	6	0	6.652170	-1.656229	-0.205868
84	6	0	5.210878	-1.736906	-0.076267
85	6	0	7.483212	-2.846337	-0.662394
86	6	0	4.618583	-0.572875	0.392484
87	6	0	4.443738	-2.997482	-0.448387
88	6	0	6.623424	-3.922316	-1.363921
89	1	0	8.296720	-2.509270	-1.317424
90	1	0	7.967968	-3.306191	0.212120
91	6	0	5.363882	-4.236876	-0.529670
92	1	0	3.625774	-3.165583	0.263630
93	1	0	3.967208	-2.855004	-1.430146

94	1	0	6.319260	-3.572969	-2.362313
95	1	0	7.226029	-4.829253	-1.508876
96	1	0	5.667108	-4.550648	0.480715
97	1	0	4.806315	-5.072715	-0.973642
98	1	0	12.072679	0.063280	1.053967
99	16	0	9.802184	-0.976439	1.094627
100	16	0	5.870931	0.712241	0.676545
101	16	0	2.021225	-0.569824	-0.741439
102	16	0	-2.021225	-0.569823	0.741440
103	16	0	-5.870931	0.712240	-0.676545
104	16	0	-9.802184	-0.976440	-1.094626

6CHSe

Center Number	Atomic Number	Atomic Type	Coordinates (Angstroms) X	Y	Z
1	6	0	-7.319237	0.239568	-0.374452
2	6	0	-6.711884	0.472442	-1.597778
3	6	0	-5.268909	0.648773	-1.562527
4	6	0	-4.687717	0.599129	-0.305589
5	6	0	-6.589307	0.397488	-4.145613
6	1	0	-6.274827	-0.652816	-4.243857
7	1	0	-7.162612	0.648800	-5.048376
8	6	0	-5.338807	1.293717	-4.030484
9	1	0	-5.653596	2.339997	-3.896950
10	1	0	-4.746219	1.250100	-4.954436
11	6	0	-7.492502	0.568630	-2.903632

12	1	0	-8.307062	-0.166615	-2.907447
13	1	0	-7.975732	1.556749	-2.961418
14	6	0	-4.461214	0.845900	-2.840349
15	1	0	-3.648115	1.561074	-2.662802
16	1	0	-3.975042	-0.105475	-3.108468
17	6	0	-8.739988	0.075824	-0.071170
18	6	0	-9.396834	-1.025122	0.454154
19	6	0	-10.823001	-0.848331	0.692015
20	6	0	-8.698053	-2.336893	0.790595
21	6	0	-11.307022	0.402141	0.387080
22	6	0	-11.704607	-1.990767	1.182934
23	6	0	-9.691567	-3.514746	0.916983
24	1	0	-7.926095	-2.555409	0.041992
25	1	0	-8.168518	-2.226156	1.750177
26	1	0	-12.330710	0.745681	0.475943
27	6	0	-10.881287	-3.132113	1.823525
28	1	0	-12.453961	-1.612964	1.891258
29	1	0	-12.262624	-2.403103	0.327495
30	1	0	-10.069758	-3.798408	-0.077067
31	1	0	-9.165809	-4.389277	1.323870
32	1	0	-10.500626	-2.817232	2.807027
33	1	0	-11.531565	-4.001556	1.991544
34	6	0	0.690791	-0.239677	0.500523
35	6	0	1.208814	-1.396772	1.059246
36	6	0	2.626747	-1.627048	0.832776
37	6	0	3.272465	-0.680830	0.053362
38	6	0	1.208176	-3.341453	2.711335
39	1	0	1.619966	-2.796798	3.574738
40	1	0	0.567427	-4.140200	3.109072

41	6	0	2.367768	-3.937031	1.886026
42	1	0	1.957756	-4.452370	1.003963
43	1	0	2.914171	-4.687353	2.473529
44	6	0	0.356994	-2.391437	1.839543
45	1	0	-0.380179	-1.854956	2.450194
46	1	0	-0.223759	-3.001261	1.129418
47	6	0	3.344796	-2.823984	1.445811
48	1	0	4.090851	-3.213993	0.741973
49	1	0	3.908779	-2.489627	2.330955
50	6	0	-0.690791	0.239677	0.500522
51	6	0	-1.208815	1.396772	1.059245
52	6	0	-2.626747	1.627048	0.832774
53	6	0	-0.356995	2.391437	1.839543
54	6	0	-3.272465	0.680830	0.053361
55	6	0	-3.344796	2.823983	1.445809
56	6	0	-1.208177	3.341453	2.711334
57	1	0	0.380178	1.854956	2.450193
58	1	0	0.223758	3.001260	1.129418
59	6	0	-2.367769	3.937030	1.886025
60	1	0	-4.090851	3.213992	0.741971
61	1	0	-3.908780	2.489627	2.330953
62	1	0	-1.619967	2.796798	3.574737
63	1	0	-0.567429	4.140199	3.109071
64	1	0	-1.957757	4.452370	1.003962
65	1	0	-2.914172	4.687353	2.473528
66	6	0	8.739988	-0.075824	-0.071165
67	6	0	9.396834	1.025120	0.454165
68	6	0	10.823001	0.848328	0.692026
69	6	0	11.307022	-0.402142	0.387084

70	6	0	9.691566	3.514741	0.917007
71	1	0	10.069757	3.798408	-0.077041
72	1	0	9.165807	4.389270	1.323899
73	6	0	10.881286	3.132104	1.823547
74	1	0	10.500625	2.817218	2.807048
75	1	0	11.531564	4.001546	1.991571
76	6	0	8.698052	2.336889	0.790613
77	1	0	7.926095	2.555408	0.042012
78	1	0	8.168517	2.226146	1.750195
79	6	0	11.704607	1.990761	1.182951
80	1	0	12.453960	1.612955	1.891273
81	1	0	12.262624	2.403102	0.327514
82	6	0	7.319237	-0.239567	-0.374447
83	6	0	6.711885	-0.472439	-1.597775
84	6	0	5.268910	-0.648770	-1.562524
85	6	0	7.492503	-0.568624	-2.903628
86	6	0	4.687717	-0.599129	-0.305587
87	6	0	4.461215	-0.845895	-2.840347
88	6	0	6.589309	-0.397478	-4.145609
89	1	0	8.307063	0.166621	-2.907442
90	1	0	7.975733	-1.556742	-2.961416
91	6	0	5.338808	-1.293708	-4.030483
92	1	0	3.648116	-1.561069	-2.662802
93	1	0	3.975042	0.105482	-3.108464
94	1	0	6.274828	0.652826	-4.243851
95	1	0	7.162614	-0.648788	-5.048372
96	1	0	5.653598	-2.339988	-3.896952
97	1	0	4.746220	-1.250089	-4.954435
98	1	0	12.330710	-0.745682	0.475945

99	34	0	9.963684	-1.567790	-0.286408
100	34	0	6.018528	-0.261600	1.055238
101	34	0	2.053405	0.723477	-0.475556
102	34	0	-2.053405	-0.723477	-0.475557
103	34	0	-6.018528	0.261598	1.055235
104	34	0	-9.963683	1.567792	-0.286405

6CHTe

Center Number	Atomic Number	Atomic Type	Coordinates (Angstroms) X	Y	Z
1	6	0	-7.574561	0.236362	-0.328187
2	6	0	-6.927427	1.138973	-1.155564
3	6	0	-5.482568	1.299491	-0.996424
4	6	0	-4.853551	0.561006	-0.007908
5	6	0	-6.802631	2.578306	-3.271847
6	1	0	-6.467876	1.788841	-3.962183
7	1	0	-7.384808	3.298527	-3.863021
8	6	0	-5.570556	3.260082	-2.644792
9	1	0	-5.905656	4.031323	-1.934332
10	1	0	-4.974782	3.768204	-3.415471
11	6	0	-7.703045	1.976555	-2.171076
12	1	0	-8.507772	1.375687	-2.611658
13	1	0	-8.202368	2.802122	-1.638184
14	6	0	-4.687947	2.218593	-1.922945
15	1	0	-3.886473	2.711894	-1.359694
16	1	0	-4.183832	1.600576	-2.683784

17	6	0	-9.007570	-0.075895	-0.305836
18	6	0	-9.659375	-1.076448	-1.008217
19	6	0	-11.103485	-1.211125	-0.812671
20	6	0	-8.896850	-2.045701	-1.909863
21	6	0	-11.695701	-0.347395	0.079421
22	6	0	-11.915955	-2.236072	-1.602307
23	6	0	-9.814548	-2.806019	-2.893522
24	1	0	-8.107106	-1.510985	-2.451828
25	1	0	-8.378917	-2.781715	-1.273715
26	1	0	-12.752991	-0.333299	0.321353
27	6	0	-11.043837	-3.382350	-2.161286
28	1	0	-12.723616	-2.635748	-0.975048
29	1	0	-12.400742	-1.726662	-2.450387
30	1	0	-10.155474	-2.129962	-3.692681
31	1	0	-9.240650	-3.609783	-3.374839
32	1	0	-10.707598	-4.036828	-1.342858
33	1	0	-11.644407	-3.999244	-2.843983
34	6	0	0.712320	-0.175071	0.666877
35	6	0	1.335811	-1.144321	1.434787
36	6	0	2.775675	-1.333166	1.261864
37	6	0	3.423338	-0.552466	0.319044
38	6	0	1.424075	-2.717457	3.456112
39	1	0	1.780560	-1.983233	4.194817
40	1	0	0.823160	-3.458193	4.001336
41	6	0	2.636965	-3.390233	2.783645
42	1	0	2.280977	-4.105071	2.025923
43	1	0	3.219399	-3.962366	3.518841
44	6	0	0.539111	-2.022317	2.398627
45	1	0	-0.247346	-1.428234	2.879729

46	1	0	0.015586	-2.798179	1.816465
47	6	0	3.546355	-2.328148	2.128253
48	1	0	4.335906	-2.804742	1.534609
49	1	0	4.064945	-1.772065	2.926250
50	6	0	-0.712321	0.175123	0.666867
51	6	0	-1.335816	1.144407	1.434730
52	6	0	-2.775681	1.333240	1.261795
53	6	0	-0.539121	2.022449	2.398532
54	6	0	-3.423339	0.552495	0.319010
55	6	0	-3.546365	2.328259	2.128137
56	6	0	-1.424090	2.717635	3.455983
57	1	0	0.247336	1.428390	2.879663
58	1	0	-0.015597	2.798286	1.816336
59	6	0	-2.636980	3.390377	2.783482
60	1	0	-4.335916	2.804824	1.534469
61	1	0	-4.064956	1.772211	2.926157
62	1	0	-1.780574	1.983444	4.194721
63	1	0	-0.823178	3.458397	4.001175
64	1	0	-2.280992	4.105181	2.025730
65	1	0	-3.219418	3.962541	3.518651
66	6	0	9.007572	0.075868	-0.305829
67	6	0	9.659382	1.076393	-1.008245
68	6	0	11.103493	1.211067	-0.812706
69	6	0	11.695705	0.347363	0.079413
70	6	0	9.814564	2.805897	-2.893610
71	1	0	10.155483	2.129810	-3.692747
72	1	0	9.240670	3.609649	-3.374955
73	6	0	11.043857	3.382246	-2.161397
74	1	0	10.707625	4.036755	-1.342992

75	1	0	11.644430	3.999112	-2.844117
76	6	0	8.896862	2.045621	-1.909923
77	1	0	8.107112	1.510892	-2.451867
78	1	0	8.378936	2.781661	-1.273799
79	6	0	11.915969	2.235982	-1.602380
80	1	0	12.723634	2.635674	-0.975135
81	1	0	12.400752	1.726539	-2.450442
82	6	0	7.574561	-0.236380	-0.328167
83	6	0	6.927421	-1.139019	-1.155509
84	6	0	5.482561	-1.299523	-0.996362
85	6	0	7.703034	-1.976644	-2.170990
86	6	0	4.853550	-0.560997	-0.007873
87	6	0	4.687934	-2.218657	-1.922846
88	6	0	6.802615	-2.578436	-3.271736
89	1	0	8.507762	-1.375797	-2.611598
90	1	0	8.202355	-2.802192	-1.638067
91	6	0	5.570537	-3.260180	-2.644652
92	1	0	3.886458	-2.711930	-1.359575
93	1	0	4.183823	-1.600667	-2.683709
94	1	0	6.467862	-1.788996	-3.962103
95	1	0	7.384787	-3.298683	-3.862881
96	1	0	5.905634	-4.031393	-1.934161
97	1	0	4.974759	-3.768330	-3.415309
98	1	0	12.752996	0.333268	0.321343
99	52	0	10.355604	-0.985353	0.928116
100	52	0	6.225574	0.669581	1.011846
101	52	0	2.083385	0.774011	-0.619982
102	52	0	-2.083381	-0.774020	-0.619953
103	52	0	-6.225567	-0.669542	1.011859

104	52	0	-10.355607	0.985359	0.928076

Optimization of PCHXs

PCHO

Center Number	Atomic Number	Atomic Type	Coordinates (Angstroms) X	Y	Z
1	6	0	0.646717	0.307432	-0.021579
2	6	0	1.093598	1.620361	-0.105912
3	6	0	2.533760	1.586048	-0.093321
4	6	0	2.916281	0.253068	-0.007748
5	6	0	1.201535	4.078559	-0.639558
6	1	0	1.401102	3.988673	-1.718378
7	1	0	0.668512	5.027083	-0.488141
8	6	0	2.547257	4.094317	0.122459
9	1	0	2.346916	4.151843	1.203327
10	1	0	3.125213	4.988931	-0.146551
11	6	0	0.302272	2.904989	-0.176677
12	1	0	-0.559813	2.788855	-0.843012
13	1	0	-0.110111	3.136350	0.817903
14	6	0	3.386772	2.829174	-0.185099
15	1	0	4.244921	2.757093	0.492401
16	1	0	3.805761	2.911709	-1.200194
17	8	0	1.761062	-0.557065	0.038948
18	6	0	-0.646612	-0.308223	0.022626
19	6	0	-1.093531	-1.620967	0.109312

20	8	0	-1.760888	0.557509	-0.019278
21	6	0	-2.533600	-1.584657	0.127878
22	6	0	-0.302349	-2.904645	0.196819
23	6	0	-2.916049	-0.251433	0.045628
24	6	0	-3.386852	-2.828908	0.201015
25	6	0	-1.204246	-4.126444	-0.109368
26	1	0	0.556139	-2.873984	-0.483394
27	1	0	0.115265	-3.009158	1.210474
28	6	0	-2.544276	-4.045956	0.658386
29	1	0	-4.239118	-2.671408	0.871464
30	1	0	-3.814692	-3.038286	-0.791904
31	1	0	-1.411605	-4.172025	-1.189481
32	1	0	-0.669805	-5.048857	0.155726
33	1	0	-2.335983	-3.967649	1.736457
34	1	0	-3.123867	-4.966931	0.507990
35	-2	0	7.093984	-0.173952	-0.014740

PCHS

Center Number	Atomic Number	Atomic Type	Coordinates (Angstroms) X	Y	Z
1	6	0	0.731291	1.113223	0.045753
2	6	0	1.323304	0.799033	-1.166977
3	6	0	2.755013	0.765911	-1.109738
4	6	0	3.259331	1.049537	0.150918
5	6	0	1.418906	-0.412583	-3.384593
6	1	0	1.559043	-1.420387	-2.970611

7	1	0	0.889783	-0.531744	-4.336716
8	6	0	2.783529	0.245361	-3.600831
9	1	0	2.632821	1.262018	-3.988459
10	1	0	3.363713	-0.295435	-4.356776
11	6	0	0.566954	0.430630	-2.425258
12	1	0	-0.357061	-0.094641	-2.164392
13	1	0	0.255120	1.346231	-2.947438
14	6	0	3.586595	0.304706	-2.292342
15	1	0	4.459190	0.947935	-2.427235
16	1	0	3.986461	-0.695131	-2.068704
17	6	0	-0.685155	1.073272	0.399426
18	6	0	-1.298897	0.347794	1.407838
19	6	0	-2.730308	0.396156	1.353431
20	6	0	-0.568094	-0.499670	2.427126
21	6	0	-3.212819	1.155519	0.297665
22	6	0	-3.589194	-0.444364	2.280073
23	6	0	-1.460800	-1.608916	3.001284
24	1	0	0.336615	-0.923073	1.979899
25	1	0	-0.224757	0.136541	3.255138
26	6	0	-2.799481	-1.027860	3.461818
27	1	0	-4.435745	0.134824	2.655917
28	1	0	-4.027946	-1.267641	1.697595
29	1	0	-1.638133	-2.377538	2.236799
30	1	0	-0.944523	-2.101776	3.832705
31	1	0	-2.610998	-0.241168	4.204885
32	1	0	-3.406933	-1.791129	3.960760
33	16	0	-1.885289	1.884637	-0.583838
34	16	0	1.955283	1.443850	1.253197
35	-2	0	7.849805	-0.228699	0.332235

PCHSe

Center Number	Atomic Number	Atomic Type	Coordinates (Angstroms) X	Y	Z
1	6	0	0.752077	1.418775	0.013565
2	6	0	1.376510	0.815661	-1.067996
3	6	0	2.828207	0.773174	-1.015983
4	6	0	3.406583	1.355302	0.102459
5	6	0	1.437111	-0.879753	-2.973622
6	1	0	1.561599	-1.778955	-2.350839
7	1	0	0.898035	-1.185969	-3.880576
8	6	0	2.824273	-0.311889	-3.333403
9	1	0	2.695244	0.602569	-3.932165
10	1	0	3.386167	-1.024849	-3.952311
11	6	0	0.605142	0.179304	-2.217334
12	1	0	-0.330488	-0.253923	-1.842792
13	1	0	0.313030	0.966077	-2.930819
14	6	0	3.635477	0.002896	-2.057001
15	1	0	4.547590	0.553218	-2.314953
16	1	0	3.972608	-0.945267	-1.607471
17	6	0	-0.683553	1.421019	0.300781
18	6	0	-1.341415	0.631994	1.232330
19	6	0	-2.793206	0.665799	1.175481
20	6	0	-0.607280	-0.264735	2.221647
21	6	0	-3.337761	1.496056	0.206719
22	6	0	-3.640553	-0.272311	2.029847

23	6	0	-1.490114	-1.420134	2.742417
24	1	0	0.310849	-0.651983	1.762963
25	1	0	-0.285283	0.342332	3.082692
26	6	0	-2.855420	-0.880898	3.212743
27	1	0	-4.533038	0.247639	2.395880
28	1	0	-4.008618	-1.091419	1.390634
29	1	0	-1.646655	-2.165111	1.946958
30	1	0	-0.972333	-1.930774	3.565991
31	1	0	-2.694119	-0.117973	3.989578
32	1	0	-3.452309	-1.682879	3.668424
33	34	0	2.056454	2.141996	1.244254
34	34	0	-1.946500	2.444513	-0.746984
35	-2	0	8.152083	-0.215546	0.293617

PCHTe

Center Number	Atomic Number	Atomic Type	Coordinates (Angstroms) X	Y	Z
1	6	0	0.763839	1.642374	-0.013883
2	6	0	1.442531	0.843599	-0.921810
3	6	0	2.900268	0.809864	-0.847728
4	6	0	3.521026	1.593013	0.112713
5	6	0	1.529006	-1.147223	-2.537401
6	1	0	1.642792	-1.941203	-1.782926
7	1	0	0.999054	-1.588859	-3.392624
8	6	0	2.922203	-0.644377	-2.960886
9	1	0	2.806800	0.166454	-3.696281

10	1	0	3.492004	-1.445608	-3.451616
11	6	0	0.691943	0.016576	-1.964591
12	1	0	-0.248095	-0.353284	-1.538414
13	1	0	0.405205	0.680677	-2.796141
14	6	0	3.708526	-0.138699	-1.732729
15	1	0	4.639480	0.348380	-2.047383
16	1	0	4.013212	-1.009465	-1.129281
17	6	0	-0.693902	1.663441	0.161045
18	6	0	-1.407699	0.814209	0.993362
19	6	0	-2.865651	0.848758	0.918514
20	6	0	-0.694438	-0.132187	1.958625
21	6	0	-3.450935	1.745414	0.038408
22	6	0	-3.713338	-0.145627	1.710900
23	6	0	-1.582401	-1.298922	2.441653
24	1	0	0.224536	-0.510994	1.495892
25	1	0	-0.371830	0.447614	2.838642
26	6	0	-2.956365	-0.774271	2.899887
27	1	0	-4.635066	0.339569	2.054074
28	1	0	-4.032431	-0.955008	1.033605
29	1	0	-1.725676	-2.028558	1.629574
30	1	0	-1.074153	-1.825022	3.261548
31	1	0	-2.814243	-0.027133	3.695639
32	1	0	-3.560142	-1.587717	3.325479
33	52	0	-1.991620	2.899874	-0.949313
34	52	0	2.109613	2.712128	1.206007
35	-2	0	8.403868	-0.197763	0.396558

Checkcif report provided by IUCr

No syntax errors found.
Please wait while processing ....

CIF dictionary
Interpreting this report

Datablock: cptditms

Bond precision: C-C = 0.0089 A Wavelength=0.71073

Cell: a=6.0030(4) b=13.0295(10) c=13.9564(10)
alpha=101.251(4) beta=92.850(4) gamma=91.986(4)

Temperature: 296 K

	Calculated	Reported
Volume	1068.22(13)	1068.21(13)
Space group	P -1	P -1
Hall group	-P 1	-P 1
Moiety formula	C17 H32 O2 S Si2	C17 H32 O2 S Si2
Sum formula	C17 H32 O2 S Si2	C17 H32 O2 S Si2
Mr	356.67	356.67
Dx,g cm-3	1.109	1.109
Z	2	2
Mu (mm-1)	0.268	0.268
F000	388.0	388.0
F000'	388.69	
h,k,lmax	7,15,16	7,15,16
Nref	3764	3764
Tmin,Tmax	0.902,0.940	0.902,0.940
Tmin'	0.889	

Correction method= # Reported T Limits: Tmin=0.902 Tmax=0.940
AbsCorr = MULTI-SCAN

Data completeness= 1.000 Theta(max)= 25.000

R(reflections)= 0.0720(2893) wR2(reflections)= 0.2104(3764)

S = 1.311 Npar= 207

The following ALERTS were generated. Each ALERT has the format
test-name_ALERT_alert-type_alert-level.
Click on the hyperlinks for more details of the test.

Alert level C

ABSTY02_ALERT_1_C An _exptl_absorpt_correction_type has been given without
a literature citation. This should be contained in the
_exptl_absorpt_process_details field.
Absorption correction given as multi-scan
PLAT242_ALERT_2_C Low 'MainMol' Ueq as Compared to Neighbors of Si1 Check
PLAT242_ALERT_2_C Low 'MainMol' Ueq as Compared to Neighbors of Si2 Check
PLAT340_ALERT_3_C Low Bond Precision on C-C Bonds .... 0.00889 Ang.

Alert level G

PLAT005_ALERT_5_G No Embedded Refinement Details Found in the CIF Please Do !
PLAT066_ALERT_1_G Predicted and Reported Tmin&Tmax Range Identical ? Check
PLAT093_ALERT_1_G No s.u.'s on H-positions, Refinement Reported as mixed Check
PLAT154_ALERT_1_G The s.u.'s on the Cell Angles are Equal ..(Note) 0.004 Degree
PLAT899_ALERT_4_G SHELXL-97 is Deprecated and Succeeded by SHELXL 2019/3 Note

0 ALERT level A = Most likely a serious problem - resolve or explain
0 ALERT level B = A potentially serious problem, consider carefully
4 ALERT level C = Check. Ensure it is not caused by an omission or oversight
5 ALERT level G = General information/check it is not something unexpected

4 ALERT type 1 CIF construction/syntax error, inconsistent or missing data
2 ALERT type 2 Indicator that the structure model may be wrong or deficient
1 ALERT type 3 Indicator that the structure quality may be low
1 ALERT type 4 Improvement, methodology, query or suggestion
1 ALERT type 5 Informative message, check

It is advisable to attempt to resolve as many as possible of the alerts in all categories. Often the minor alerts point to easily fixed oversights, errors and omissions in your CIF or refinement strategy, so attention to these fine details can be worthwhile. In order to resolve some of the more serious problems it may be necessary to carry out additional measurements or structure refinements. However, the purpose of your study may justify the reported deviations and the more serious of these should normally be commented upon in the discussion or experimental section of a paper or in the "special_details" fields of the CIF. checkCIF was carefully designed to identify outliers and unusual parameters, but every test has its limitations and alerts that are not important in a particular case may appear. Conversely, the absence of alerts does not guarantee there are no aspects of the results needing attention. It is up to the individual to critically assess their own results and, if necessary, seek expert advice.

Publication of your CIF in IUCr journals

A basic structural check has been run on your CIF. These basic checks will be run on all CIFs submitted for publication in IUCr journals (*Acta Crystallographica*, *Journal of Applied Crystallography*, *Journal of Synchrotron Radiation*); however, if you intend to submit to *Acta Crystallographica Section C* or *E* or *IUCrData*, you should make sure that full publication checks are run on the final version of your CIF prior to submission.

Publication of your CIF in other journals

Please refer to the *Notes for Authors* of the relevant journal for any special instructions relating to CIF submission.

PLATON version of 06/07/2023; check.def file version of 30/06/2023

Datablock cptditms - ellipsoid plot

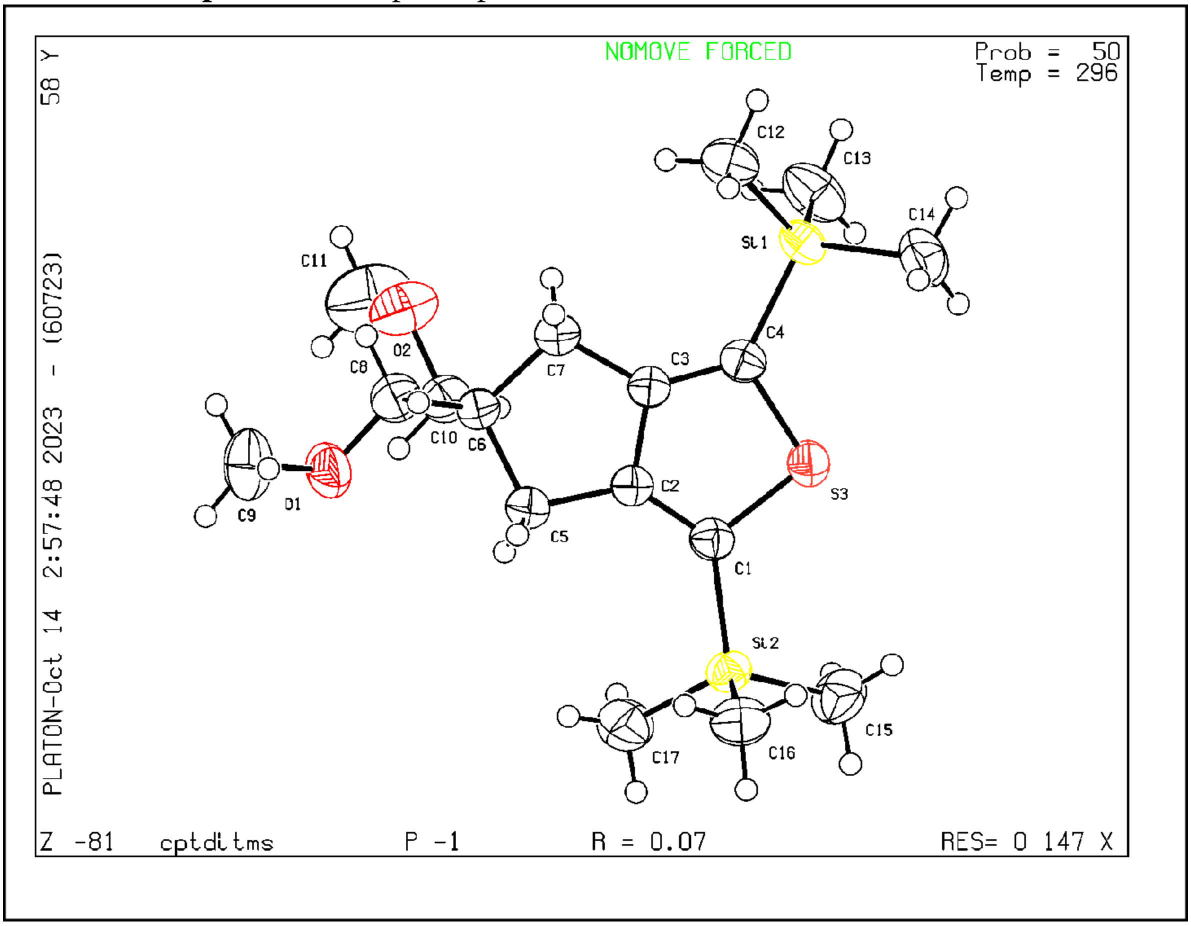

[Download CIF editor (publCIF) from the IUCr](#) [Download CIF editor (enCIFer) from the CCDC](#)[Test a new CIF entry](#)
